# Synthesis and Biological Evaluation of New *N*-Acyl-α-amino Ketones and 1,3-Oxazoles Derivatives

**DOI:** 10.3390/molecules26165019

**Published:** 2021-08-19

**Authors:** Theodora-Venera Apostol, Luminita Gabriela Marutescu, Constantin Draghici, Laura-Ileana Socea, Octavian Tudorel Olaru, George Mihai Nitulescu, Elena Mihaela Pahontu, Gabriel Saramet, Cristian Enache-Preoteasa, Stefania-Felicia Barbuceanu

**Affiliations:** 1 Faculty of Pharmacy, “Carol Davila” University of Medicine and Pharmacy, 6 Traian Vuia Street, 020956 Bucharest, Romania; theodora.apostol@umfcd.ro (T.-V.A.); laura.socea@umfcd.ro (L.-I.S.); george.nitulescu@umfcd.ro (G.M.N.); elena.pahontu@umfcd.ro (E.M.P.); gabriel.saramet@umfcd.ro (G.S.); stefania.barbuceanu@umfcd.ro (S.-F.B.); 2 Department of Botany and Microbiology, Faculty of Biology & Research Institute of the University of Bucharest (ICUB), University of Bucharest, 060101 Bucharest, Romania; 3 “Costin D. Nenițescu” Centre of Organic Chemistry, Romanian Academy, 202 B Splaiul Independenței, 060023 Bucharest, Romania; cst_drag@yahoo.com; 4 National Phytosanitary Laboratory, 11 Voluntari Boulevard, 077190 Voluntari, Romania; cristian.enache@anfdf.ro

**Keywords:** *N*-acyl-α-amino acid, 4-isopropyl-1,3-oxazol-5(4*H*)-one, *N*-acyl-α-amino ketone, 4-isopropyl-1,3-oxazole, antimicrobial activity, antibiofilm agents, toxicity

## Abstract

In order to develop novel bioactive substances with potent activities, some new valine-derived compounds incorporating a 4-(phenylsulfonyl)phenyl fragment, namely, acyclic precursors from *N*-acyl-α-amino acids and *N*-acyl-α-amino ketones classes, and heterocycles from the large family of 1,3-oxazole-based compounds, were synthesized. The structures of the new compounds were established using elemental analysis and spectral (UV-Vis, FT-IR, MS, NMR) data, and their purity was checked by reversed-phase HPLC. The newly synthesized compounds were evaluated for their antimicrobial and antibiofilm activities, for toxicity on *D. magna*, and by in silico studies regarding their potential mechanism of action and toxicity. The 2-aza-3-isopropyl-1-[4-(phenylsulfonyl)phenyl]-1,4-butanedione **4b** bearing a *p*-tolyl group in 4-position exhibited the best antibacterial activity against the planktonic growth of both Gram-positive and Gram-negative strains, while the *N*-acyl-α-amino acid **2** and 1,3-oxazol-5(4*H*)-one **3** inhibited the *Enterococcus faecium* biofilms. Despite not all newly synthesized compounds showing significant biological activity, the general scaffold allows several future optimizations for obtaining better novel antimicrobial agents by the introduction of various substituents on the phenyl moiety at position 5 of the 1,3-oxazole nucleus.

## 1. Introduction

Many heterocyclic compounds are very important in medicinal chemistry since they exhibit remarkable and various pharmacological activities, being present as active substances in the composition of numerous potent drugs.

**Figure 1:**
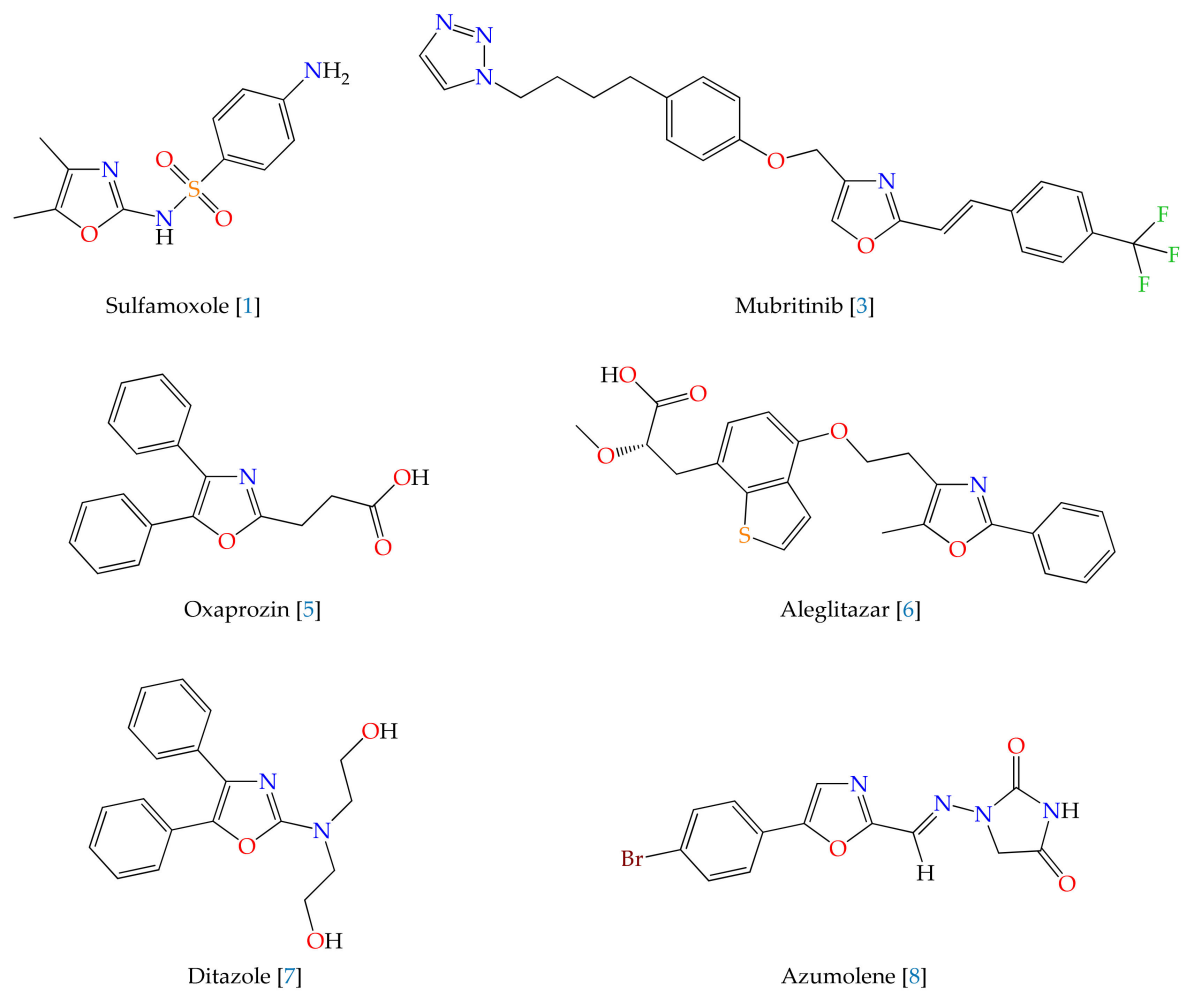
Structures of some representative bioactive compounds containing 1,3-oxazole moiety.

Among them, the synthetic heterocycles containing 1,3-oxazole nucleus have a wide range of biological activities, such as antimicrobial (e.g., sulfamoxole, a chemotherapeutic agent from the sulfonamides group) [1,2], anticancer (e.g., mubritinib, a tyrosine kinase inhibitor) [3], analgesic, antipyretic, anti-inflammatory (e.g., oxaprozin) [4,5], anti-diabetic (e.g., aleglitazar, a dual PPARα/γ agonist from glitazar class used in the treatment of type 2 diabetes) [6], antithrombotic (e.g., ditazole, an inhibitor of platelet aggregation) [7], and skeletal muscle relaxant (e.g., azumolene) [8] actions. The structures of the representative bioactive compounds sharing the 1,3-oxazole scaffold are presented in Figure 1.

It is also worth noting that many biologically active heterocyclic compounds which contain 1,3-oxazole ring are biosynthesized by marine invertebrates and microorganisms, such as muscoride A (an antibacterial agent) [9,10], virginiamycin M_2_ (from group A streptogramin antibiotics) [11], ulapualide A (with antifungal activity) [10], diazonamide A (an anticancer agent that inhibits tubulin polymerization) [10,12,13], hennoxazole A (with antiviral and analgesic effect) [10], and texaline (an antimycobacterial alkaloid) [14]. In addition, some saturated 1,3-oxazol-5(4*H*)-ones are reported to present antiviral [15] and antimicrobial (e.g., jadomycin B) [16] properties.

The literature survey on intermediates used in the synthesis of heterocycles from 1,3-oxazoles class (namely, *N*-acyl-α-amino acid and *N*-acyl-α-amino ketone derivatives) revealed that they are also endowed with a broad spectrum of therapeutic activities. A large number of representatives of *N*-acyl-α-amino acids have anticancer (e.g., methotrexate) [17], mucolytic (e.g., *N*-acetyl cysteine) [18], antihypertensive (e.g., angiotensin-converting enzyme inhibitors: captopril, enalapril, lisinopril) [19], antianemic (e.g., folic acid) [20], anti-ulcer (e.g., benzotript) [21] effects, and are specific antidotes in acute intoxications [22,23]. The *N*-acyl-α-amino ketones display antiviral (e.g., rupintrivir) [24], anti-inflammatory [25], and antithrombotic [26] actions.

Derivatives of the above classes were linked with a fragment derived from diphenyl sulfone with the purpose of obtaining new compounds with potent biological properties. The choice of this pharmacophore center is justified, on the one hand, by the fact that numerous diaryl sulfones (e.g., dapsone, acedapsone, glucosulfone, sulfoxone, thiazosulfone) were found to possess antimicrobial, antimalarial, antioxidant, anticancer properties [27,28,29,30,31,32,33] and, on the other hand, by data from the literature indicating that it was incorporated into various heterocyclic systems with biological value [34,35].

**Figure 2:**
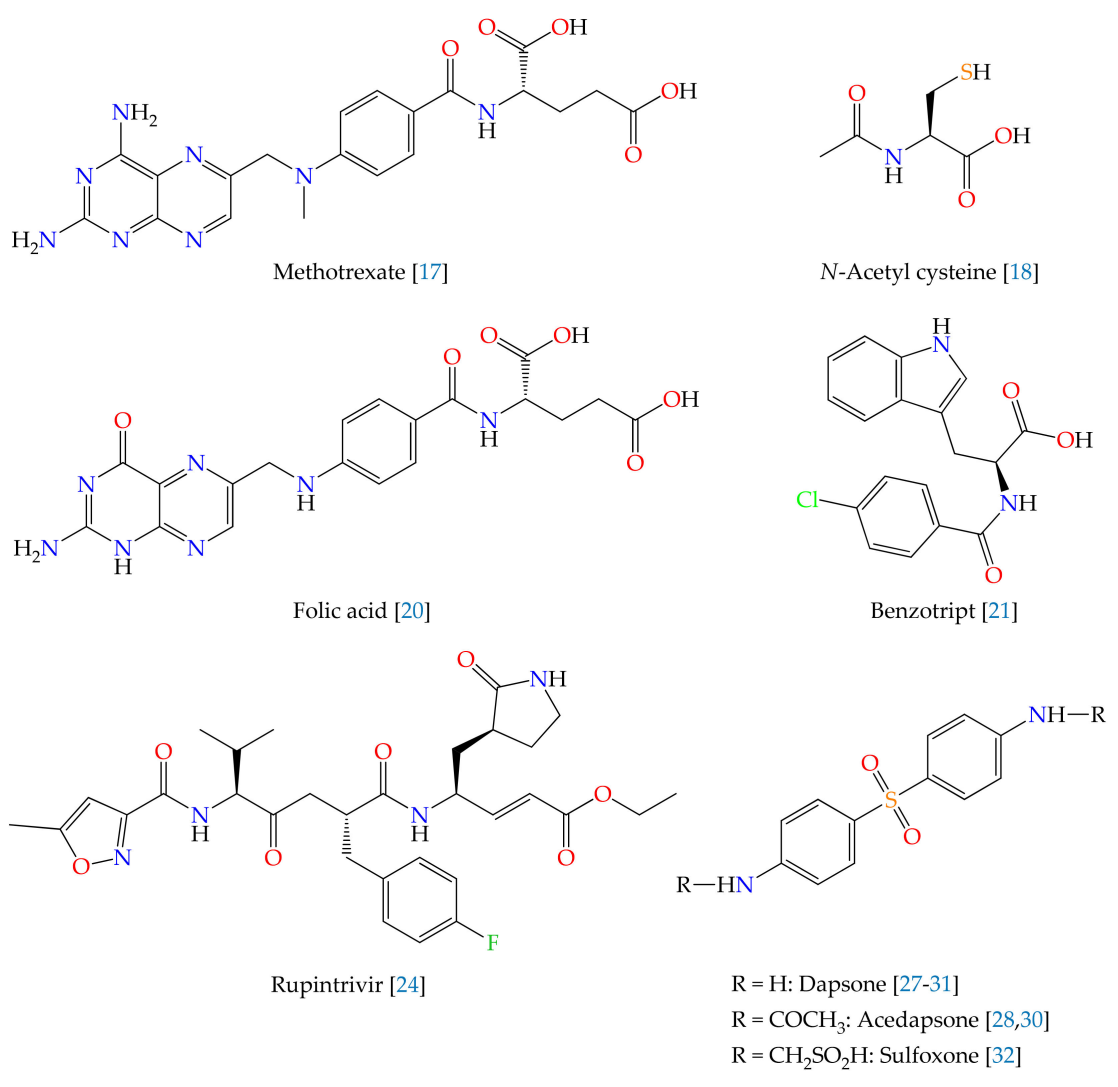
Structures of some representative biologically active compounds from *N*-acyl-α-amino acids, *N*-acyl-α-amino ketones, and diaryl sulfones classes.

The structures of some representative bioactive compounds from *N*-acyl-α-amino acids, *N*-acyl-α-amino ketones, and diaryl sulfones classes are shown in Figure 2.

Our findings showed that 1,3-oxazole-based compounds could be potent agents regarding the antimicrobial activity. For example, Tipparaju et al. reported the first synthesis and biological evaluation of the natural antibiotic 2-{3-hydroxy-2-[(3-hydroxypyridine-2-carbonyl)amino]phenyl}-1,3-benzoxazole-4-carboxylic acid (A-33853) and of a number of its analogues, and discovered novel antileishmanial chemotypes [36]. In addition, recently, other new benzoxazole derivatives were developed as antiprotozoal agents [37] and a series of novel pyridyl–oxazole carboxamides were evaluated against fungi and displayed good fungicidal activities [38]. Further, four previously undescribed new biologically active secondary metabolites, 2,5-disubstituted 1,3-oxazole-4-carboxylic acid derivatives (named as macrooxazoles A–D) were isolated from the plant pathogenic fungus *Phoma macrostoma* and assessed for their antimicrobial, cytotoxic, and antibiofilm activities [39].

The aim of the present work was to continue our previous research on 1,3-oxazole derivatives [40,41,42,43,44,45], by focusing on the biological evaluation of newly synthesized *N*-acyl-α-amino acid, 1,3-oxazol-5(4*H*)-one, *N*-acyl-α-amino ketone, and 1,3-oxazole analogs derived from valine containing the 4-(phenylsulfonyl)phenyl substituent.

## 2. Results

### 2.1. Chemistry

#### 2.1.1. Chemical Synthesis

**Scheme 1:**
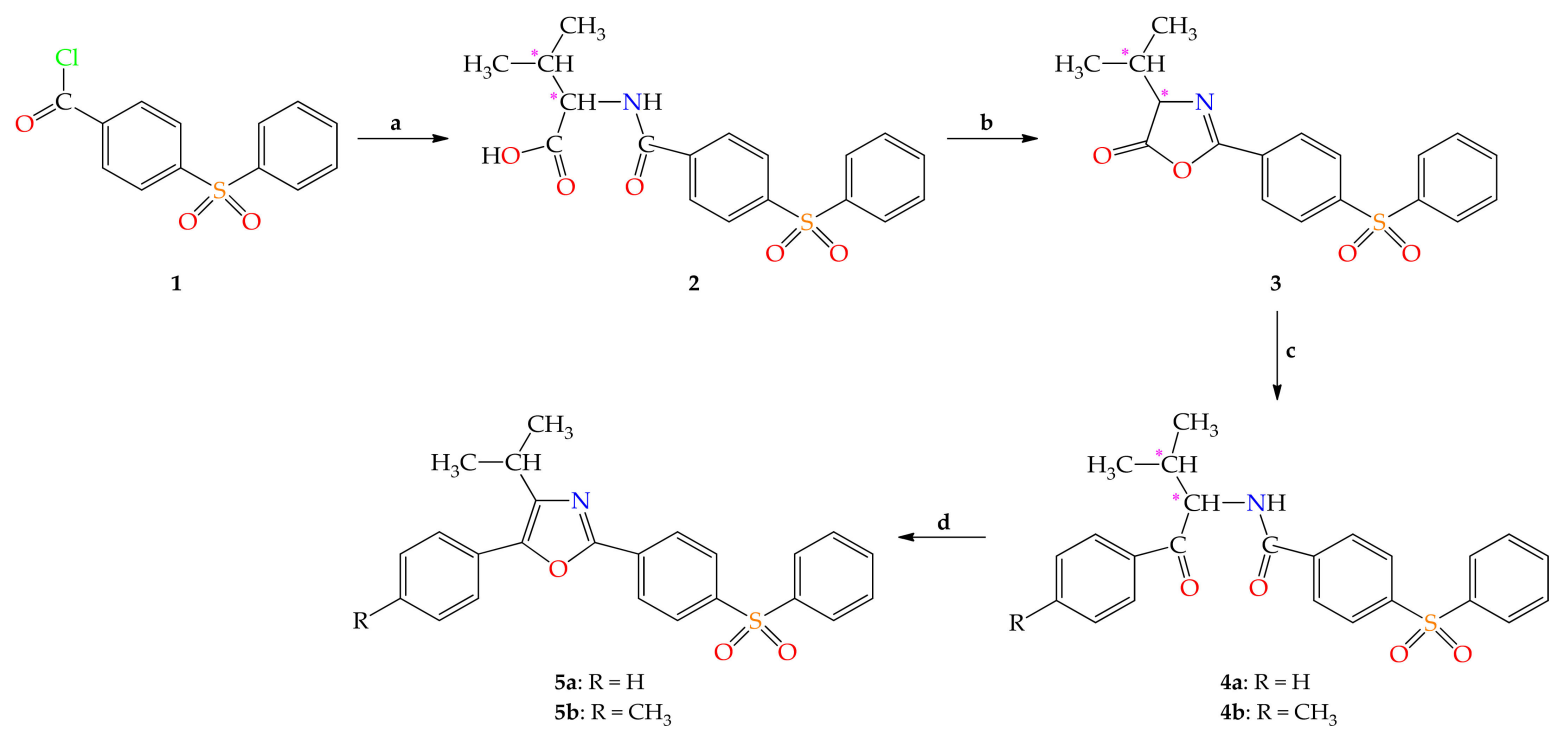
Synthesis of the new compounds 2–5. Reagents and conditions: (**a**) (**i**) *L*-valine/NaOH, CH_2_Cl_2_, 0–5 °C, 30 min; room temperature (r.t.), 1 h; (**ii**) HCl (89%); (**b**) ClCO_2_C_2_H_5_/4-methylmorfoline, CH_2_Cl_2_, r.t., 30 min (90%); (**c**) C_6_H_5_-R (R = H or CH_3_)/AlCl_3_, r.t., 20 h (**4a**: 80%, **4b**: 74%); (**d**) POCl_3_, reflux, 4 h (**5a**: 79%, **5b**: 76%).

The new compounds **2**–**5** were prepared using the multi-step synthetic strategy presented in Scheme 1.

Synthesis of the new compounds started from the known acyl chloride **1** [40,46], which was used for the *N*-acylation of *L*-valine (*L*-2-amino-3-methylbutanoic acid, (*S*)-2-amino-3-methylbutanoic) to new 3-methyl-2-[4-(phenylsulfonyl)benzamido]butanoic acid **2**. Intramolecular cyclodehydration of compound **2**, using ethyl chloroformate in the presence of 4-methylmorpholine (in the molar ratio of 1:1:1), in anhydrous dichloromethane, at room temperature, led to 4-isopropyl-2-[4-(phenylsulfonyl)phenyl]-1,3-oxazol-5(4*H*)-one **3**. Friedel–Crafts acylation, catalyzed by aluminum trichloride of aromatic hydrocarbons (benzene, toluene) with 2-aryl-4-isopropyl-1,3-oxazol-5(4*H*)-one **3** yielded the *N*-(1-aryl-3-methyl-1-oxobutan-2-yl)-4-(phenylsulfonyl)benzamides **4a,b**. These intermediates underwent Robinson–Gabriel cyclization using phosphoryl trichloride at reflux with the preparation of 5-aryl-4-isopropyl-2-[4-(phenylsulfonyl)phenyl]-1,3-oxazoles **5a,b**. Elemental and spectral (UV-Vis, FT-IR, MS, ^1^H- and ^13^C-NMR) analyses were used to characterize the new compounds.

#### 2.1.2. Spectral Characterization

##### UV-Vis Spectral Data

The UV-Vis spectra of new compounds **2**–**5** showed the E band at *λ*_max_ = 202.6 nm and the B band in the range of 241.0–252.0 nm. Furthermore, 2,5-diaryl-4-isopropyl-1,3-oxazoles **5a,b** spectra presented a third absorption band at a longer wavelength: 331.3 (**5a**) or 336.6 (**5b**) nm, due to the appearance of the 1,3-oxazole chromophore, which determined the extension of the π electrons conjugation.

##### IR Spectral Data

For acyclic intermediates **2** and **4a,b**, a characteristic absorption band due to N-H stretching vibration, *ν*(N-H), was registered in the range of 3302–3424 cm^−1^. In the IR spectrum of *N*-acyl-α-amino acid **2**, the peak due to carbonyl valence vibration, *ν*(O=C-C), was recorded at 1720 cm^−1^, and amidic carbonyl absorption, *ν*(O=C-N), at 1674 cm^−1^. These two carbonyl absorption bands are overlapped in the case of *N*-acyl-α-amino ketones **4a,b**, as suggested by the single very strong peak present in their spectra at 1657 (**4b**) or 1662 (**4a**) cm^−1^. Representative bands for the *N*-acyl-*L*-valine **2** associated by hydrogen bonds are also: a strong, broad absorption peak between 2500 and 3300 cm^−1^, and two medium broad bands at 2686 and 2609 cm^−1^ due to O-H stretching vibration, *ν*(O-H).

The IR spectra of heterocyclic compounds **3** and **5a,b** differ significantly from the corresponding spectra of their open-chain precursors (*N*-acyl-α-amino acid **2** and *N*-acyl-α-amino ketones **4a,b**) and this demonstrates that intramolecular cyclocondensation reactions occurred. In the IR spectrum of saturated azlactone **3**, the band due to carbonyl valence vibration, *ν*(C=O), is shifted at a higher wavenumber (1827 cm^−1^), compared with the corresponding absorption band in the compound **2** spectrum. The IR spectra of heterocycles **3** and **5a,b** presented a peak due to C=N valence vibration, *ν*(C=N), at 1649 cm^−1^ (**3**), and 1597 (**5b**) or 1603 (**5a**) cm^−1^. Moreover, the band due to C-O-C symmetrical stretching vibration, *ν*_sym_(C-O-C), was registered at 1037 cm^−1^ in the 1,3-oxazol-5(4*H*)-one **3** spectrum, and at 1095 (**5b**) or 1098 (**5a**) cm^−1^ in 1,3-oxazoles spectra. In the spectrum of **3**, the peak due to C-O-C asymmetrical stretching vibration, *ν*_as_(C-O-C), was observed at 1244 cm^−1^, while in compounds **5a,b** spectra, it is overlapped with the asymmetric sulfonyl stretch, ν_as_(SO_2_) from 1292 (**5b**) and 1294 cm^−1^ (**5a**), respectively.

##### NMR Spectral Data

The NMR spectral data also proved the structures of the new compounds (Supplementary Materials).

##### ^1^H-NMR Spectral Data

**Figure 3:**
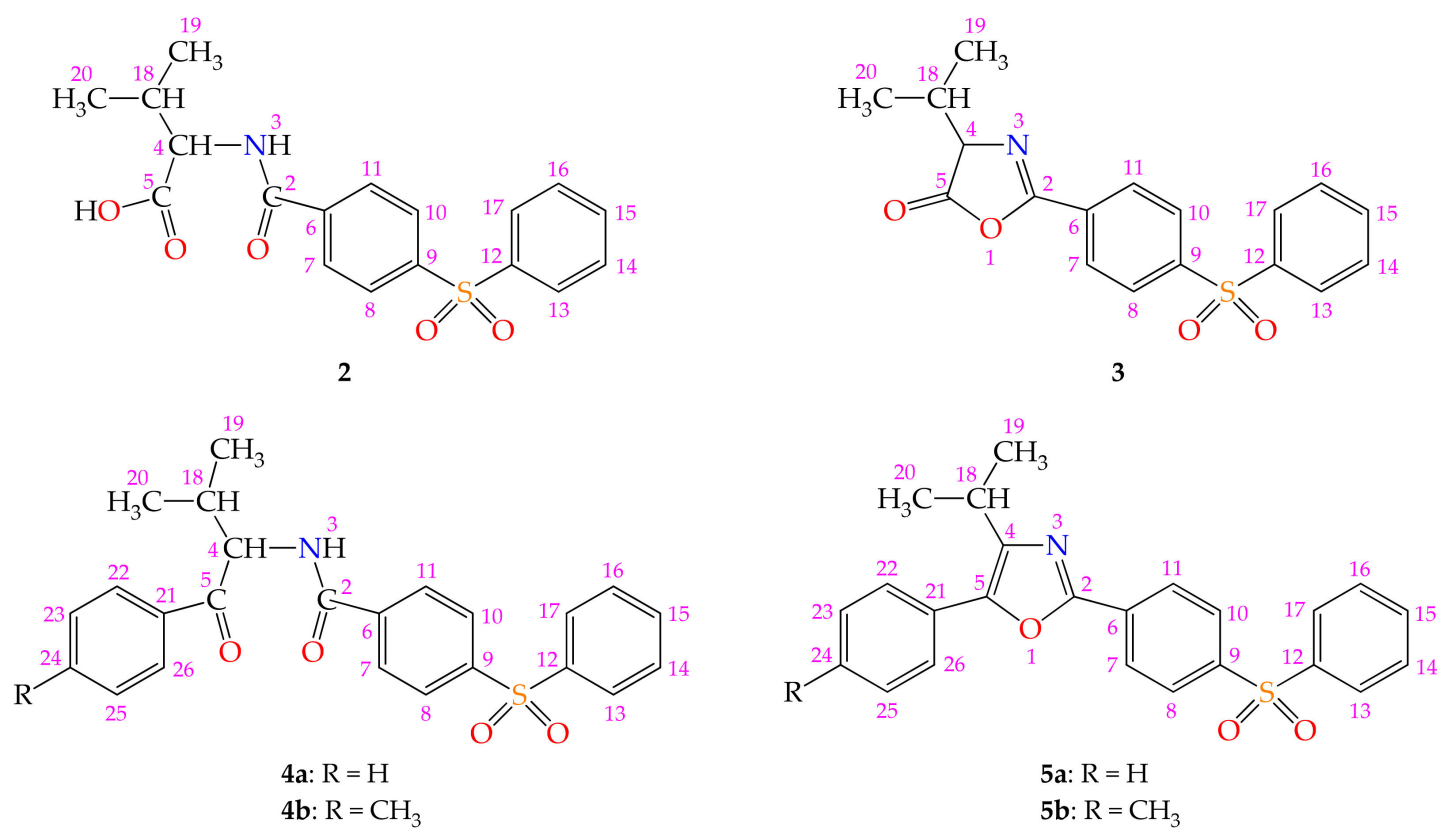
Structures of compounds **2**–**5** with the numbering of atoms (for NMR assignments).

The numbering of atoms used for assigning the NMR signals of the compounds **2**–**5** is presented in Figure 3.

In ^1^H-NMR spectra of new compounds **2** and **4a,b**, for the deshielded proton of NH group, respectively H-3, the signal was registered as a doublet at *δ*_H_ = 8.38 ppm (**2**) and 8.96 (**4b**) or 8.99 (**4a**) ppm, due to the vicinal coupling to H-4. In the ^1^H-NMR spectrum of the *N*-acyl-α-amino acid **2**, a doublet of doublets signal at 4.26 ppm was assigned to the H-4 proton, coupled to the proton of the NH group and the H-18 methine proton. In the case of *N*-acyl-α-amino ketones **4a,b**, the H-4 proton signal appeared as a triplet at 5.35 (**4b**) or 5.38 (**4a**) ppm, due to the coupling to H-3 and H-18. In the ^1^H-NMR spectra of acyclic intermediates **2** and **4a,b**, for the isopropyl group, an octet or multiplet signal was recorded in the 2.12–2.26 ppm range due to the proton of the methine group (H-18) and two strongly shielded doublet signals at *δ*_H_ = 0.89 (**2**, **4b**) or 0.90 (**4a**) ppm and in the interval: 0.90–0.92 ppm, respectively, corresponding to the H-19 and H-20 nonequivalent protons from the two methyl groups.

A proof for the intramolecular cyclocondensations of precursors **2** and **4a,b** is represented by the absence in ^1^H-NMR spectra of heterocyclic compounds **3** and **5a,b** of the signal assigned to the NH proton.

In the case of 4-isopropyl-1,3-oxazol-5(4*H*)-one **3**, the H-4 signal was recorded at 4.32 ppm as a doublet due to coupling to the adjacent methine proton and the signal of H-18 at 2.39 ppm as a heptet of doublets, this proton being coupled to H-19 and H-20 protons (with ^3^*J* = 6.9 Hz) and H-4 (with ^3^*J* = 4.7 Hz). In the ^1^H-NMR spectra of 1,3-oxazoles **5a,b**, for the isopropyl group, a heptet signal appeared at *δ*_H_ = 3.26 (**5b**) or 3.28 (**5a**) ppm due to the H-18 proton (shifted downfield compared to the corresponding proton signal registered for *N*-acyl-α-amino ketones **4a,b**), and a strongly shielded doublet at *δ*_H_ = 1.35 (**5b**) or 1.36 (**5a**) ppm, corresponding to protons of the two CH_3_ groups.

##### ^13^C-NMR Spectral Data

In the ^13^C-NMR spectrum of *N*-acyl-α-amino acid **2**, the signal due to the C-4 atom was observed at a chemical shift value of 58.43 ppm. The isopropyl group is highlighted by the presence of the carbon atom of the methine group for which a signal was recorded at 29.48 ppm and by the presence of nonequivalent carbon atoms of methyl groups, which showed two signals at *δ*_C_ = 18.61 and 19.24 ppm, respectively.

The C-4 signal was shifted downfield with 12.59 ppm after cyclodehydration of compound **2** to saturated azlactone **3**. Further, in the case of 1,3-oxazol-5(4*H*)-one **3**, the C-2 atom resonated at 160.44 ppm, being more shielded with 5.43 ppm than the corresponding carbon of intermediate **2**, and the C-5 at 177.05 ppm, being shifted downfield with 4.24 ppm than the corresponding atom of **2**.

In the ^13^C-NMR spectra of 1,3-oxazoles **5a,b**, the C-4 signal registered at *δ*_C_ = 143.56 (**5b**) or 144.15 (**5a**) ppm was more deshielded with ≈84.70 ppm by comparison of the signal of the corresponding carbon atom of **4a,b** observed at *δ*_C_ = 59.09 (**4b**) or 59.22 (**4a**) ppm and this is an additional indication that the formation of the 1,3-oxazole ring took place. The signal attributed to C-2 of 1,3-oxazoles **5a,b** was recorded at *δ*_C_ = 157.65 (**5b**) or 157.93 (**5a**) ppm, while the corresponding carbon signal of *N*-acyl-α-amino ketones **4a,b** appeared at *δ*_C_ = 165.61 (**4b**) or 165.65 (**4a**) ppm. The C-5 atom of 1,3-oxazoles **5a,b** resonated at *δ*_C_ = 145.41 (**5a**) or 145.60 (**5b**) ppm, whereas the corresponding carbon of **4a,b** at *δ*_C_ = 198.66 (**4b**) or 199.22 (**4a**) ppm, revealing an upfield shift for this carbon atom, as a confirmation that the intramolecular cyclocondensation occurred.

##### Mass Spectral Data

The mass spectra recorded by the L-ESI-MS/MS technique had an additional contribution to the elucidation of the structures of compounds **2**, **3**, **4a**, and **5a**.

In this study was used the ability of ionization at atmospheric pressure of electrospray ionization (ESI) type for amino acid derivatives analysis. Further, a special advantage of the ESI source is that negative ions can also be analyzed.

In our experiments, compounds **2** and **4a**, dissolved in methanol/water with 0.1% ammonium carbonate (9:1, *v*/*v*), were ionized positively and negatively, respectively. For compounds **3** and **5a**, methanol/water (containing 0.1% ammonium formate and 1% formic acid) 9:1 (*v*/*v*) was used as the solvent mixture. After evaporation of the solvents, the ions obtained were introduced into the mass spectrometer.

ESI is considered a mild source of ionization. Consequently, the mass spectra of these compounds are very simple and consist mainly of the protonated molecular ion [M + H]^+^ for positive ionization or the deprotonated molecular ion [M−H]^-^ for negative ionization. Non-covalent dipole–dipole interactions can be highlighted by using ESI so that in the compounds spectra appeared combinations like [2M + H]^+^ and [2M−H]^−^, respectively (e.g., for **4a**).

Valuable structural information can be obtained from the fragmentation by the collision of pseudo-molecular ions with an inert gas (argon), as fragmentation in positive-ion mode can be different from fragmentation in negative-ion mode. In the positive ionization mode, the first fragmentation occurred with water (for **2**), carbon monoxide (for **3**), or methane loss (for **5a**), and in the negative ionization mode with carbon dioxide (for **2**) or isopropyl radical loss (for **4a**). Protonated and/or deprotonated molecular ions and the main fragments of these compounds are reported in the Materials and Methods section.

In the mass spectrum of 1,3-oxazol-5(4*H*)-one **3** which was also achieved by GC-EI-MS analysis, the molecular ion, [M]^+^, being unstable, did not show a signal. 1,3-Oxazol-5(4*H*)-one **3** was first fragmented at the side chain from 4-position with the removal of a propene molecule and the formation of cation-radical with *m*/*z* = 301, which is the base peak (BP). Other main fragments of **3** are indicated in Materials and Methods.

### 2.2. Antimicrobial Activity Assessment

#### 2.2.1. Qualitative Analysis of the Antimicrobial Activity

The results of the qualitative analysis of the antimicrobial activity of the newly synthesized compounds showed that the majority of the compounds did not produce growth inhibition zones, except for compounds **2** and **3**, which inhibited the growth of the Gram-positive strain *Enterococcus faecium* E5, producing a growth inhibition zone of 20 and 17 mm, respectively.

#### 2.2.2. Investigation of the Influence of the Tested Compounds on the Antibiotic Susceptibility Spectrum of *Enterococcus faecium* E5

*E. faecium* is increasingly identified in nosocomial infections and it has rapidly adapted to newer anti-gram-positive agents (e.g., linezolid, quinupristin/dalfopristin, daptomycin, tigecycline) [47]. Therefore, the development of new drugs to combat these recalcitrant microorganisms is needed. For compounds **2** and **3** only, which proved to be active in the qualitative disk diffusion assay against *E. faecium* E5, their influence on the antibiotic susceptibility spectrum of this strain was evaluated. Antibiotic susceptibility tests were performed and interpreted according to the CLSI. The diameters of growth inhibition zones (mm) are shown in Table 1. Kirby–Bauer disk diffusion tests showed that *E. faecium* E5 was resistant to penicillin and susceptible to ampicillin, linezolid, and vancomycin. Regarding the effects of compounds **2** and **3**, no changes in the *E. faecium* E5 strain susceptibility to the tested antibiotics were determined after cultivation in the presence of subinhibitory concentrations of the two compounds, suggesting both a different mechanism of action and a low selective pressure for resistance occurrence.

#### 2.2.3. Quantitative Evaluation of Antimicrobial and Antibiofilm Activities

The urgent need for novel antimicrobial agents persists, as the emergence of multi-drug pathogens is both unpredictable and inevitable. Therefore, the α-amino ketones and 1,3-oxazoles derivatives received significant importance due to their wide spectrum of biological applications across synthetic and medicinal chemistry [48,49]. The newly synthesized derivatives were screened for their antimicrobial activity using the two-fold serial microdilution method, following the CLSI guidelines. Additionally, the potential of the compounds to prevent initial cell attachment was investigated through the biofilm inhibition assay. The minimal inhibitory concentration (MIC) and the minimal biofilm eradication concentration (MBEC) values (in µg/mL) obtained for the tested compounds **2**–**5** are presented in Table 2. The quantitative testing of the antimicrobial activity showed that the majority of the tested compounds exhibited antimicrobial effects, with MIC values equal to or higher than 500 µg/mL. Among the analyzed compounds, **4b** was found to have a good antimicrobial activity (MIC value of 62.5 µg/mL) against two Gram-positive, i.e., *Bacillus subtilis* ATCC 6683*, Staphylococcus aureus* ATCC 6538, and one Gram-negative, i.e., *Escherichia coli* ATCC 8739 reference strains. Concerning the influence on the development of microbial biofilms on the inert substrate, the compounds **4a**, **4b**, **5a**, and **5b** did not interfere with the development of microbial biofilms on the inert substrate, at the tested range of concentrations. The compounds **2** and **3** exhibited antibiofilm effects in the case of the Gram-positive *E. faecium* E5 strain, with an MBEC value of 15.6 µg/mL. Comparing the MIC and MBEC values obtained for the newly synthesized derivatives with those obtained for ciprofloxacin and fluconazole, respectively, the tested compounds were shown to exhibit a weak antimicrobial activity.

### 2.3. Daphnia Magna Toxicity Assay

The *Daphnia magna* bioassay results are summarized in Table 3. Both at 24 and 48 h, compounds **2**, **4b**, **5b**, and positive controls induced lethality values lower than 35%, and therefore, the LC_50_ values couldn’t be determined. The LC_50_ was determined at 24 h only for compound **4a**, its value being greater than the maximum tested concentration as shown by the 95% CI range. After 48 h of exposure, compounds **3**, **4a**, and **5a** showed high to moderate cytotoxicity values, compound **3** being the most active of all. The correlation between concentrations and L% was higher than 0.7 for compounds **4a** and **5a**. The predicted values of **3**, **4a**, and **5a** are significantly lower than those obtained experimentally, and for all other compounds, the values are ranging from 0.18 to 68.2 µg/mL, despite no significant toxicity was recorded experimentally.

### 2.4. Prediction of the Molecular Mechanism of Action and Toxicity

#### 2.4.1. PASS Prediction

The software prediction of activity spectra for substances (PASS) is an application that can predict a large number of biological activities for a given molecule using its structure as input data. The software returns the probability of the compound to be active (Pa) or inactive (Pi) for each target [50]. The corresponding Pa values are presented in Table 4 for biological activities related to the antibacterial effects.

The Pa values are generally higher for compounds **4a** and **4b** than for the corresponding 1,3-oxazoles **5a** and **5b**, but this is an indication of the possibility that the new compounds produce an effect, and not for their potency. There are small differences between the not substituted derivatives (**a**) and the corresponding 4-methyl analogs (**b**).

The resulted Pa values indicate an acceptable potential for the new compounds to have a general antiinfective effect, and in the case of compound **3**, to have a similar effect with the glycopeptides antibiotics. The PASS prediction functions on the basis of the structural similarity between the tested structures and those included in the prediction set. The higher the value, the higher is the possibility to find an active drug. Nevertheless, a small probability could be an indicator for an original active structure.

#### 2.4.2. Structural Similarity Analysis

The similarity search on the ChEMBL database for compounds **2**, **3**, **4a,b**, and **5a,b** returned 62 analog compounds, with the highest degree of structural similarity (80.0%) being observed for the pair formed by compound CHEMBL2071499 and **4a**. The results highlight the originality of the newly synthesized compounds.

**Figure 4:**
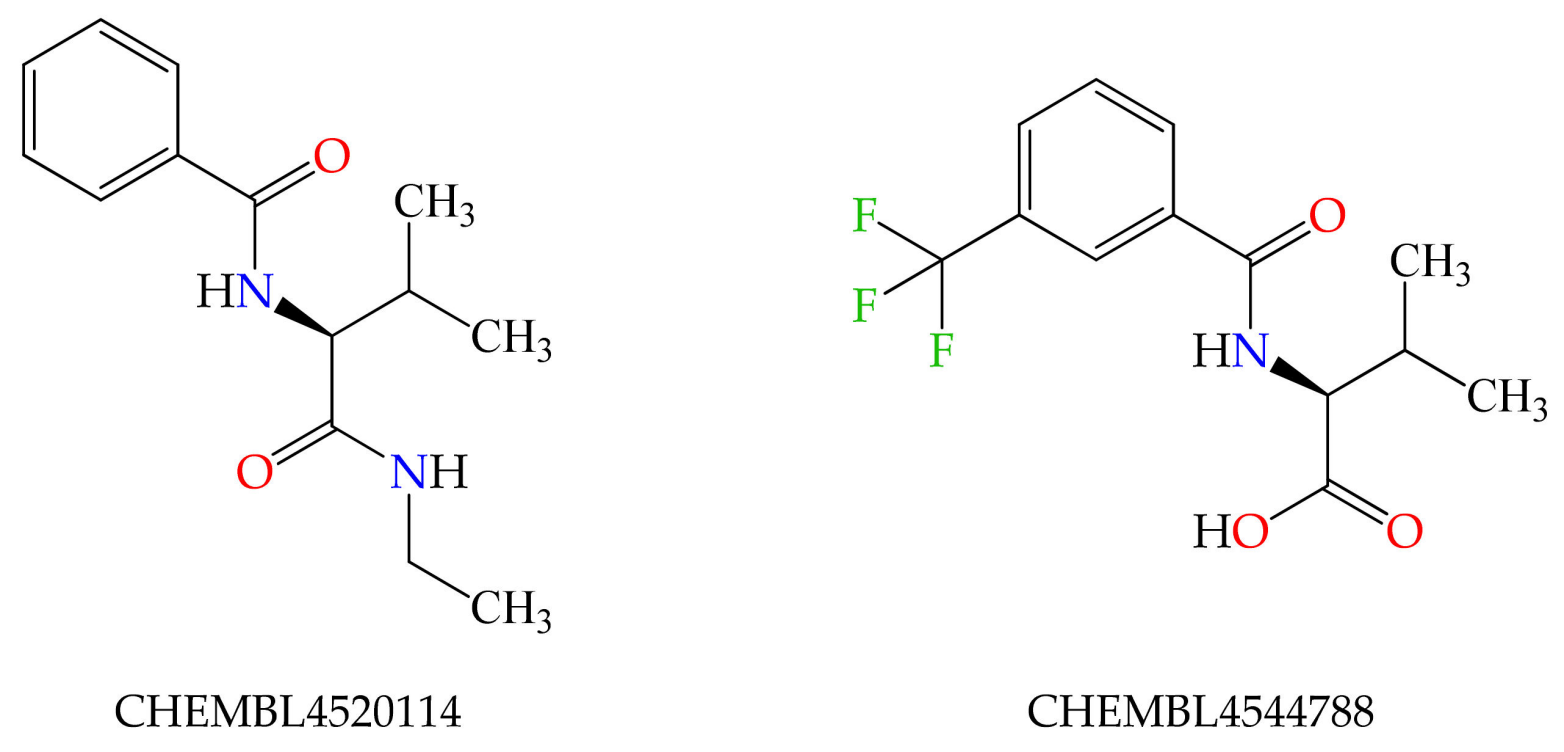
Structures of two compounds extracted from ChEMBL as structurally similar to compounds **4a** and **2**.

For only 2 of the 62 structurally similar compounds, there are antimicrobial results available, namely, for CHEMBL4520114 and CHEMBL4544788, both of which are *N*-benzoylvaline derivatives (Figure 4).

Compound CHEMBL4520114 shares a 51.1% structural similarity with **4a** and demonstrated low inhibition effects on several bacterial pathogens: *Acinetobacter baumannii* (7.1%), *Pseudomonas aeruginosa* ATCC 27,853 (7.1%), and *Pseudomonas aeruginosa* PAO397 (21.1%) after exposure to a 32 μg/mL concentration.

CHEMBL4544788 has a 52.2% structural similarity with **2** and low inhibition effects on *Staphylococcus aureus* (19.1%), *Acinetobacter baumannii* (4.3%), *Klebsiella pneumoniae* (3.7%), *Escherichia coli* (0.6%), and *Pseudomonas aeruginosa* (−9.2%) at 32 μg/mL.

## 3. Discussion

The 1,3-oxazole moiety gained attention in recent times due to its increasing importance in the field of medicinal chemistry. Being a doubly unsaturated 5-membered ring with one oxygen atom at position 1 and one nitrogen atom at position 3, the 1,3-oxazoles were identified as antibacterial, anticancer, and anti-inflammatory agents. The development of new antibacterial active substances is an ongoing process to improve the affinity for different bacterial strains. Some 1,3-oxazoles, e.g., 4-(1-benzofuran-2-yl)-1,3-oxazole-2-amine derivatives [51], showed appreciable antimicrobial activity as compared to the standard drugs, and a number of multi-substituted oxazoles containing a heterocyclic moiety exhibited pronounced antibacterial activity against *S. aureus*, *E. coli*, *B. subtilis*, and *K. pneumonia* [52].

In this paper, we report the synthesis of novel derivatives based on 1,3-oxazole and diphenyl sulfone scaffolds, starting from a natural α-amino acid, namely, from *L*-valine. The chemical structures of the novel compounds **2**–**5** were confirmed based on spectral studies. Furthermore, the newly synthesized compounds were evaluated for their antimicrobial and antibiofilm activities, for toxicity on *D. magna*, and by in silico studies regarding their potential mechanism of action and toxicity. Among the new tested compounds, the best antibacterial activity was revealed by compound **4b** (MIC value: 62.5 µg/mL) against *B. subtilis*, *S. aureus*, and *E. coli* reference strains. Additionally, compounds **2** and **3** were very active against the biofilm formed by *E. faecalis*. The *Daphnia magna* model is frequently used for determining the toxicity of drugs, biocompounds, plant extracts, as well as in ecotoxicological evaluations, and can be a useful tool for the anticancer and antibacterial activities prediction [53]. In the present study, the newly synthesized compounds were tested at selected concentrations, mainly chosen based on their solubility. The most toxic compound was compound **3**, followed by **4a** and **5a**. All other compounds showed low toxicity or no toxicity at all. The results indicate that these compounds have biological activity and are good candidates for further investigations. The high difference between the predicted and the experimentally obtained values of LC_50_ can be attributed to the low solubility in water of these compounds.

It can be noticed that the *N*-acyl-α-amino acid derivative **2** is promising due to its antibacterial and antibiofilm activities and low toxicity. Comparing the structure of this compound with that of the most probable pharmacological target: CHEMBL4544788, the biological action is probably due to the presence in the structure of the valine residue and also of the fragment derived from diphenyl sulfone. The antibacterial activity and antibiofilm effect against the Gram-positive *E. faecium* E5 strain are maintained by conversion of compound **2** to the corresponding 1,3-oxazol-5(4*H*)-one **3**, but the toxicity of the reaction product increases. Further, it appears that the transformation of *N*-acyl-α-amino acid **2** to *N*-acyl-α-amino ketone **4b** (via 1,3-oxazol-5(4*H*)-one **3**) does not increase the toxicity, but determines an improvement in the antimicrobial profile, with the disappearance of the antibiofilm activity. Compounds **4a**, **5a**, and **5b** were proved inactive, in the tested range of concentrations. Subsequent studies will reveal the structure-activity relationships (SAR). The different activity profiles for *N*-acyl-α-amino ketones **4** and 1,3-oxazoles derivatives **5** are probably due to the fact that acyclic intermediates **4** are much more reactive than the corresponding cyclodehydration products **5**. The higher antimicrobial potential of *N*-acyl-α-amino ketones **4** may be due to the presence in their structures of the two C=O double bonds and of the NH group which are both centers of reactivity and allow the formation of hydrogen bonds, being known that the intermolecular hydrogen-bonding capability is important to biological activity [54]. By cyclizing precursors **4**, to the corresponding heterocycles **5**, all these groups are no longer present in the structures of the molecules.

Further studies will be made for optimizations in order to obtain new derivatives with potent antimicrobial and antibiofilm activities. Thus, we identified three critical positions, which influence biological activity. A first possibility is to add various substituents on the diphenyl sulfone fragment, like nitro or fluoro, e.g., by using other aromatic compounds in the Friedel–Crafts sulfonylation. Desai et al. showed that the nitro group leads to a better efficacy against *S. pyogenes*, whereas the presence of the fluorine atom could increase the affinity for the active site of DNA gyrase [55]. The predictive studies indicated a high degree of similarity of **4a** and **2** with CHEMBL4520114 and CHEMBL4544788, respectively outlining a potential future synthesis approach. As a second choice of future improvement of these derivatives, other amino acids will be taken into account—cysteine, α-alanine, phenylalanine, and serine. As previously showed by Mhlongo et al., the naturally occurring oxazole-containing peptides, such as muscoride A and microcin B17, possessed strong antibacterial activity against *E. coli* strains by inhibition of DNA gyrase [13]. A third possibility is to use another aromatic compound in the Friedel–Crafts acylation reaction with 1,3-oxazol-5(4*H*)-one **3**. This step could also improve the antibacterial efficacy. Thus, in the future, we will consider the introduction of various substituents (e.g., a fluorine atom or a trifluoromethyl group) on the phenyl moiety at position 5 of the 1,3-oxazole nucleus for obtaining strong antimicrobial and antibiofilm analogs.

## 4. Materials and Methods

### 4.1.General Information

The melting points, m.p., were measured on a Boëtius apparatus (VEB Wägetechnik Rapido, PHMK 81/3026, Radebeul, Germany) and are uncorrected. The UV-Vis spectra were registered on a Specord 40 spectrophotometer (Analytik Jena AG, Jena, Germany) in a 1 cm pathlength quartz cuvette, for solutions in methanol (≈0.025 mM). The FT-IR spectra were acquired in KBr pellets on a Vertex 70 spectrometer (Bruker Optik GmbH, Ettlingen, Germany). Selected IR absorption bands are described as very strong, vs; strong, s; medium, m; weak; w. The NMR spectra were recorded on a Gemini 300 BB spectrometer (Varian, Inc., Palo Alto, CA, USA) in DMSO-d_6_ or CDCl_3_, at room temperature, operating at 300 MHz for ^1^H and 75 MHz for ^13^C. Combined 2D spectra (COSY, HETCOR) were also registered. Chemical shifts, *δ*, are in parts per million (ppm) relative to tetramethylsilane (TMS) used as internal standard and coupling constants, *J*, are expressed in hertz (Hz). The ^1^H-NMR signals multiplicity was abbreviated as follows: singlet, s; doublet, d; doublet of doublets, dd; triplet, t; triplet of triplets, tt; heptet, hp; heptet of doublets, hpd; octet, oct; multiplet, m; and a broad signal was abbreviated br. The ^1^H-NMR data are reported in the following order: chemical shift (multiplicity, coupling constants, number of protons, proton assignment), and the ^13^C-NMR data are quoted as follows: chemical shift (carbon attribution). Mass spectra of one representative from each class (**2**, **3**, **4a**, **5a**) were recorded on a Varian 1200L MS/MS triple quadrupole mass spectrometer (Varian, Inc., Walnut Creek, CA, USA) with an electrospray interface, in positive and/or negative ionization mode. A solution in methanol/water with 0.1% ammonium carbonate (or 0.1% ammonium formate and 1% formic acid) 9/1 (*v/v*) of around 1 ppm of these compounds was directly infused with a Prostar 240 SDM at 70 µL/min flow rate, using a Rheodyne manual injector. Pseudo-molecular ions (protonated molecules or deprotonated molecular ions) were selected with the first quadrupole. Fragments were obtained by collision with argon at different energies up to 50 eV. GC-EI-MS analysis was performed on a GC 8000 gas chromatograph, equipped with an electron impact quadrupole, and coupled to an MD 800 mass spectrometer detector (Fisons Instruments SpA, Rodano, Milano, Italy), using a fused-silica capillary column coated with poly(5% diphenyl/95% dimethylsiloxane) (SLB-5ms, 30 m × 0.32 mm, d_f_ 0.25 μm), a helium carrier gas flow rate of 2 mL/min, and dichloromethane as solvent. RP-HPLC chromatograms were acquired on a System Gold 126 liquid chromatograph (Beckman Coulter, Inc., Fullerton, CA, USA), with a System Gold 166 UV-Vis detector, a Rheodyne injection system, and a non-polar chromatography column (LiChrosorb RP-18, 25 cm × 4.6 mm, 5 μm particle size). The flow rate of the mobile phase (a mixture of methanol–water in various proportions) was 1 mL/min. Compounds’ purity (%) and retention time, *t*_R_, in minutes (min) were indicated. The elemental analysis was performed on an ECS 4010 elemental analyzer (Costech Analytical Technologies Inc., Valencia, CA, USA).

### 4.2. Chemistry

All the chemicals and reagents were purchased from commercial suppliers and used without purification. Dichloromethane was dried over anhydrous calcium chloride.

#### 4.2.1. Synthesis of 3-Methyl-2-[4-(phenylsulfonyl)benzamido]butanoic acid **2**

*L*-Valine (2.34 g, 20 mmol) was dissolved in 1 N sodium hydroxide solution (20 mL, 20 mmol). To the obtained solution, cooled in an ice bath to 0–5 °C, a solution of crude 4-(phenylsulfonyl)benzoyl chloride **1** (5.61 g, 20 mmol) in anhydrous dichloromethane (45 mL), and a 2 N NaOH solution (10 mL, 20 mmol), respectively were added simultaneously, dropwise, under magnetic stirring, for 30 min. The reaction mixture was then stirred for 1 h at room temperature. The aqueous phase was separated and acidified with 2 N hydrochloric acid. The formed precipitate was filtered off, washed with water, dried, and purified by recrystallization from water when white acicular crystals were obtained; yield = 89% (6.43 g); m.p. = 84–86 °C.

UV-Vis (CH_3_OH, *λ* nm) (lg *ε*): 202.6 (4.49); 241.0 (4.11).

FT-IR (KBr, *ν* cm^−1^): 3373s; 3088m; 3069s; 2967s; 2933s; 2875m; 2686m; 2609m; 1720vs; 1674vs; 1655vs; 1601m; 1570m; 1533vs; 1487m; 1465m; 1449s; 1311vs; 1296vs; 1156vs; 858m.

^1^H-NMR (DMSO-d_6_, *δ* ppm, *J* Hz): 0.89 (d, 6.9, 3H, H-19); 0.90 (d, 6.9, 3H, H-20); 2.12 (oct, 6.9, 1H, H-18); 4.26 (dd, 8.2, 6.6, 1H, H-4); 7.56 (dd, 7.8, 7.4, 2H, H-14, H-16); 7.62 (tt, 7.4, 1.5, 1H, H-15); 7.91 (dd, 7.8, 1.5, 2H, H-13, H-17); 7.96 (s, 4H, H-7, H-8, H-10, H-11); 8.38 (d, 8.0, 1H, H-3).

^13^C-NMR (DMSO-d_6_, *δ* ppm): 18.61 (C-19); 19.24 (C-20); 29.48 (C-18); 58.43 (C-4); 127.41 (C-13, C-17); 127.46 (C-8, C-10); 129.00 (C-7, C-11); 129.85 (C-14, C-16); 133.96 (C-15); 138.87 (C-6); 140.69 (C-12); 143.18 (C-9); 165.87 (C-2); 172.81 (C-5).

+ESI-MS/MS (*m*/*z*, rel. abund. %): 362 [M+H]^+^; 344 (14.9) [M+H-H_2_O]^+^; 316 (100, BP) [M+H-H_2_O-CO]^+^; 245 (15.4) [C_6_H_5_SO_2_C_6_H_4_CO]^+^.

−ESI-MS/MS (*m*/*z*, rel. abund. %): 360 (100, BP) [M-H]^-^; 316 (73.1) [M-H-CO_2_]^−^; 288 (10.1) [M-H-CO_2_-C_2_H_4_]^−^; 245 (5.2) [C_6_H_5_SO_2_C_6_H_4_CO]^−^; 217 (10.3) [C_6_H_5_SO_2_C_6_H_4_]^−^.

RP-HPLC (methanol–water 30:70, *v/v*; 1 mL/min; 250 nm): purity = 99.99%; *t*_R_ = 3.78 min.

Elemental analysis (%): Calculated for C_18_H_19_NO_5_S (361.41 g/mol): C, 59.82; H, 5.30; N, 3.88; S, 8.87. Found: C, 59.87; H, 5.28; N, 3.89; S, 8.85.

#### 4.2.2. Synthesis of 4-Isopropyl-2-[4-(phenylsulfonyl)phenyl]-1,3-oxazol-5(4*H*)-one **3**

3-Methyl-2-[4-(phenylsulfonyl)benzamido]butanoic acid **2** (3.79 g, 10.5 mmol) was suspended at room temperature, under magnetic stirring in 50 mL anhydrous dichloromethane and an equimolar quantity of 4-methylmorpholine (1.15 mL, 10.5 mmol) was added. Ethyl chloroformate (1 mL, 10.5 mmol) was then added slowly to the formed solution. The reaction mixture was further stirred at room temperature for another 30 min and then poured over 100 mL of the ice–water mixture. The organic phase was separated and washed with 5% sodium hydrogen carbonate solution, then with water and dried over anhydrous magnesium sulfate. After concentration by distillation under reduced pressure, the solid product was purified by recrystallization from cyclohexane as white crystals; yield = 90% (3.24 g); m.p. = 115–117 °C.

UV-Vis (CH_3_OH, *λ* nm) (lg *ε*): 202.6 (4.48); 247.6 (4.32).

FT-IR (KBr, *ν* cm^−1^): 3093w; 3066m; 2964s; 2931m; 2875m; 1827vs; 1649vs; 1599w; 1584w; 1570m; 1492w; 1466m; 1447s; 1326vs; 1309vs; 1295vs; 1244s; 1159vs; 1037vs; 843s.

^1^H-NMR (CDCl_3_, *δ* ppm, *J* Hz): 0.99 (d, 6.9, 3H, H-19); 1.14 (d, 6.9, 3H, H-20); 2.39 (hpd, 6.9, 4.7, 1H, H-18); 4.32 (d, 4.7, 1H, H-4); 7.54 (t, 7.8, 2H, H-14, H-16); 7.61 (tt, 7.5, 1.8, 1H, H-15); 7.90 (dd, 7.8, 1.8, 2H, H-13, H-17); 8.07 (d, 8.8, 2H, H-8, H-10); 8.15 (d, 8.8, 2H, H-7, H-11).

^13^C-NMR (CDCl_3_, *δ* ppm): 17.65 (C-19); 18.89 (C-20); 31.40 (C-18); 71.02 (C-4); 127.99 (C-13, C-17); 128.18 (C-8, C-10); 128.87 (C-7, C-11); 129.63 (C-14, C-16); 130.32 (C-6); 133.82 (C-15); 140.85 (C-12); 145.38 (C-9); 160.44 (C-2); 177.05 (C-5).

+ESI-MS/MS (*m*/*z*, rel. abund. %): 344 [M+H]^+^; 316 (100, BP) [M+H-CO]^+^; 245 (19.0) [C_6_H_5_SO_2_C_6_H_4_CO]^+^.

GC-EI-MS (*m*/*z*, rel. abund. %): 301 (100, BP) [M-C_3_H_6_]^+^; 245 (37.71) [C_6_H_5_SO_2_C_6_H_4_CHNH]^+^ or [C_6_H_5_SO_2_C_6_H_4_CO]^+^; 218 (17.37) [C_6_H_5_SO_2_C_6_H_5_]^+^; 152 (6.57); 132 (12.92); 125 (38.56) [C_6_H_5_SO]^+^; 104 (7.42) [C_6_H_4_CHNH]^+^; 97 (4.24) [C_3_H_7_CNCO]^+^; 77 (25.85) [C_6_H_5_]^+^; 76 (9.75) [C_6_H_4_]^+^; 51 (10.17) [C_4_H_3_]^+^; 44 (17.80) [C_3_H_8_]^+^ or [CO_2_]^+^; 43 (21.82) [C_3_H_7_]^+^; *t*_R_ = 26.25 min.

RP-HPLC (methanol–water 60:40, *v/v*; 1 mL/min; 250 nm): purity = 91.85%; *t*_R_ = 3.47 min.

Elemental analysis (%): Calculated for C_18_H_17_NO_4_S (343.40 g/mol): C, 62.96; H, 4.99; N, 4.08; S, 9.34. Found: C, 62.89; H, 4.95; N, 4.12; S, 9.28.

#### 4.2.3. General Procedure for the Synthesis of the *N*-(1-Aryl-3-methyl-1-oxobutan-2-yl)-4-(phenylsulfonyl)benzamides **4a,b**

An excess amount of anhydrous aluminum trichloride (2.00 g, 15 mmol) was added in portions, under stirring, at room temperature to crude 4-isopropyl-2-[4-(phenylsulfonyl)phenyl]-1,3-oxazol-5(4*H*)-one **3** (1.72 g, 5 mmol) in 25 mL of anhydrous aromatic hydrocarbon (benzene or toluene). Stirring was continued for 20 h until hydrogen chloride emission ceased, then the reaction mixture was poured over 100 mL of ice water acidified with 5 mL of 37% hydrochloric acid. The precipitate was filtered off, washed with cold water, then with a cold mixture of ethanol–water (1:1, *v/v*). The aqueous phase was extracted with 2 × 15 mL dichloromethane. Combined organic layers were washed with water, dried over anhydrous sodium sulfate, and concentrated by distillation under reduced pressure, leaving the second fraction of the raw product. Purification by recrystallization from ethanol leads to compound **4** as colorless crystals.

#### *N*-(3-Methyl-1-oxo-1-phenylbutan-2-yl)-4-(phenylsulfonyl)benzamide **4a**

Compound **4a** was obtained by reaction of **3** with benzene; yield = 80% (1.69 g); m.p. = 162–164 °C.

UV-Vis (CH_3_OH, *λ* nm) (lg *ε*): 202.6 (4.49); 246.7 (4.19).

FT-IR (KBr, *ν* cm^−1^): 3304m; 3088w; 3061m; 3039w; 2970m; 2935m; 2878m; 1662vs; 1595m; 1570m; 1528s; 1486m; 1468m; 1447m; 1324s; 1310m; 1300s; 1162s; 857m.

^1^H-NMR (DMSO-d_6_, *δ* ppm, *J* Hz): 0.90 (d, 7.4, 3H, H-19); 0.92 (d, 7.4, 3H, H-20); 2.23 (m, 1H, H-18); 5.38 (t, 7.7, 1H, H-4); 7.53 (t, 7.2, 2H, H-23, H-25); 7.63 (t, 7.7, 2H, H-14, H-16); 7.64 (m, 1H, H-24); 7.71 (tt, 7.7, 1.4, 1H, H-15); 7.99 (dd, 7.7, 1.4, 2H, H-13, H-17); 8.02 (d, 8.8, 2H, H-8, H-10); 8.05 (dd, 7.2, 1.6, 2H, H-22, H-26); 8.07 (d, 8.8, 2H, H-7, H-11); 8.99 (d, 8.0, 1H, H-3).

^13^C-NMR (DMSO-d_6_, *δ* ppm): 18.37 (C-19); 19.74 (C-20); 29.51 (C-18); 59.22 (C-4); 127.47 (C-8, C-10, C-22, C-26); 128.26 (C-13, C-17); 128.83 (C-23, C-25); 128.99 (C-7, C-11); 129.85 (C-14, C-16); 133.46 (C-24); 133.98 (C-15); 136.18 (C-21); 138.63 (C-6); 140.66 (C-12); 143.28 (C-9); 165.65 (C-2); 199.22 (C-5).

+ESI-MS/MS (*m*/*z*, rel. abund. %): 843 [2M+H]^+^; 422 [M+H]^+^; 245 (100, BP) [C_6_H_5_SO_2_C_6_H_4_CO]^+^; 161 (36.4) [C_6_H_5_COCHCH(CH_3_)_2_]^+^; 125 (65.8) [C_6_H_5_SO]^+^; 118 (15.7) [C_6_H_5_COCH]^+^; 105 (11.3) [C_6_H_5_CO]^+^.

−ESI-MS/MS (*m*/*z*, rel. abund. %): 841 [2M-H]^−^; 420 [M-H]^−^; 377 (100, BP) [M-H-C_3_H_7_]^−^; 300 (22.3) [M-H-C_3_H_7_-C_6_H_5_]^−^; 217 (36.0) [C_6_H_5_SO_2_C_6_H_4_]^−^; 141 (57.5) [C_6_H_5_SO_2_]^−^.

RP-HPLC (methanol–water 60:40, *v/v*; 1 mL/min; 250 nm): purity = 94.95%; *t*_R_ = 3.90 min.

Elemental analysis (%): Calculated for C_24_H_23_NO_4_S (421.51 g/mol): C, 68.39; H, 5.50; N, 3.32; S, 7.61. Found: C, 68.45; H, 5.48; N, 3.31; S, 7.64.

#### *N*-[3-Methyl-1-oxo-1-(*p*-tolyl)butan-2-yl]-4-(phenylsulfonyl)benzamide **4b**

Compound **4b** was obtained by reaction of **3** with toluene; yield = 74% (1.61 g); m.p. = 155–156 °C.

UV-Vis (CH_3_OH, *λ* nm) (lg *ε*): 202.6 (4.49); 252.0 (4.21).

FT-IR (KBr, *ν* cm^−1^): 3302s; 3091w; 3064w; 2965m; 2933w; 2875w; 1657vs; 1605s; 1570m; 1532s; 1484m; 1466m; 1447m; 1319s; 1308s; 1294s; 1158vs; 847m.

^1^H-NMR (DMSO-d_6_, *δ* ppm, *J* Hz): 0.89 (d, 6.9, 3H, H-19); 0.91 (d, 6.9, 3H, H-20); 2.26 (oct, 6.9, 1H, H-18); 2.36 (s, 3H, CH_3_); 5.35 (t, 7.7, 1H, H-4); 7.33 (d, 8.2, 2H, H-23, H-25); 7.63 (dd, 7.6, 7.2, 2H, H-14, H-16); 7.71 (tt, 7.2, 1.4, 1H, H-15); 7.96 (d, 8.2, 2H, H-22, H-26); 7.98 (dd, 7.6, 1.4, 2H, H-13, H-17); 8.02 (d, 8.8, 2H, H-8, H-10); 8.07 (d, 8.8, 2H, H-7, H-11); 8.96 (d, 8.0, 1H, H-3).

^13^C-NMR (DMSO-d_6_, *δ* ppm): 18.40 (C-19); 19.77 (C-20); 21.20 (CH_3_); 29.62 (C-18); 59.09 (C-4); 127.50 (C-8, C-10, C-13, C-17); 128.44 (C-22, C-26); 129.01 (C-7, C-11); 129.42 (C-23, C-25); 129.89 (C-14, C-16); 133.66 (C-21); 134.01 (C-15); 138.69 (C-6); 140.70 (C-12); 143.29 (C-24); 143.98 (C-9); 165.61 (C-2); 198.66 (C-5).

RP-HPLC (methanol–water 60:40, *v/v*; 1 mL/min; 250 nm): purity = 92.70%; *t*_R_ = 4.08 min.

Elemental analysis (%): Calculated for C_25_H_25_NO_4_S (435.54 g/mol): C, 68.94; H, 5.79; N, 3.22; S, 7.36. Found: C, 68.91; H, 5.73; N, 3.19; S, 7.33.

#### 4.2.4. General Procedure for the Synthesis of the 5-Aryl-4-isopropyl-2-[4-(phenylsulfonyl)phenyl]-1,3-oxazoles **5a,b**

Raw *N*-(1-aryl-3-methyl-1-oxobutan-2-yl)-4-(phenylsulfonyl)benzamide **4** (10 mmol) in phosphoryl trichloride (20 mL, 217.83 mmol) was refluxed for 4 h. Excess of POCl_3_ was distilled under vacuum. The oily residue was slowly and carefully poured into a crushed ice–water mixture and extracted twice with dichloromethane (20 mL). Combined organic phases were washed with 5% sodium hydrogen carbonate solution, then with water and dried over anhydrous sodium sulfate. After removal of the solvent by distillation under reduced pressure, crude solid **5** was purified by recrystallization from ethanol, as colorless crystals.

##### 4-Isopropyl-5-phenyl-2-[4-(phenylsulfonyl)phenyl]-1,3-oxazole **5a**

Compound **5a** was obtained from 4.22 g of **4a**; yield = 79% (3.19 g); m.p. = 168–169 °C.

UV-Vis (CH_3_OH, *λ* nm) (lg *ε*): 202.6 (4.48); 241.4 (4.06); 331.3 (4.23).

FT-IR (KBr, *ν* cm^−1^): 3085w; 3055m; 2966m; 2929m; 2871m; 1603m; 1589m; 1547w; 1495m; 1483m; 1465m; 1447s; 1323s; 1312s; 1294s; 1160vs; 1098s; 842m.

^1^H-NMR (CDCl_3_, *δ* ppm, *J* Hz): 1.36 (d, 6.9, 6H, H-19, H-20); 3.28 (hp, 6.9, 1H, H-18); 7.37 (tt, 7.7, 1.4, 1H, H-24); 7.47 (t, 7.7, 2H, H-23, H-25); 7.54 (brt, 7.7, 2H, H-14, H-16); 7.58 (tt, 7.7, 1.7, 1H, H-15); 7.64 (dd, 7.7, 1.4, 2H, H-22, H-26); 7.97 (dd, 7.7, 1.7, 2H, H-13, H-17); 8.02 (d, 8.8, 2H, H-8, H-10); 8.21 (d, 8.8, 2H, H-7, H-11).

^13^C-NMR (CDCl_3_, *δ* ppm): 22.07 (C-19, C-20); 26.12 (C-18); 126.26 (C-22, C-26); 127.01 (C-8, C-10); 127.78 (C-13, C-17); 128.28 (C-7, C-11); 128.36 (C-24); 128.90 (C-21); 129.01 (C-23, C-25); 129.49 (C-14, C-16); 132.11 (C-6); 133.46 (C-15); 141.54 (C-9); 142.29 (C-12); 144.15 (C-4); 145.41 (C-5); 157.93 (C-2).

+ESI-MS/MS (*m*/*z*, rel. abund. %): 404 [M+H]^+^; 388 (13.1) [M+H-CH_4_]^+^; 262 (42.0) [C_6_H_5_SO_2_C_6_H_4_CONH_3_]^+^; 248 (30.6) [M+H-CH_4_-C_6_H_5_SO_2_+H]^+^; 245 (38.1) [C_6_H_5_SO_2_C_6_H_4_CO]^+^; 125 (100, BP) [C_6_H_5_SO]^+^.

RP-HPLC (methanol–water 70:30, *v/v*; 1 mL/min; 335 nm): purity = 99.99%; *t*_R_ = 5.08 min.

Elemental analysis (%): Calculated for C_24_H_21_NO_3_S (403.49 g/mol): C, 71.44; H, 5.25; N, 3.47; S, 7.95. Found: C, 71.39; H, 5.23; N, 3.48; S, 7.93.

##### 4-Isopropyl-2-[4-(phenylsulfonyl)phenyl]-5-(*p*-tolyl)-1,3-oxazole **5b**

Compound **5b** was obtained from 4.36 g of **4b**; yield = 76% (3.17 g); m.p. = 206–207 °C.

UV-Vis (CH_3_OH, *λ* nm) (lg *ε*): 202.6 (4.47); 243.2 (4.07); 336.6 (4.17).

FT-IR (KBr, *ν* cm^−1^): 3055m; 3030m; 2970m; 2932m; 2872m; 1597s; 1547m; 1508s; 1481m; 1464m; 1450s; 1322vs; 1292s; 1157vs; 1095vs; 842m.

^1^H-NMR (CDCl_3_, *δ* ppm, *J* Hz): 1.35 (d, 6.6, 6H, H-19, H-20); 2.40 (s, 3H, CH_3_); 3.26 (hp, 6.9, 1H, H-18); 7.27 (d, 8.2, 2H, H-23, H-25); 7.52 (brt, 7.3, 2H, H-14, H-16); 7.53 (d, 8.2, 2H, H-22, H-26); 7.58 (tt, 7.3, 1.7, 1H, H-15); 7.96 (dd, 7.3, 1.7, 2H, H-13, H-17); 8.02 (d, 8.5, 2H, H-8, H-10); 8.20 (d, 8.5, 2H, H-7, H-11).

^13^C-NMR (CDCl_3_, *δ* ppm): 21.46 (CH_3_); 22.07 (C-19, C-20); 26.08 (C-18); 126.20 (C-22, C-26); 126.93 (C-8, C-10); 127.76 (C-13, C-17); 128.25 (C-7, C-11); 129.48 (C-14, C-16); 129.69 (C-23, C-25); 132.17 (C-6); 133.45 (C-15); 138.43 (C-21); 141.54 (C-9); 142.11 (C-12); 143.56 (C-4, C-24); 145.60 (C-5); 157.65 (C-2).

RP-HPLC (methanol–water 70:30, *v*/*v*; 1 mL/min; 335 nm): purity = 99.99%; *t*_R_ = 5.70 min.

Elemental analysis (%): Calculated for C_25_H_23_NO_3_S (417.52 g/mol): C, 71.92; H, 5.55; N, 3.35; S, 7.68. Found: C, 71.98; H, 5.53; N, 3.36; S, 7.68.

### 4.3. Antimicrobial Activity Assessment

The antimicrobial activity of the compounds was investigated using the agar disc-diffusion method, broth microdilution, and microtiter plate assay.

#### 4.3.1. Microbial Strains

Microbial strains used in this study included three Gram-positive bacteria: *Bacillus subtilis* 6683, *Staphylococcus aureus* ATCC 6538, *Enterococcus faecium* E5, two Gram-negative bacteria: *Escherichia coli* ATCC 8739, *Pseudomonas aeruginosa* ATCC 27853, and a fungal strain: *Candida albicans* 393.

#### 4.3.2. Qualitative Assessment of the Antimicrobial Activity

The qualitative assessment of the antimicrobial activity of the tested compounds was performed using the agar disc-diffusion method. The Mueller Hinton (MH) agar plates are inoculated with a standardized microbial inoculum prepared in phosphate-buffered saline and adjusted to 0.5 McFarland scale. Then, five microliters of the solution of the tested compound (of 5000 µg/mL concentration in DMSO) were pipetted on the inoculated agar surface. After incubation of the plates for 24 h at 37 °C, the diameters of inhibition growth zones were measured.

#### 4.3.3. Investigation of the Influence of the Tested Compounds on the Antibiotic Susceptibility Spectrum of the Studied Strain

Subinhibitory concentrations (250 µg/mL) of tested compounds **2** and **3** were achieved in sterile liquid culture medium; the obtained tubes were inoculated with 0.5 McFarland suspensions obtained from the 24 h microbial culture of *E. faecium* E5. A control of DMSO (bacterial strain grown in the presence of DMSO) and control of microbial growth (culture medium inoculated with microbial suspension) were also prepared. The inoculated tubes were incubated at 37 °C for 24 h. The microbial cultures obtained in the presence of subinhibitory concentrations of tested compounds **2** and **3**, DMSO and control cultures, respectively, were used to determine the influence of the tested compounds on the antibiotic susceptibility spectrum of the studied strain. Thus, the liquid microbial cultures were sedimented by centrifugation at 10,000× *g* for 5 min, and the obtained cellular sediment was washed 3 times in sterile saline by centrifugation at 10,000× *g* for 5 min. The cell pellet was resuspended in sterile saline until a turbidity corresponding to the 0.5 McFarland standard was obtained and the classical Kirby–Bauer method was then performed to assess the susceptibility to the following antibiotics (bioMérieux, France): ampicillin, penicillin, linezolid, vancomycin for the *E. faecium* E5 strain. The results were recorded after 24 h of incubation at 37 °C. The diameters of the areas of inhibition of bacterial growth were interpreted according to the recommendations of the current edition of the *Clinical and Laboratory Standards Institute* (CLSI).

#### 4.3.4. Quantitative Assessment of the Antimicrobial Activity

Quantitative assessment of the antimicrobial activity of the tested compounds was performed using broth microdilution, in 96-well microtiter plates. The tested concentrations of the solutions of the different compounds in DMSO achieved through double serial dilutions, in columns 1–10, were between 500–0.97 µg/mL. Then, the wells were inoculated with 10 µL microbial suspension prepared in the same medium after dilution (1:100) of standardized microbial inoculum adjusted to 0.5 McFarland scale. Column 11 contained 10 µL of standardized inoculum and 90 μL of Mueller Hinton Broth, and column 12 contained 100 µL of Mueller Hinton Broth (as a control to monitor sterility). Ciprofloxacin (Sigma–Aldrich, St. Louis, MO, USA) and fluconazole (Sigma–Aldrich) served as positive controls. The microtiter plates were incubated without agitation for 24 h at 37 °C. In order to confirm the MIC value, the assays were performed in triplicate. The MIC was determined as the lowest concentration of tested compound that inhibited the growth of the microorganism as detected spectrophotometrically at 620 nm with an Apollo LB 911 ELISA Reader (Berthold Technologies GmbH & Co. KG, Waltham, MA, USA) [56].

#### 4.3.5. Evaluation of the Antibiofilm Activity

Evaluation of the antibiofilm activity of tested compounds was carried out using the microtiter biofilm inhibition assay. Briefly, in a 96-well polystyrene microtiter plate containing 90 μL of Mueller Hinton Broth, 90 μL of the tested compound solution was added in column 1. Serial two-fold dilutions were performed in columns 1–10. A volume of 10 μL microbial suspension (final OD600 = 0.01), prepared from an overnight culture grown in TSB into MH broth, was added. Column 11 contained 10 µL of standardized inoculum and 90 μL of Mueller Hinton Broth, and column 12 contained 100 µL of Mueller Hinton Broth (as a control to monitor sterility). Ciprofloxacin (Sigma–Aldrich) and fluconazole (Sigma–Aldrich) served as positive controls. Microplates were incubated for 24 h at 37 °C under static conditions to allow for microbial growth and biofilm maturation. The wells of the microplate were emptied and washed twice with phosphate-buffered saline. The biofilms formed on the walls of wells of the microplate were fixed with methanol for 5 min and stained with 1% crystal violet solution for 15 min, then rinsed three times with distilled water to remove the unbound dye. The fixed dye was resuspended in 33% acetic acid and the A492 was recorded with an Apollo LB 911 ELISA Reader. The amount of biofilm inhibition was calculated relative to the amount of biofilm that was grown in the absence of the tested compound and the media sterility control. The minimal biofilm eradication concentration (MBEC) was determined to be the lowest concentration of the tested compounds at which the decrease in absorbance value, measured at 492 nm, was observed in comparison to the positive control. Results from at least three separate biological replicates were averaged [57].

### 4.4. Daphnia Magna Toxicity Assay

*D. magna* Straus was maintained parthenogenetically (‘Carol Davila’ University—Department of Pharmaceutical Botany and Cell Biology) at 25 °C with a photoperiod of 16 h/8 h light/dark cycle in a Sanyo MLR-351 H climatic chamber (Sanyo, San Diego, CA, USA). With 24 h prior to the determination, young daphnids were selected according to their size and maintained for 24 h in an artificial medium. Ten daphnids/replicate were used, and the determination was performed in tissue culture plates with 12 wells (Greiner Bio-One) [58,59]. Each compound was tested in six concentrations, ranging from 2.2 to 44 µg/mL. *L*-Valine—control 1 (2.5–50 µg/mL) and 4-(phenylsulfonyl)benzoic acid—control 2 (1.1–22 µg/mL) were used as positive controls, and a 1% DMSO solution as a negative control. The concentration ranges were selected based on the solubility and a pre-screening assay. All determinations were performed in duplicate. The lethality was evaluated after 24 and 48 h of exposure. LC_50_ and 95% confidence intervals (95% CI) were calculated using the least square fit method. All calculations were performed using GraphPad Prism v 5.1 software (GraphPad Software, Inc., La Jolla, CA, USA). Freely available online GUSAR software (Institute of Biomedical Chemistry, Moscow, Russia) was used to predict the LC_50_ values for 48 h exposure of the new compounds [60].

### 4.5. Prediction of the Molecular Mechanism of Action and Toxicity

#### 4.5.1. PASS Prediction

A virtual screening was performed using the software PASS (Prediction of Activity Spectra for Substances), an application designed to evaluate the pharmacological potential of newly synthesized compounds. The structures were inputted in PASS as SMILES and the results were analyzed if the Pa values were above the corresponding Pi values.

#### 4.5.2. Structural Similarity Analysis

A similarity search was performed on the ChEMBL database for the newly synthesized compounds using a 50% threshold [58]. The resulting structures were extracted together with their assayed activities on bacteria [61]. The entries were filtered using DataWarrior v5.2.1 software [62] to remove duplicate structures.

## 5. Conclusions

In this paper, new derivatives from *N*-acyl-α-amino acids, 1,3-oxazol-5(4*H*)-ones, *N*-acyl-α-amino ketones, and 1,3-oxazoles classes, that incorporate into the structure a 4-(phenylsulfonyl)phenyl fragment, were synthesized, and physicochemically characterized. *N*-Acyl-α-amino acid **2** was produced by Steiger acylation of *L*-valine with acyl chloride **1**. Saturated 2-aryl-4-isopropyl-1,3-oxazol-5(4*H*)-one **3** was prepared from open-chain intermediate **2** by intramolecular cyclodehydration. *N*-Acyl-α-amino ketones **4a,b** were synthesized by treatment of 2,4-disubstituted 1,3-oxazol-5(4*H*)-one **3** with arenes, in presence of aluminum trichloride. The 2,5-diaryl-4-isopropyl-1,3-oxazoles **5a,b** were generated from acyclic precursors **4a,b** under the action of phosphoryl trichloride. The structures of the newly synthesized compounds were confirmed through spectral and elemental analysis data. The antimicrobial activity evaluation demonstrated that compound **4b** exhibited inhibitory effects against the planktonic growth of both Gram-positive and Gram-negative strains, while compounds **2** and **3** have inhibited the *E. faecium* biofilm development on the inert substrate. Despite the low antimicrobial effect of the newly synthesized compounds, the general scaffold allows several future optimizations for obtaining better novel antimicrobial agents.

## 6. Patents

Patent application a201900668: Theodora-Venera Apostol, Stefania-Felicia Barbuceanu, Laura-Ileana Socea, Ioana Saramet, Constantin Draghici, Valeria Radulescu, Mariana Carmen Chifiriuc, Luminita Gabriela Marutescu, Octavian Tudorel Olaru, George Mihai Nitulescu, 4-Isopropyl-1,3-oxazol-5(4*H*)-one Derivatives Containing a Diaryl sulfonyl Substituent in Position 2 with Antimicrobial Action, published in RO-BOPI, 8/2020 from 28 August 2020.

## Figures and Tables

**Table 1:** Antibiotic susceptibility testing results obtained for *E. faecium* E5 cultivated in the presence of sub-inhibitory concentrations of compounds **2** and **3** and of DMSO.

Bacteria Tested	Diameter (mm) of the Zone of Inhibition of Growth			
	Ampicillin (S ^1^, ≥17 mm)	Penicillin (S, ≥15 mm)	Linezolid (S, ≥23 mm)	Vancomycin (S, ≥17 mm)
*E. faecium* E5	24	14	27	19
*E. faecium* E5 treated with compound 2	24	13	28	19
*E. faecium* E5 treated with compound 3	23	12	27	18
*E. faecium* E5 treated with DMSO	22	0	23	18

^1^ S = susceptible, according to the current edition of the *Clinical and Laboratory Standards Institute* (CLSI).

**Table 2:** The MIC and MBEC values (µg/mL) obtained for the tested compounds.

**Compound**	*Enterococcus faecium* E5		*Staphylococcus aureus* ATCC 6538		*Bacillus subtilis* ATCC 6683		*Pseudomonas aeruginosa* ATCC 27857		*Escherichia coli* ATCC 8739		*Candida albicans* 393	
	**MIC**	**MBEC**	**MIC**	**MBEC**	**MIC**	**MBEC**	**MIC**	**MBEC**	**MIC**	**MBEC**	**MIC**	**MBEC**
**2**	500	15.6	>500	>500	>500	>500	>500	>500	>500	>500	>500	>500
**3**	500	15.6	>500	>500	>500	>500	>500	>500	>500	>500	>500	>500
**4a**	>500	>500	>500	>500	>500	>500	>500	>500	>500	>500	>500	>500
**4b**	>500	>500	62.5	>500	62.5	>500	>500	>500	62.5	>500	>500	>500
**5a**	>500	>500	>500	>500	>500	>500	>500	>500	>500	>500	>500	>500
**5b**	>500	>500	>500	>500	>500	>500	>500	>500	>500	>500	>500	>500
Ciprofloxacin	0.62	0.62	0.15	0.15	<0.03	<0.03	0.15	0.15	0.012	0.012	- ^1^	-
Fluconazole	-	-	-	-	-	-	-	-	-	-	<0.12	<0.12

^1^ -, not tested.

**Table 3:** *Daphnia magna* bioassay: 24 h and 48 h values, 95% confidence intervals for newly synthesized compounds and their starting materials.

Tested Compound	Predicted LC_50 (48 h)_ ^1^ (µg/mL)	Max. L_(48 h)_% ^2^	Determined LC_50 (24 h)_ (µg/mL)	95% CI ^3^ of LC_50__(24 h)_ (µg/mL)	Determined LC_50 (48 h)_ (µg/mL)	95% CI of LC_50__(48 h)_ (µg/mL)
**2**	5.31	10	ND ^4^ *	ND *	ND *	ND *
**3**	1.92	10	ND *	ND *	21.73	ND **
**4a**	1.26	60	89.63	33.88 to 237.1	27.32	18.64 to 40.05
**4b**	0.41	30	ND *	ND *	ND *	ND *
**5a**	0.37	35	ND *	ND *	77.02	47.85 to 124.0
**5b**	0.18	15	ND *	ND *	ND *	ND *
*L*-Valine (control 1)	1078.3	15	ND *	ND *	ND *	ND *
4-(Phenylsulfonyl)benzoic acid (control 2)	68.2	20	ND *	ND *	ND *	ND *

^1^ LC_50_—50% lethal concentration; ^2^ Max. L_(48 h)_%—maximum lethality induced at 48 h; ^3^ 95% CI–95% confidence interval; ^4^ ND—not determined due to lethality values; * the compound induced lethality values between 0 and 35%; ** 95% CI is too wide to be determined.

**Table 4:** The probability of compounds **2**, **3**, **4a,b**, and **5a,b** to be active (Pa) as antimicrobials as predicted by PASS analysis.

Target	2	3	4a	4b	5a	5b
Antibiotic glycopeptide-like	0.1	0.56	-	-	-	-
Antimycobacterial	0.40	0.49	0.42	0.43	0.28	0.29
Antituberculosis	0.43	0.36	0.43	0.43	0.25	0.25
Antiinfective	0.50	0.32	0.32	0.36	0.26	0.29

## Data Availability

The datasets used and/or analyzed during the current study are available from the corresponding author on reasonable request.

## Supplementary Materials

The following are available.

## Sample Availability

Samples of the compounds are not available from the authors.
