# Supplementary material for: Synthesis and Biological Evaluation of New N-Acyl-α-amino Ketones and 1,3-Oxazoles Derivatives
Source: Molecules. 2021 Aug 19;26(16):5019. doi: 10.3390/molecules26165019 (PMC8400786; doi:10.3390/molecules26165019)
Supplement: Supplementary file 1 [file molecules-26-05019-s001.zip › molecules-1330479-supplementary.pdf]

molecules

MDPI

Supplementary Material

Synthesis and Biological Evaluation of New *N*-Acyl-α-amino Ketones and 1,3-Oxazoles Derivatives

Theodora-Venera Apostol [1], Luminita Gabriela Marutescu [2,*], Constantin Draghici [3], Laura-Ileana Socea [1], Octavian Tudorel Olaru [1,*], George Mihai Nitulescu [1], Elena Mihaela Pahontu [1], Gabriel Saramet [1], Cristian Enache-Preoteasa [4] and Stefania-Felicia Barbuceanu [1]

[1] Faculty of Pharmacy, "Carol Davila" University of Medicine and Pharmacy, 6 Traian Vuia Street, 020956 Bucharest, Romania; theodora.apostol@umfcd.ro (T.-V.A.); laura.socea@umfcd.ro (L.-I.S.); george.nitulescu@umfcd.ro (G.M.N.); elena.pahontu@umfcd.ro (E.M.P.); gabriel.saramet@umfcd.ro (G.S.); stefania.barbuceanu@umfcd.ro (S.-F.B.)

[2] Department of Botany and Microbiology, Faculty of Biology & Research Institute of the University of Bucharest (ICUB), University of Bucharest, 060101 Bucharest, Romania

[3] "Costin D. Nenițescu" Centre of Organic Chemistry, Romanian Academy, 202 B Splaiul Independenței, 060023 Bucharest, Romania; cst_drag@yahoo.com

[4] National Phytosanitary Laboratory, 11 Voluntari Boulevard, 077190 Voluntari, Ilfov, Romania; cristian.enache@anfdf.ro

* Correspondence: octavian.olaru@umfcd.ro (O.T.O.), luminita.marutescu@bio.unibuc.ro (L.G.M.);

Abstract: In order to develop novel bioactive substances with potent activities, some new valine-derived compounds incorporating a 4-(phenylsulfonyl)phenyl fragment, namely acyclic precursors from *N*-acyl-α-amino acids and *N*-acyl-α-amino ketones classes, and heterocycles from the large family of 1,3-oxazole-based compounds, were synthesized. The structures of the new compounds were established using elemental analysis and spectral (UV-Vis, FT-IR, MS, NMR) data, and their purity was checked by reversed-phase HPLC. The newly synthesized compounds were evaluated for their antimicrobial and antibiofilm activities, for toxicity on *D. magna*, and by *in silico* studies regarding their potential mechanism of action and toxicity. The 2-aza-3-isopropyl-1-[4-(phenylsulfonyl)phenyl]-1,4-butanedione **4b**, bearing a *p*-tolyl group in 4-position exhibited the best antibacterial activity against the planktonic growth of both Gram-positive and Gram-negative strains, while the *N*-acyl-α-amino acid **2** and 1,3-oxazol-5(4*H*)-one **3** inhibited the *Enterococcus faecium* biofilms. Despite not all newly synthesized compounds showed significant biological activity, the general scaffold allows several future optimizations for obtaining better novel antimicrobial agents by the introduction of various substituents on the phenyl moiety at position 5 of the 1,3-oxazole ring.

Keywords: *N*-acyl-α-amino acid; 4-isopropyl-1,3-oxazol-5(4*H*)-one; *N*-acyl-α-amino ketone; 4-isopropyl-1,3-oxazole; antimicrobial activity; antibiofilm agents; toxicity

Supplementary Material

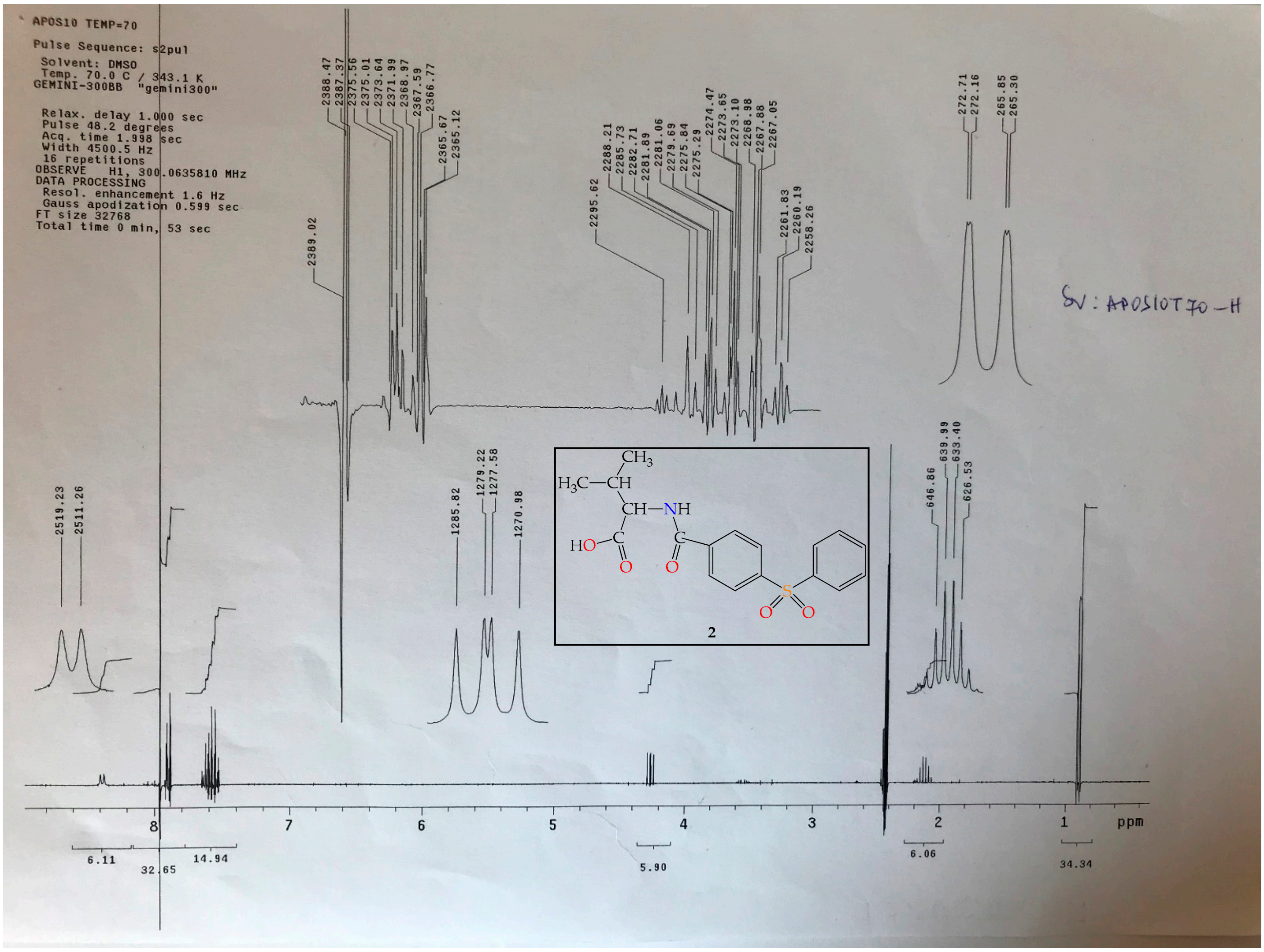

Figure S1. The ^{1}H-NMR spectrum of 3-methyl-2-[4-(phenylsulfonyl)benzamido]butanoic acid **2**.

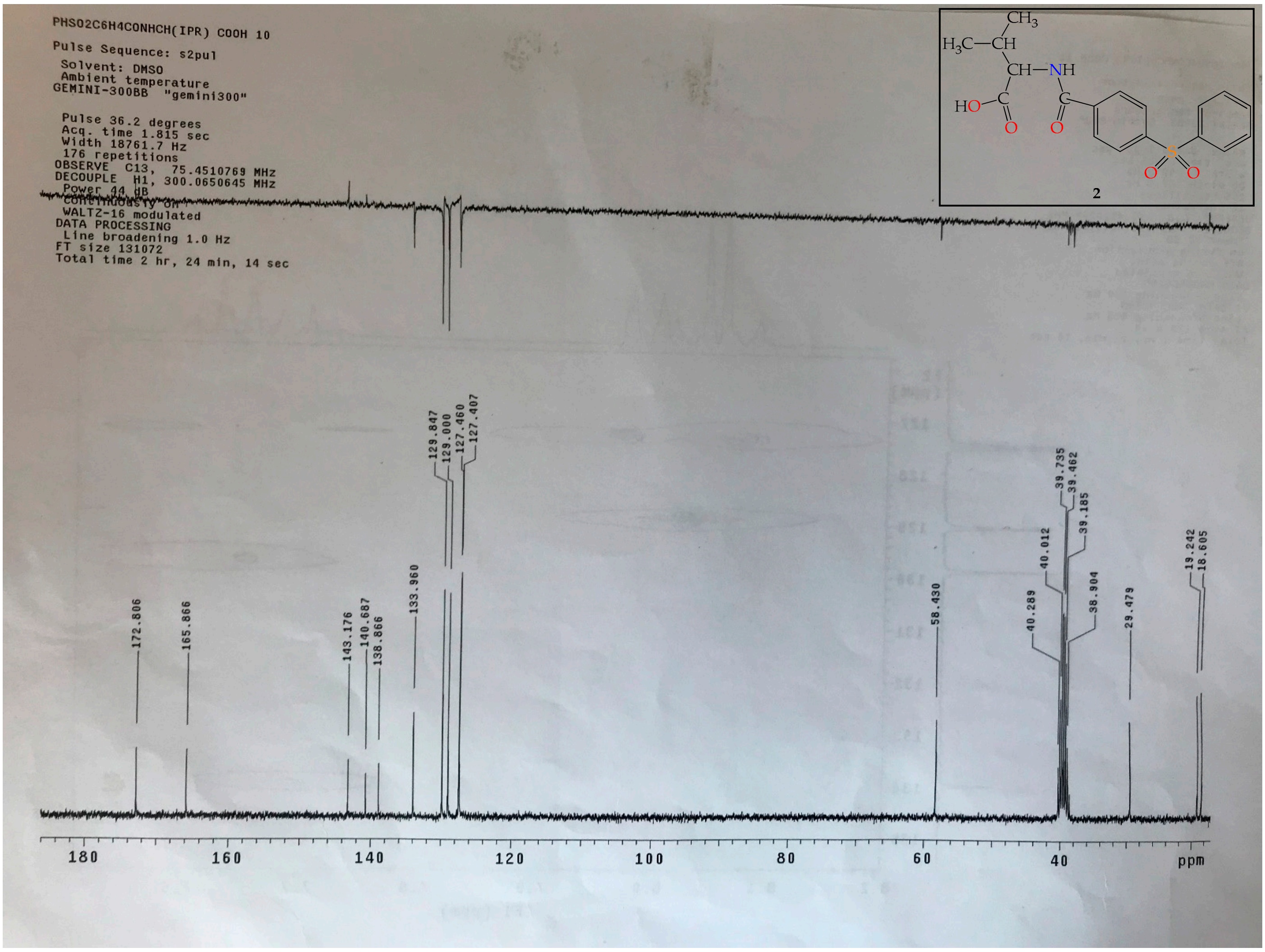

Figure S2. The ^{13}C-NMR spectrum of 3-methyl-2-[4-(phenylsulfonyl)benzamido]butanoic acid **2**.

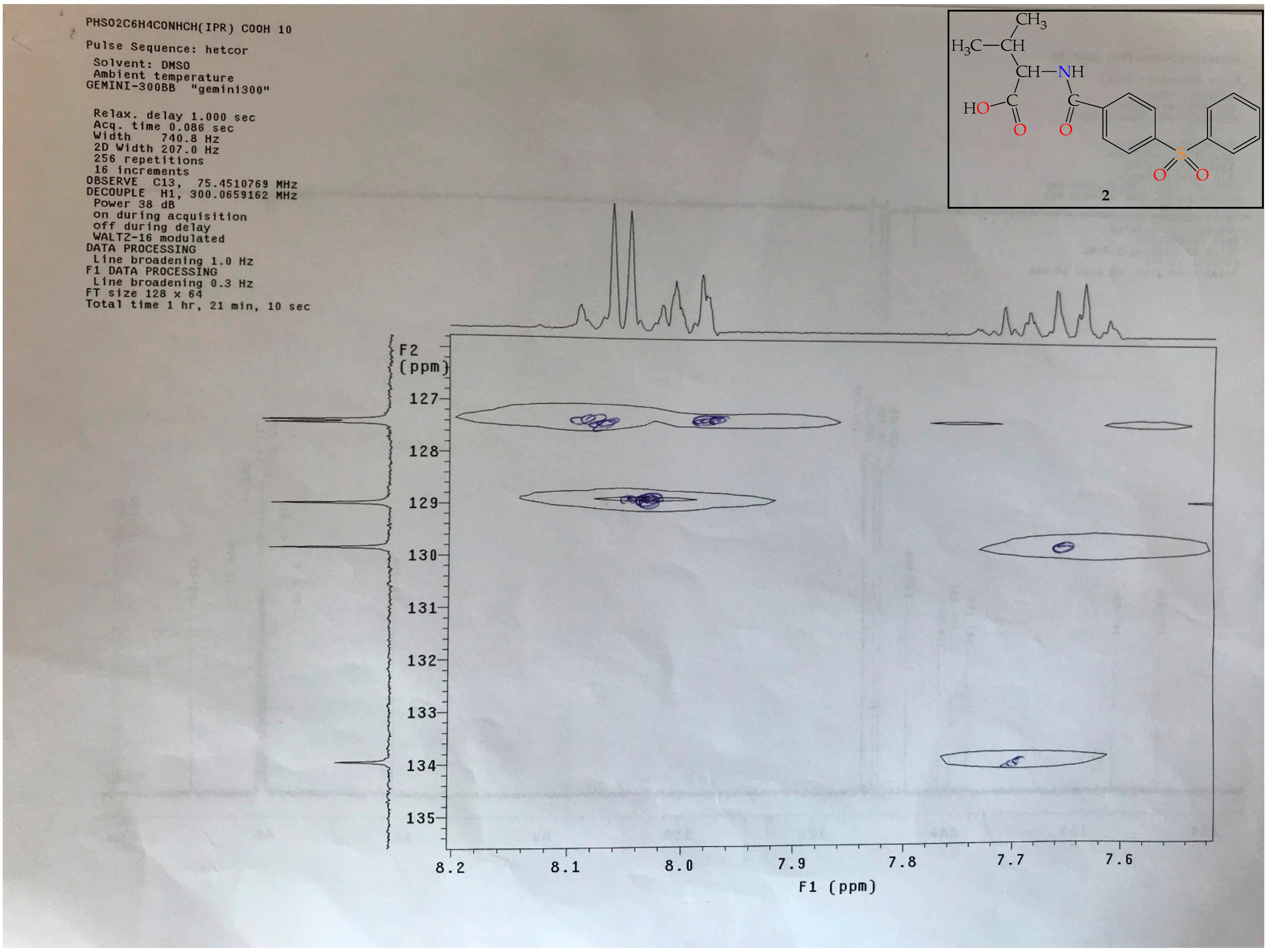

Figure S3. The 2D HETCOR spectrum of 3-methyl-2-[4-(phenylsulfonyl)benzamido]butanoic acid **2**.

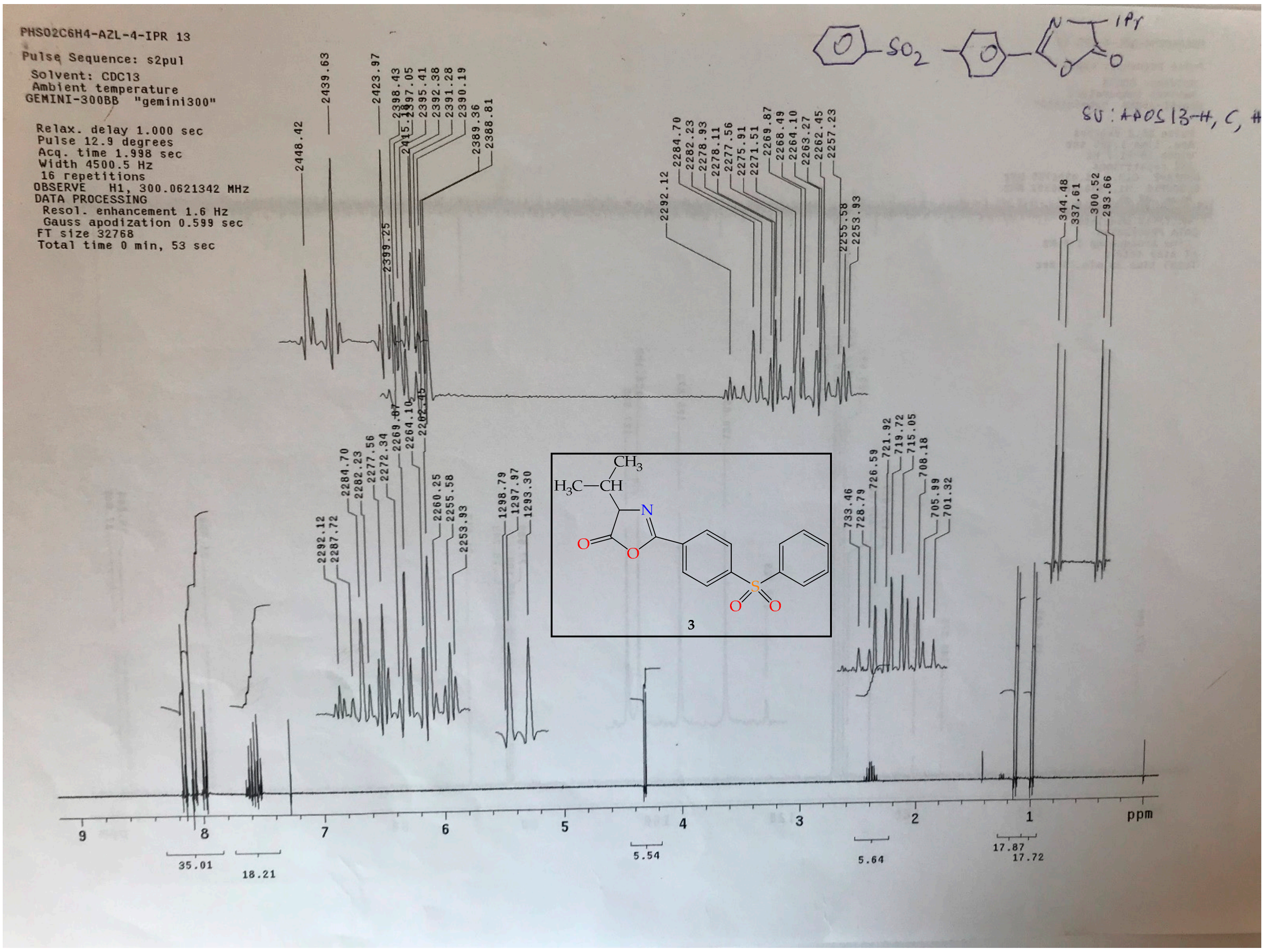

Figure S4. The ^{1}H-NMR spectrum of 4-isopropyl-2-[4-(phenylsulfonyl)phenyl]-1,3-oxazol-5(4*H*)-one **3**.

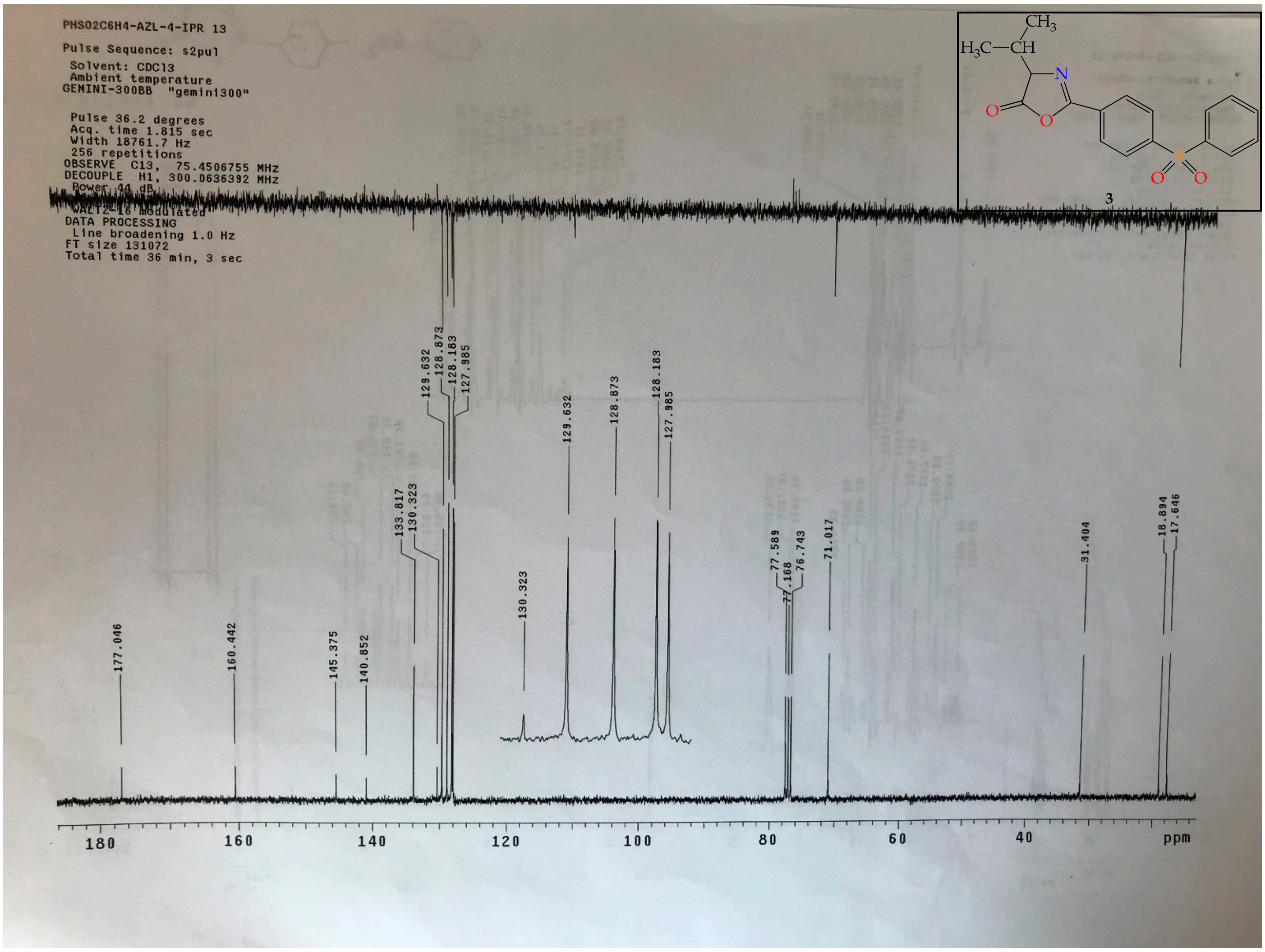

Figure S5. The ^{13}C-NMR spectrum of 4-isopropyl-2-[4-(phenylsulfonyl)phenyl]-1,3-oxazol-5(4*H*)-one **3**.

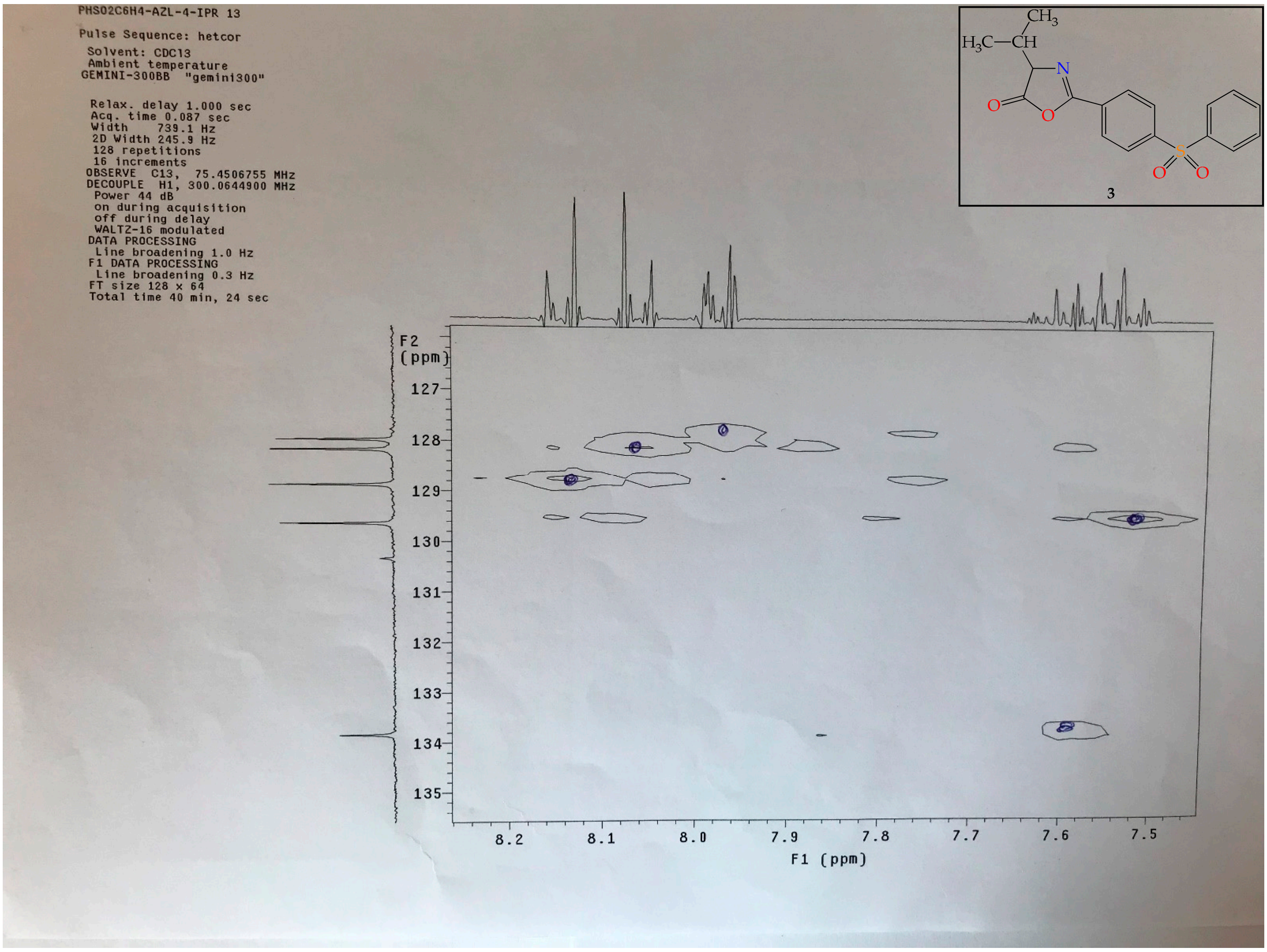

Figure S6. The 2D HETCOR spectrum of 4-isopropyl-2-[4-(phenylsulfonyl)phenyl]-1,3-oxazol-5(4*H*)-one **3**.

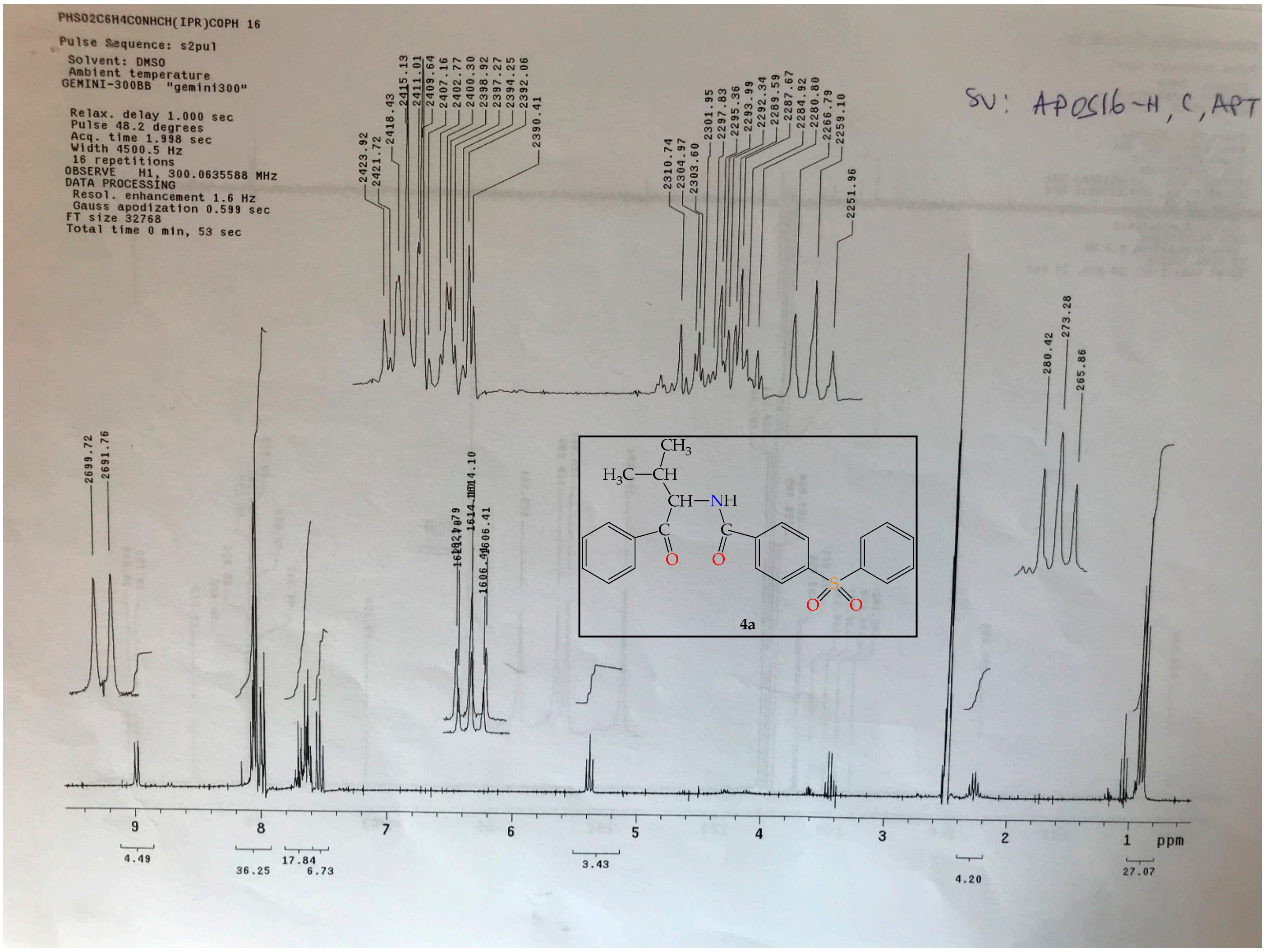

Figure S7. The ^{1}H-NMR spectrum of *N*-(3-methyl-1-oxo-1-phenylbutan-2-yl)-4-(phenylsulfonyl)benzamide **4a**.

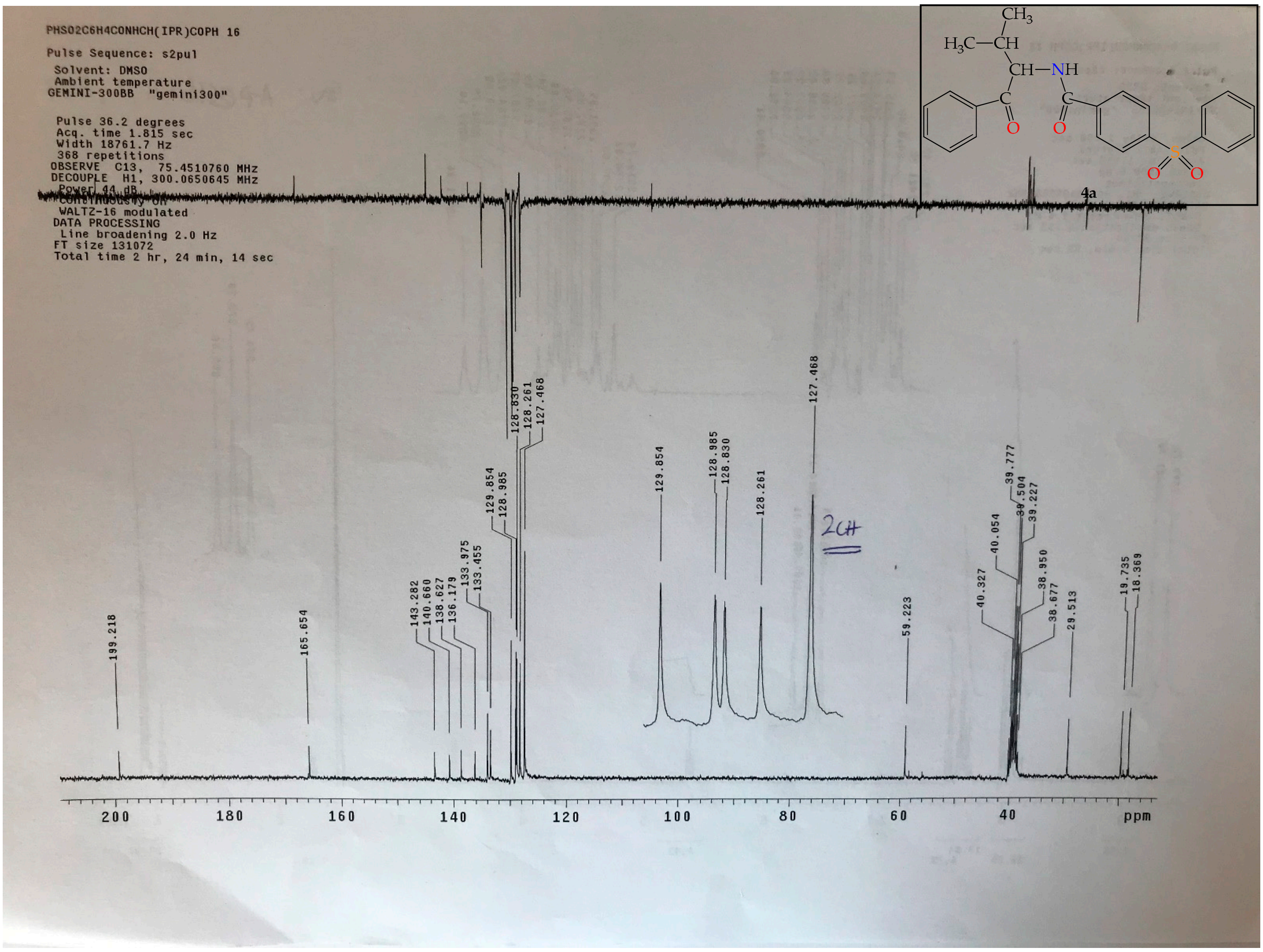

Figure S8. The ^{13}C-NMR spectrum of *N*-(3-methyl-1-oxo-1-phenylbutan-2-yl)-4-(phenylsulfonyl)benzamide **4a**.

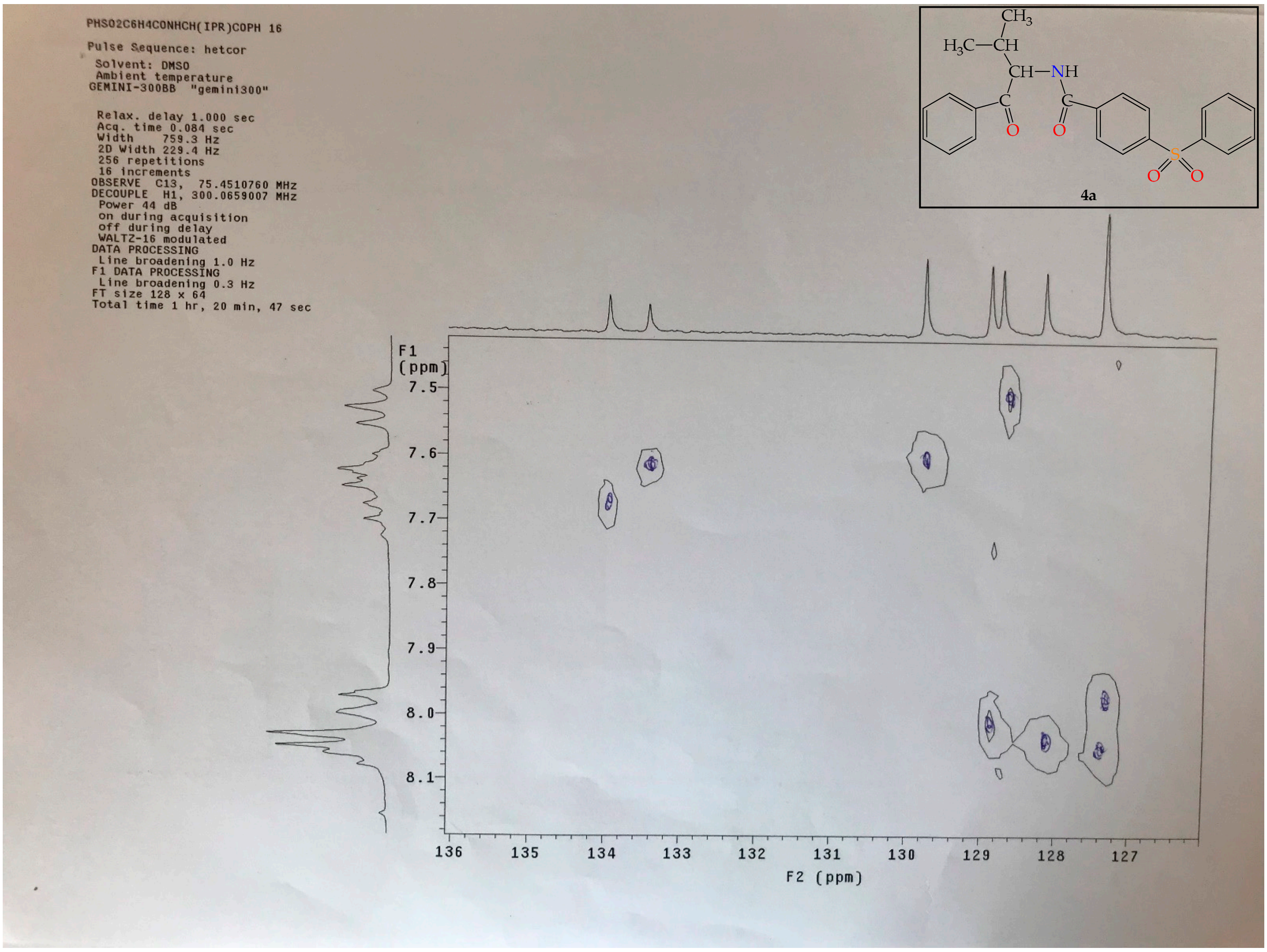

Figure S9. The 2D HETCOR spectrum of *N*-(3-methyl-1-oxo-1-phenylbutan-2-yl)-4-(phenylsulfonyl)benzamide **4a**.

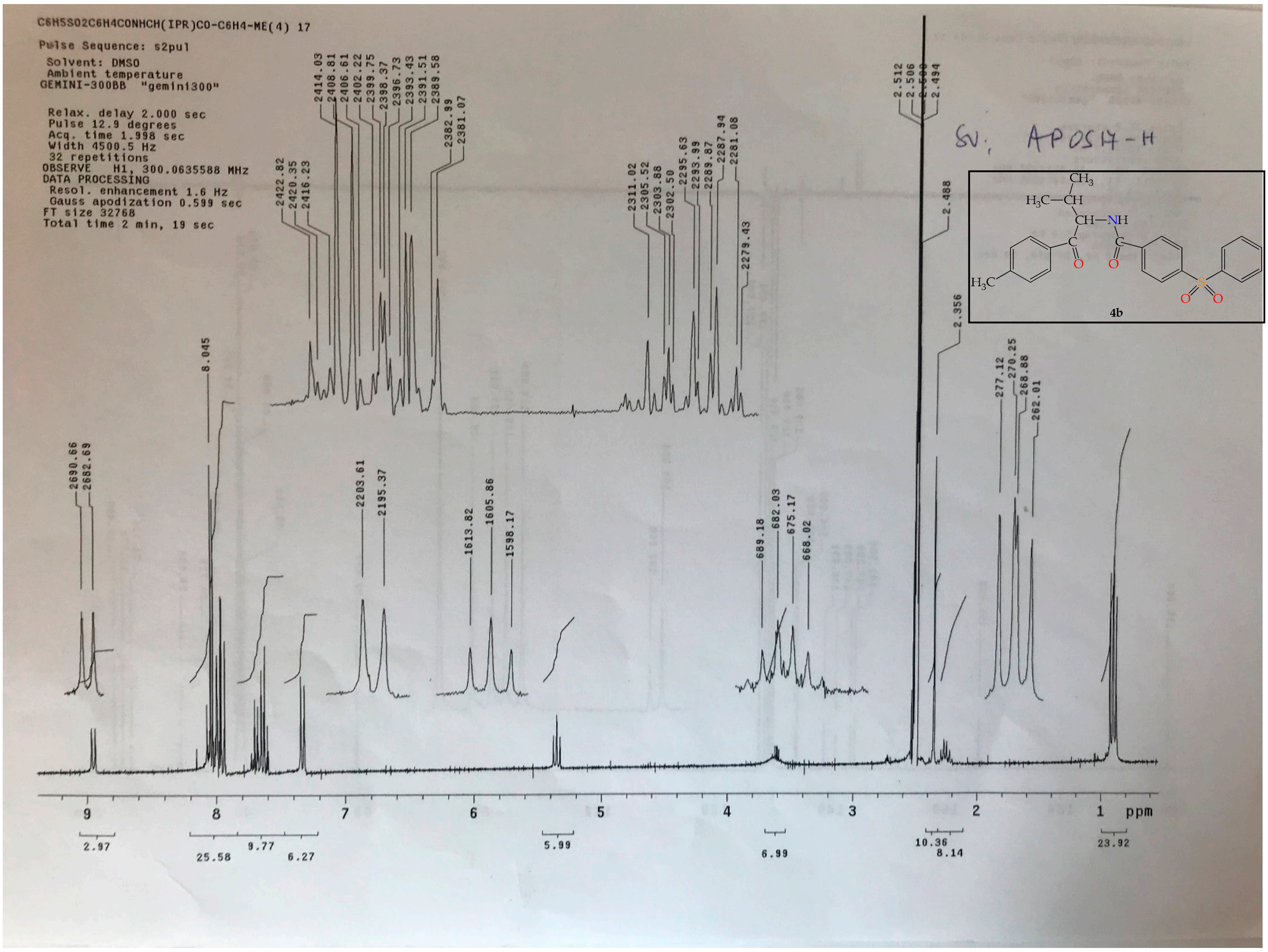

Figure S10. The ^{1}H-NMR spectrum of *N*-[3-methyl-1-oxo-1-(*p*-tolyl)butan-2-yl]-4-(phenylsulfonyl)benzamide **4b**.

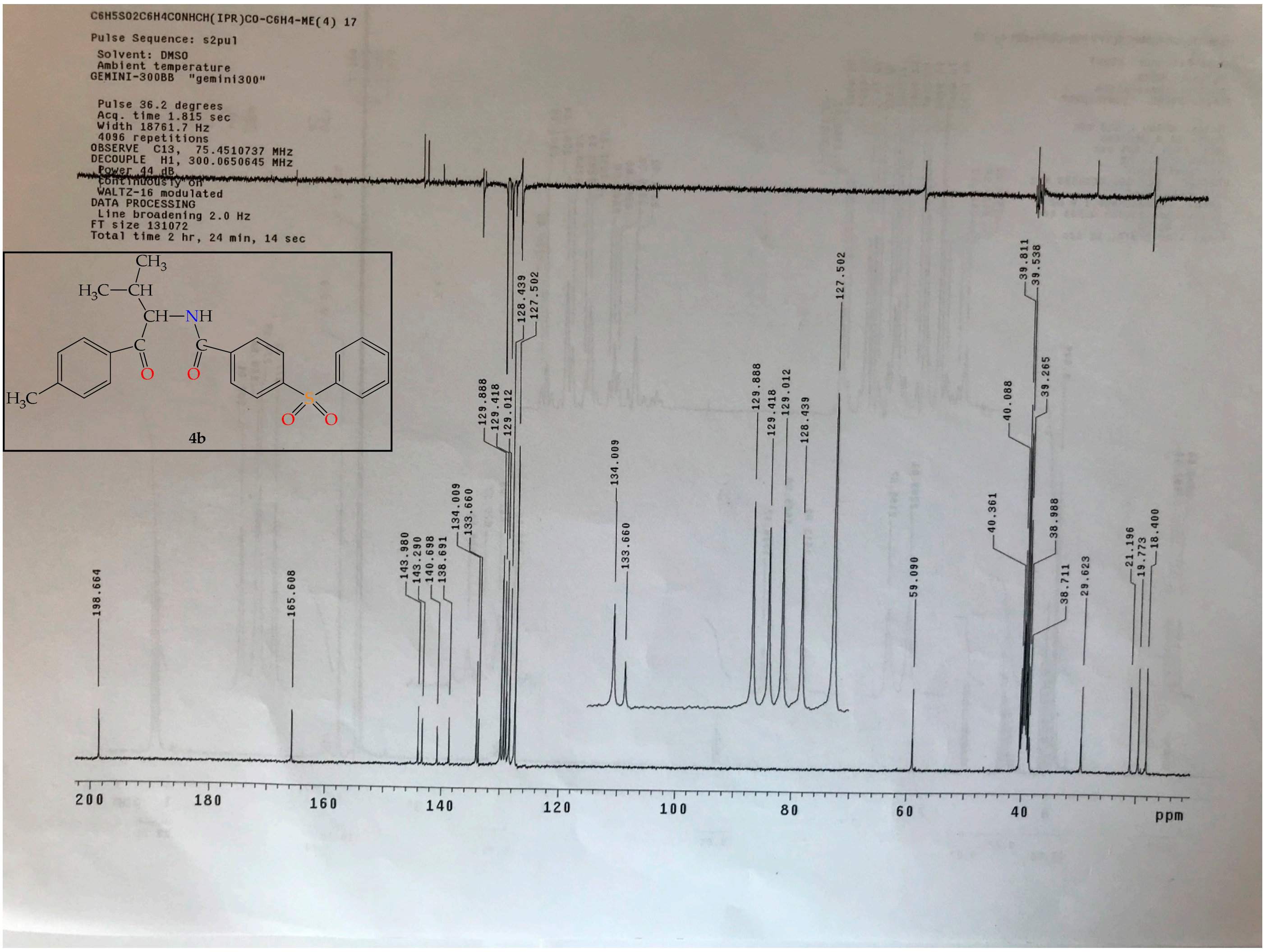

Figure S11. The ^{13}C-NMR spectrum of *N*-[3-methyl-1-oxo-1-(*p*-tolyl)butan-2-yl]-4-(phenylsulfonyl)benzamide **4b**.

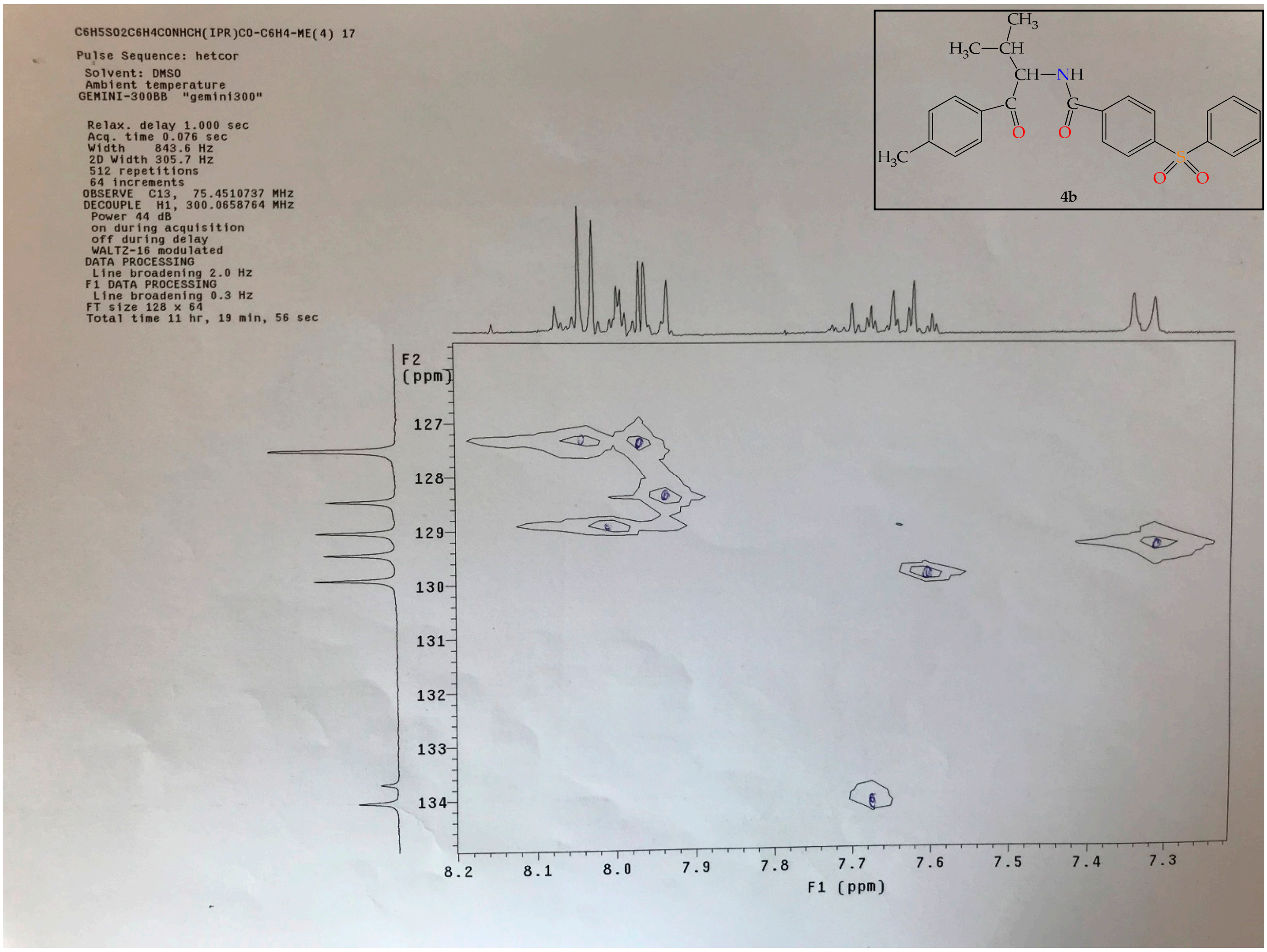

Figure S12. The 2D HETCOR spectrum of *N*-[3-methyl-1-oxo-1-(*p*-tolyl)butan-2-yl]-4-(phenylsulfonyl)benzamide **4b**.

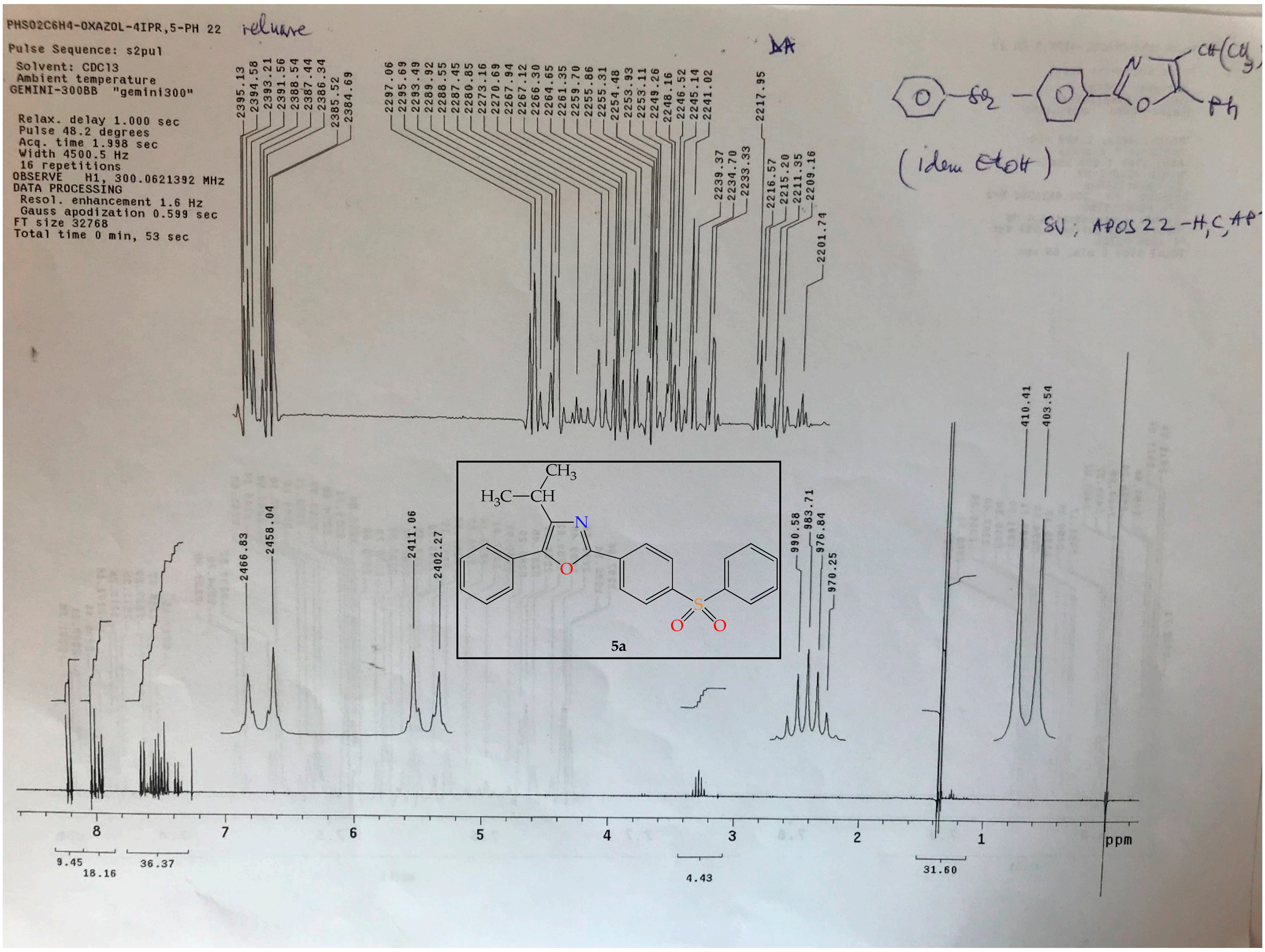

Figure S13. The ^{1}H-NMR spectrum of 4-isopropyl-5-phenyl-2-[4-(phenylsulfonyl)phenyl]-1,3-oxazole **5a**.

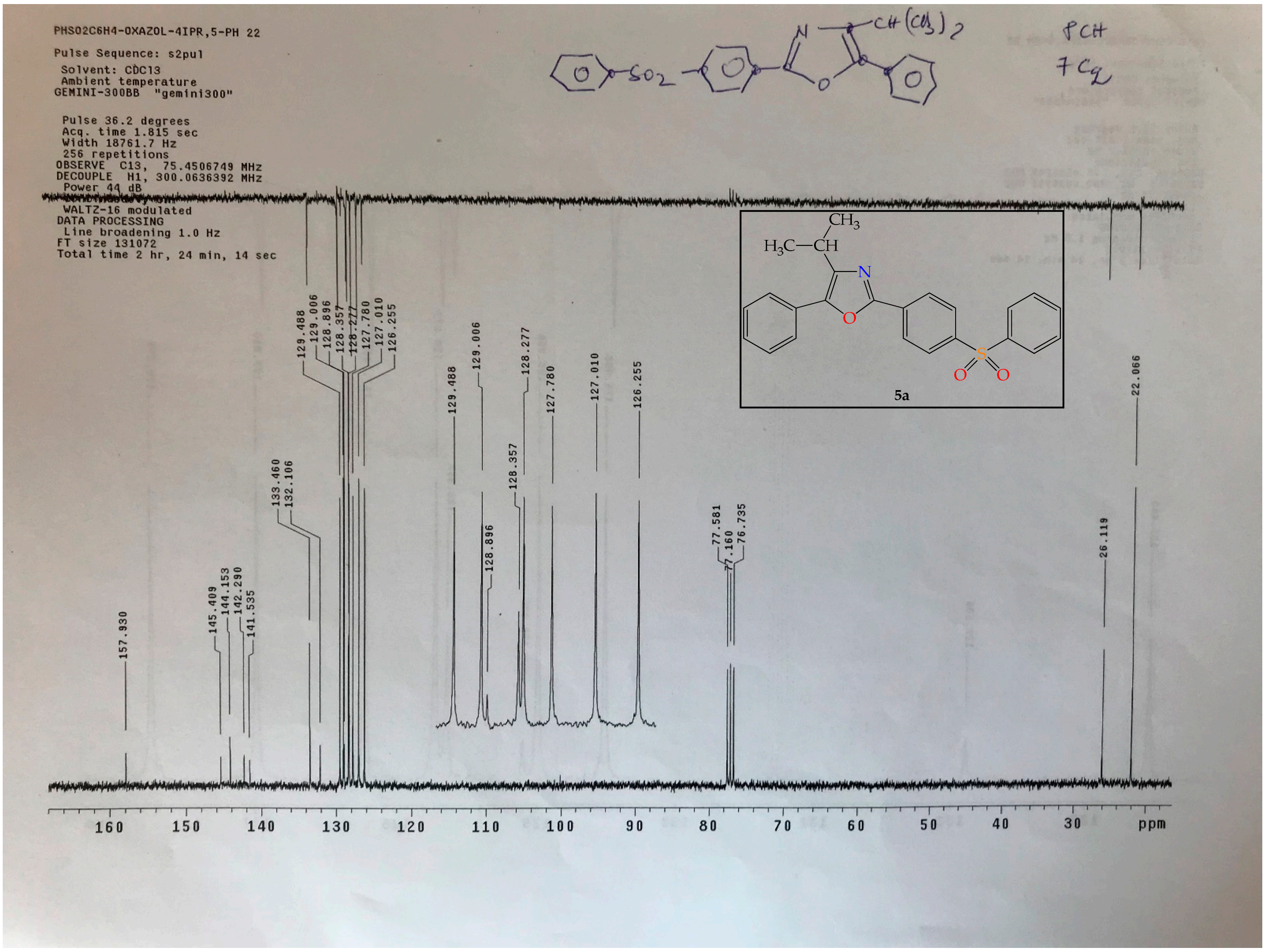

Figure S14. The ^{13}C-NMR spectrum of 4-isopropyl-5-phenyl-2-[4-(phenylsulfonyl)phenyl]-1,3-oxazole **5a**.

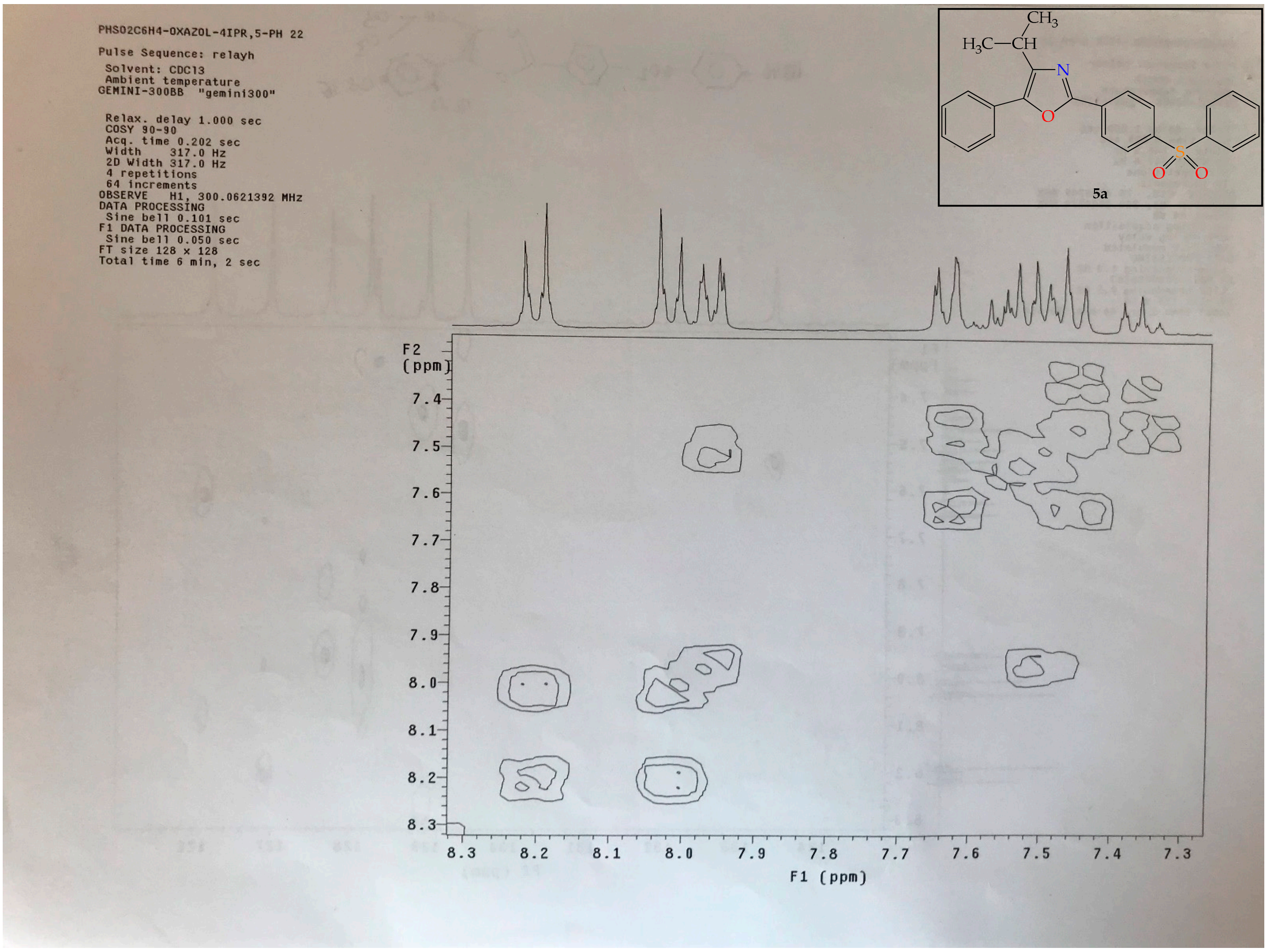

Figure S15. The 2D COSY spectrum of 4-isopropyl-5-phenyl-2-[4-(phenylsulfonyl)phenyl]-1,3-oxazole **5a**.

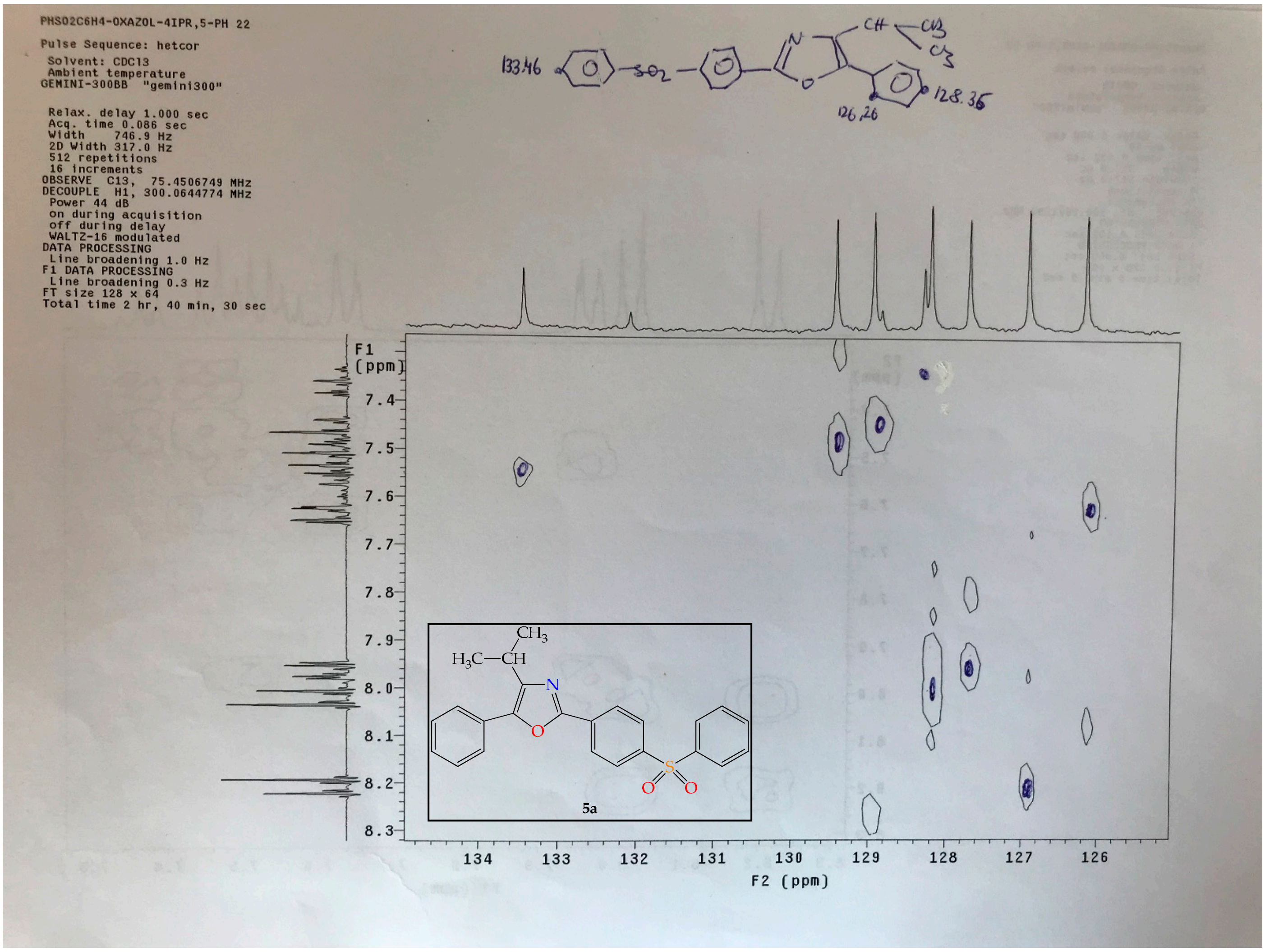

Figure S16. The 2D HETCOR spectrum of 4-isopropyl-5-phenyl-2-[4-(phenylsulfonyl)phenyl]-1,3-oxazole **5a**.

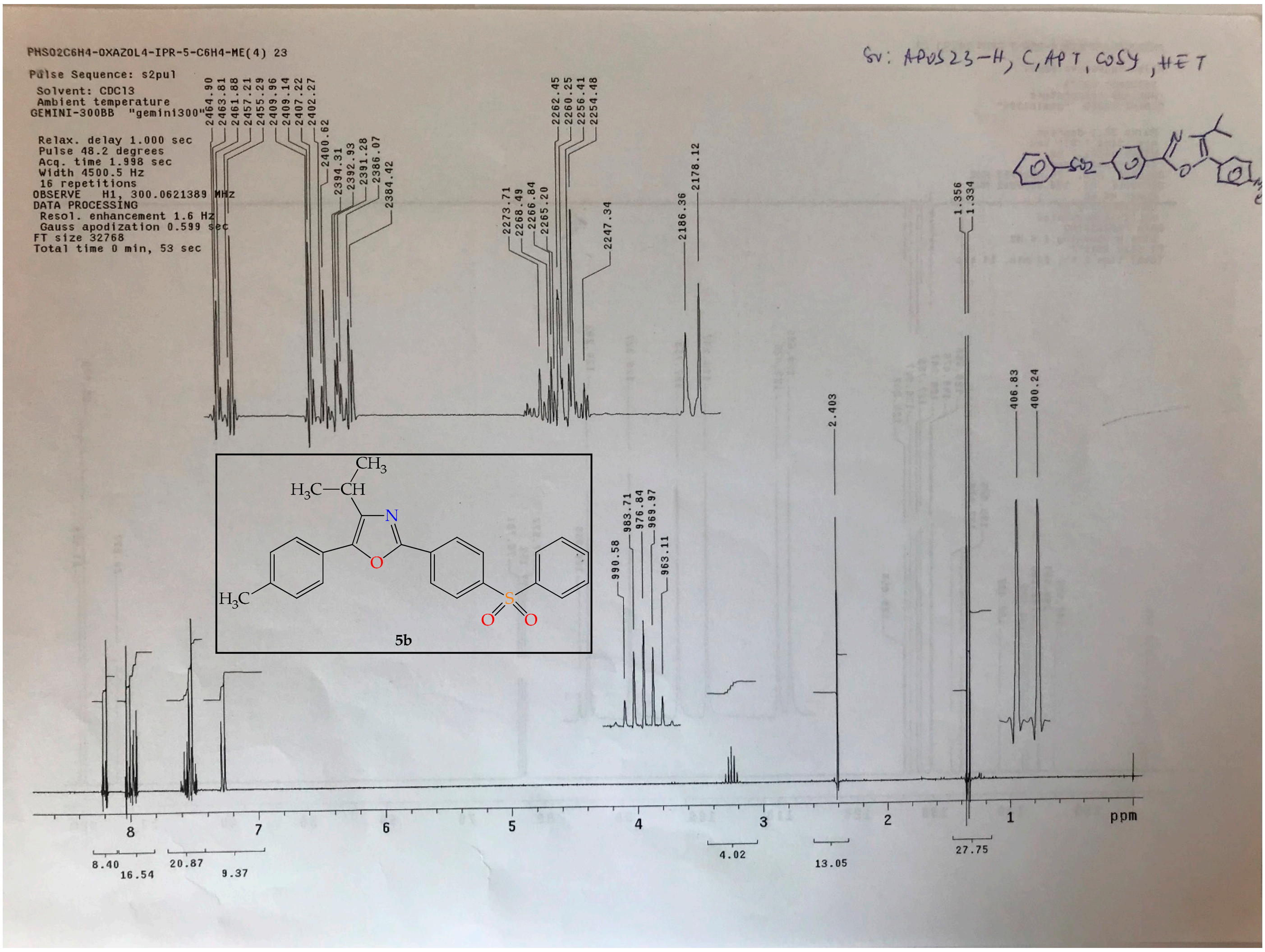

Figure S17. The ^{1}H-NMR spectrum of 4-isopropyl-2-[4-(phenylsulfonyl)phenyl]-5-(*p*-tolyl)-1,3-oxazole **5b**.

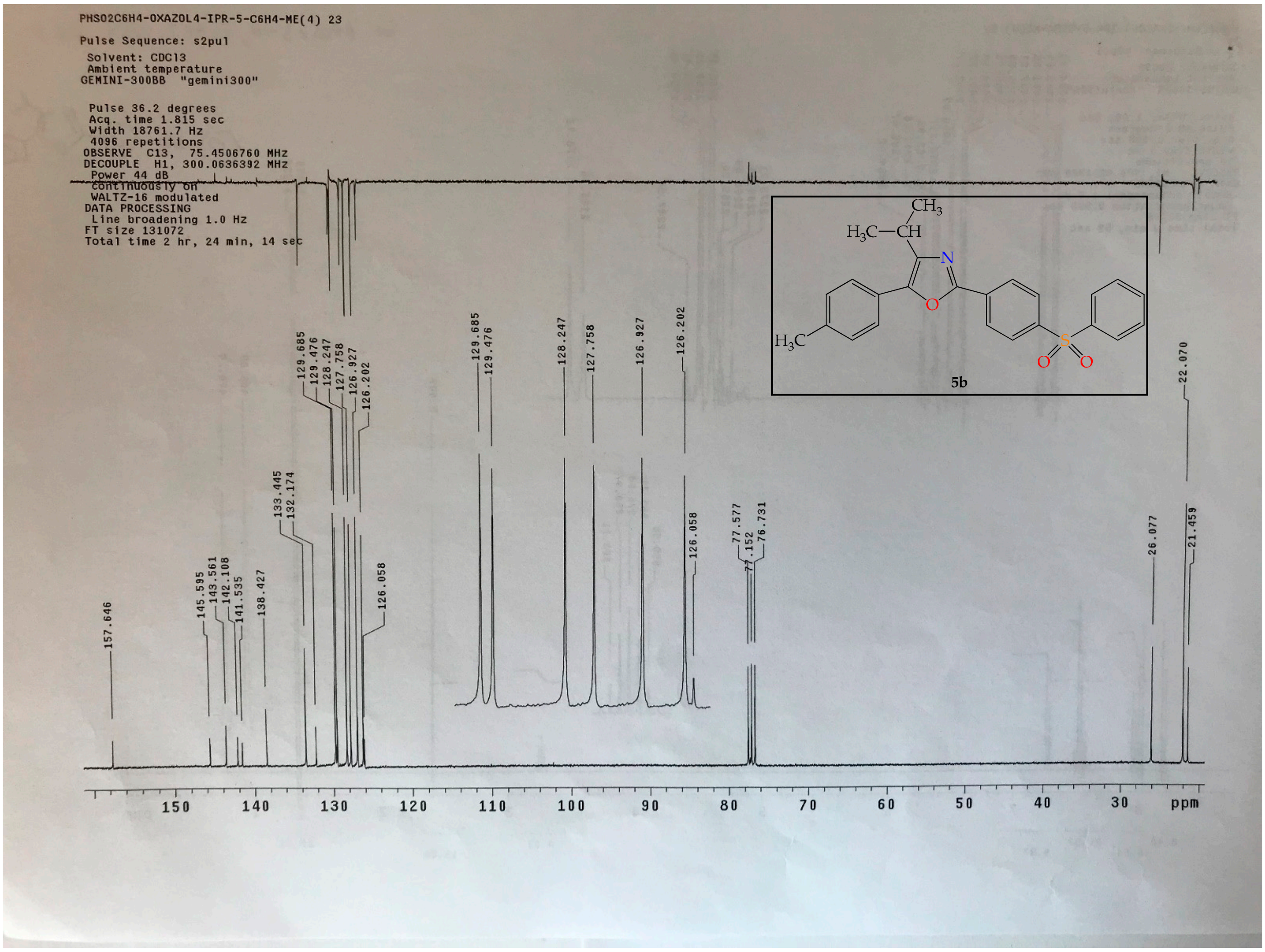

Figure S18. The ^{13}C-NMR spectrum of 4-isopropyl-2-[4-(phenylsulfonyl)phenyl]-5-(*p*-tolyl)-1,3-oxazole **5b**.

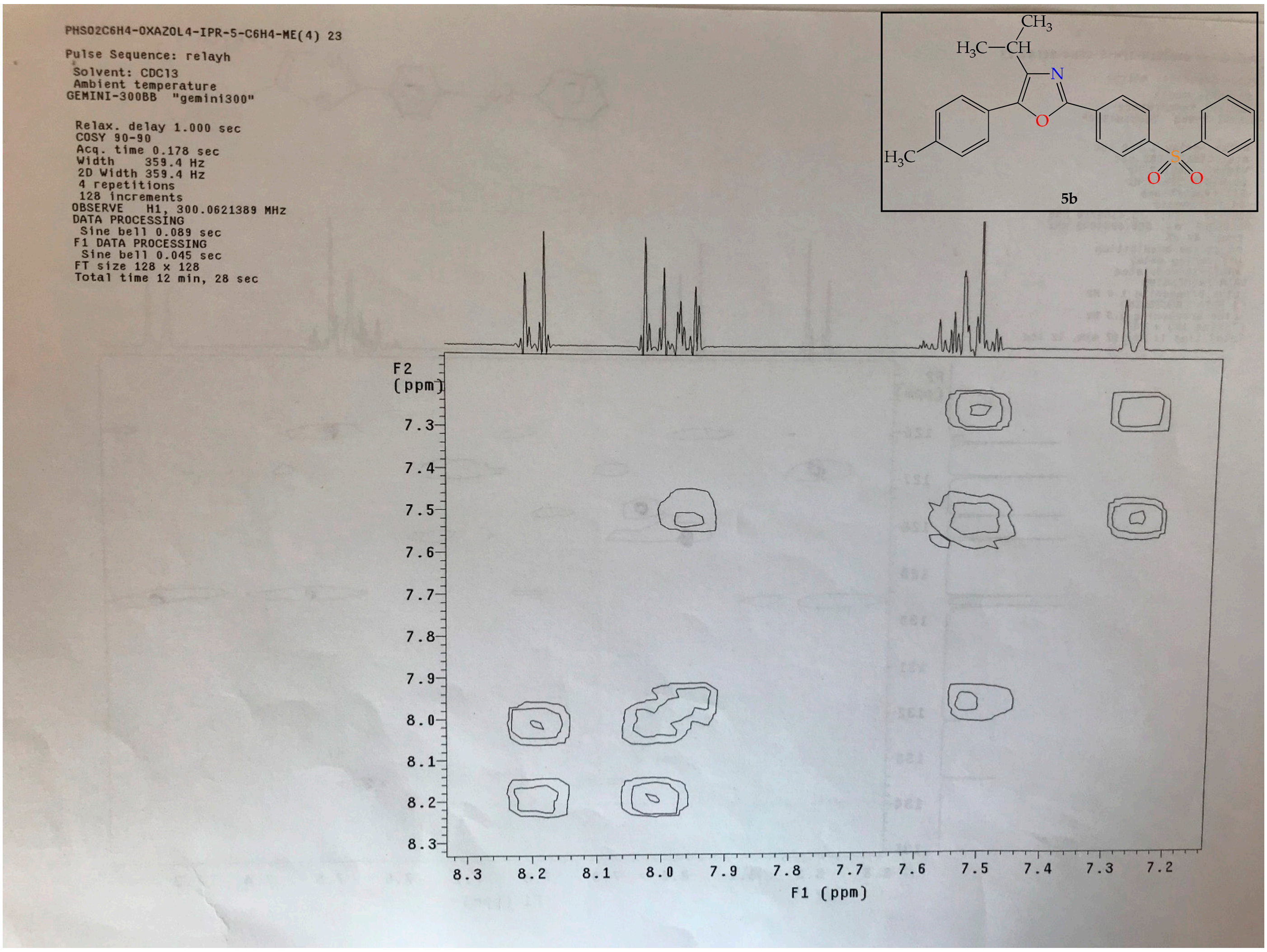

Figure S19. The 2D COSY spectrum of 4-isopropyl-2-[4-(phenylsulfonyl)phenyl]-5-(*p*-tolyl)-1,3-oxazole **5b**.

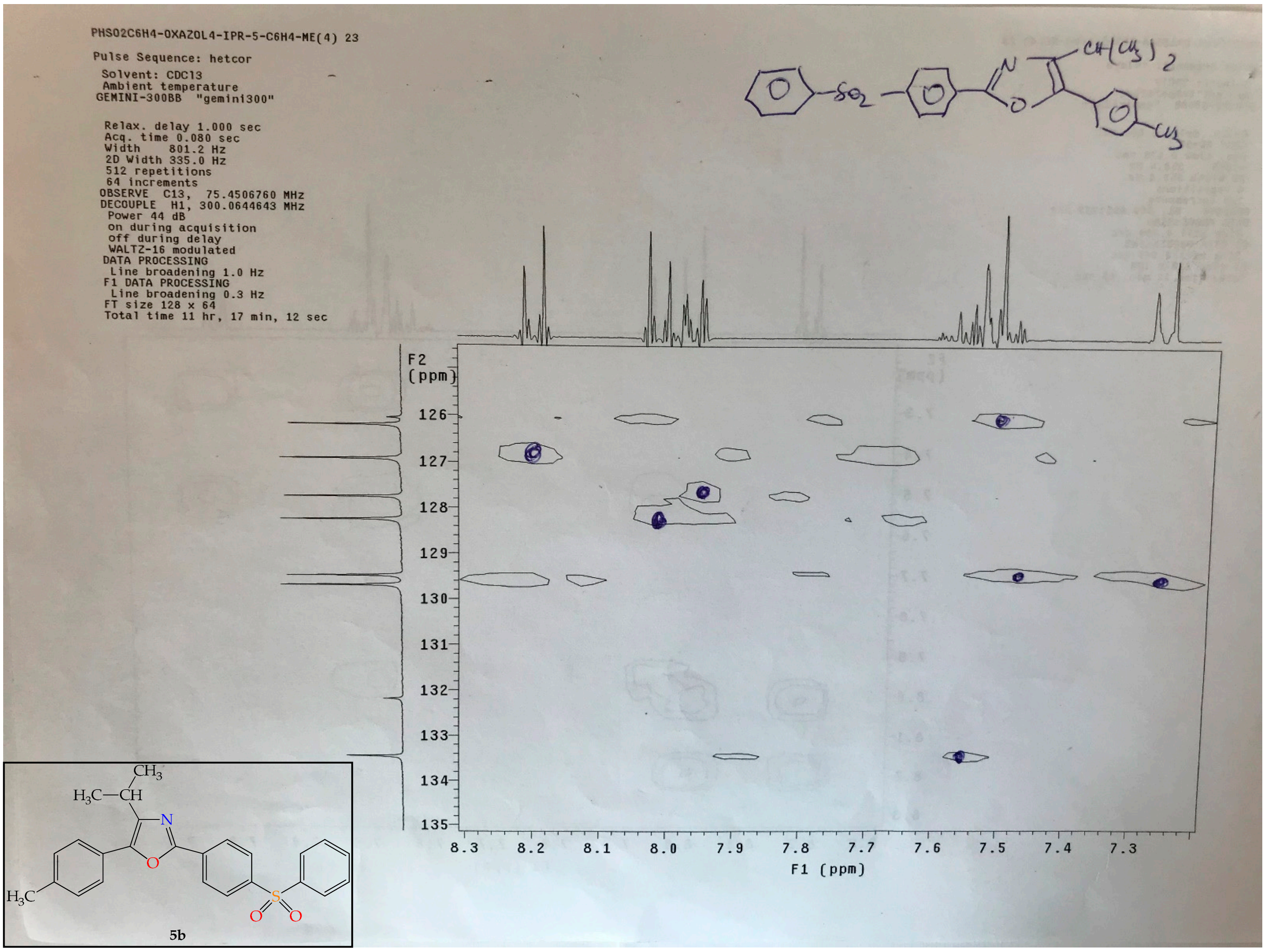

Figure S20. The 2D HETCOR spectrum of 4-isopropyl-2-[4-(phenylsulfonyl)phenyl]-5-(*p*-tolyl)-1,3-oxazole **5b**.

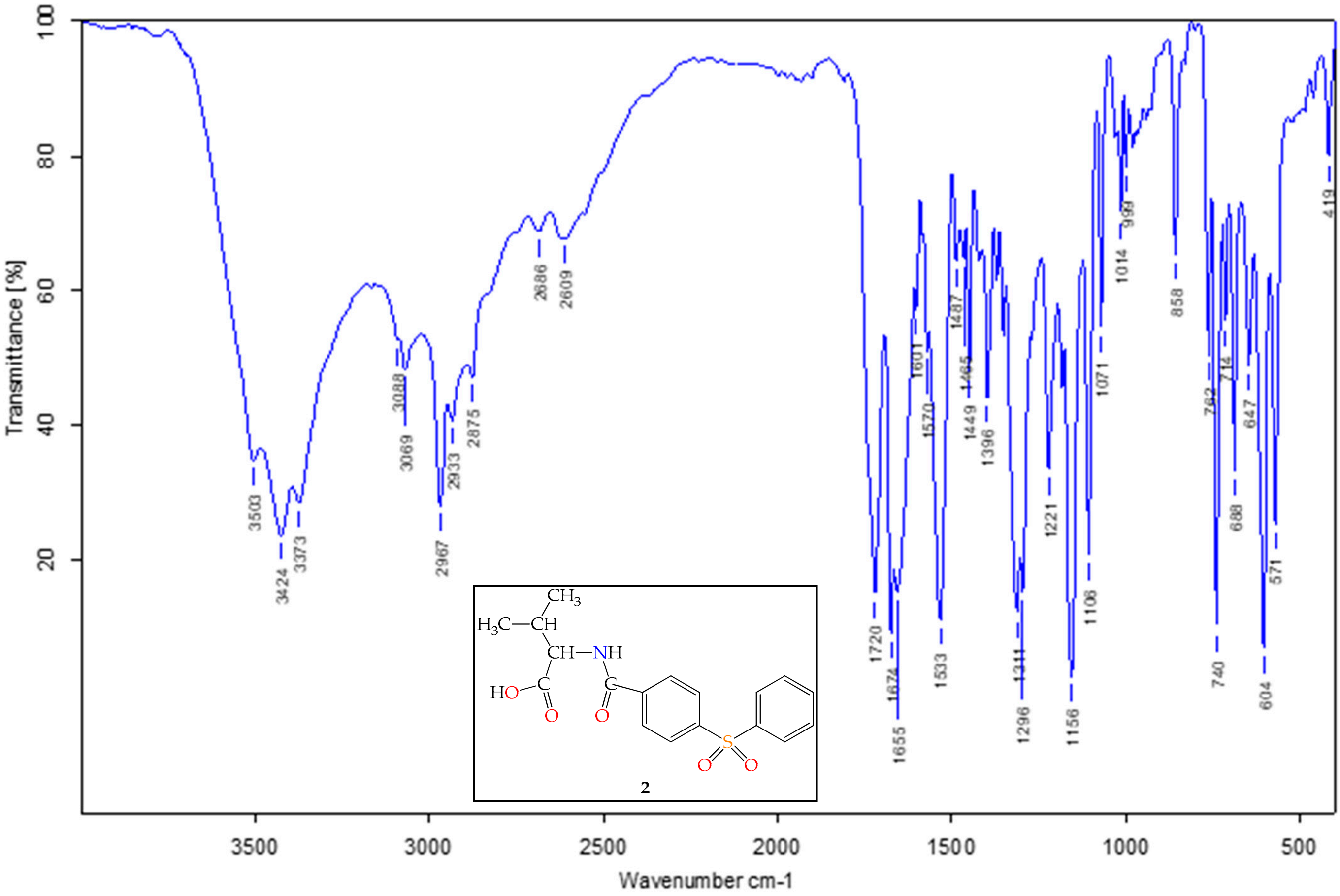

Figure S21. The FT-IR spectrum of 3-methyl-2-[4-(phenylsulfonyl)benzamido]butanoic acid **2**.

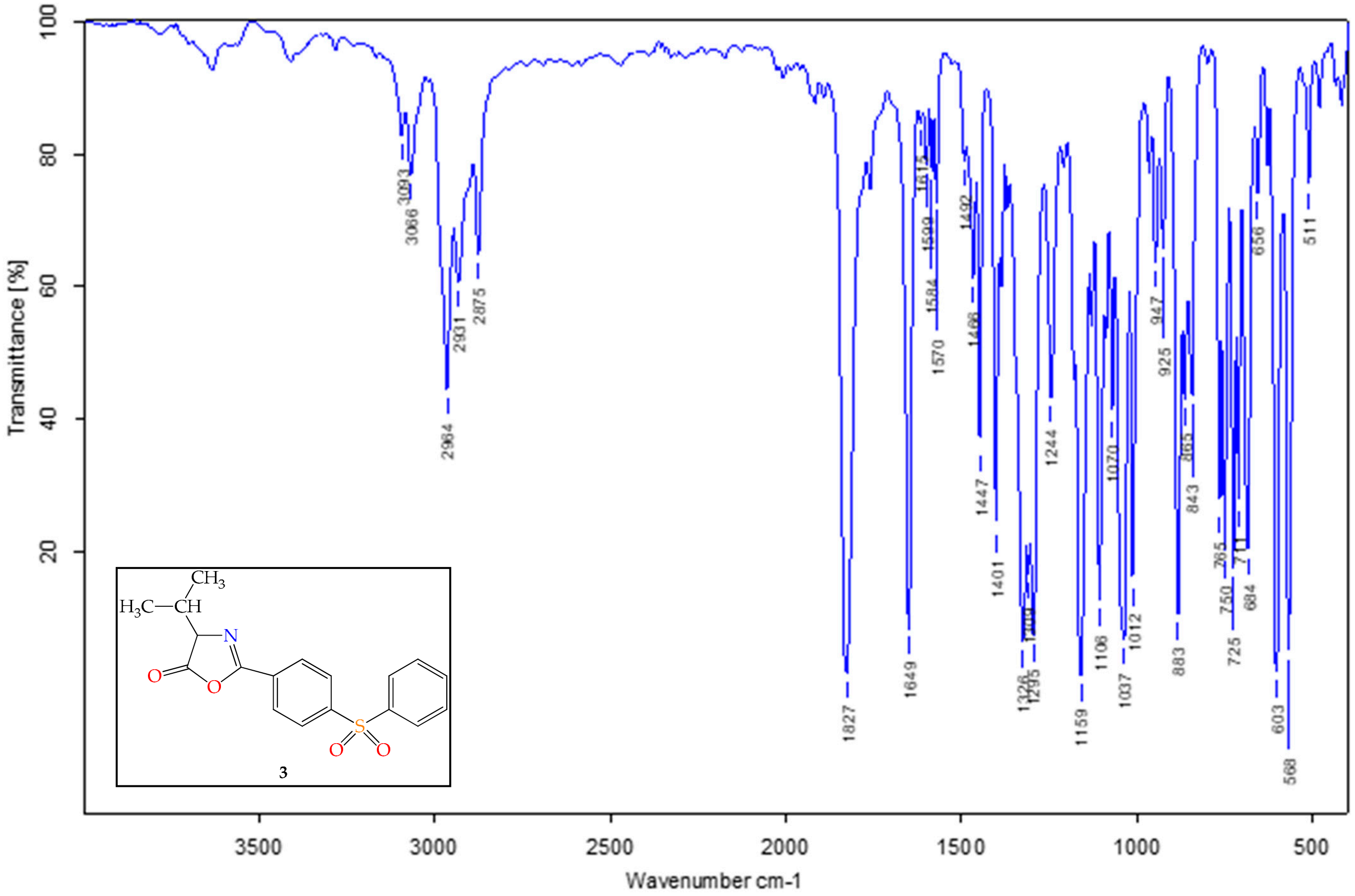

Figure S22. The FT-IR spectrum of 4-isopropyl-2-[4-(phenylsulfonyl)phenyl]-1,3-oxazol-5(4*H*)-one **3**.

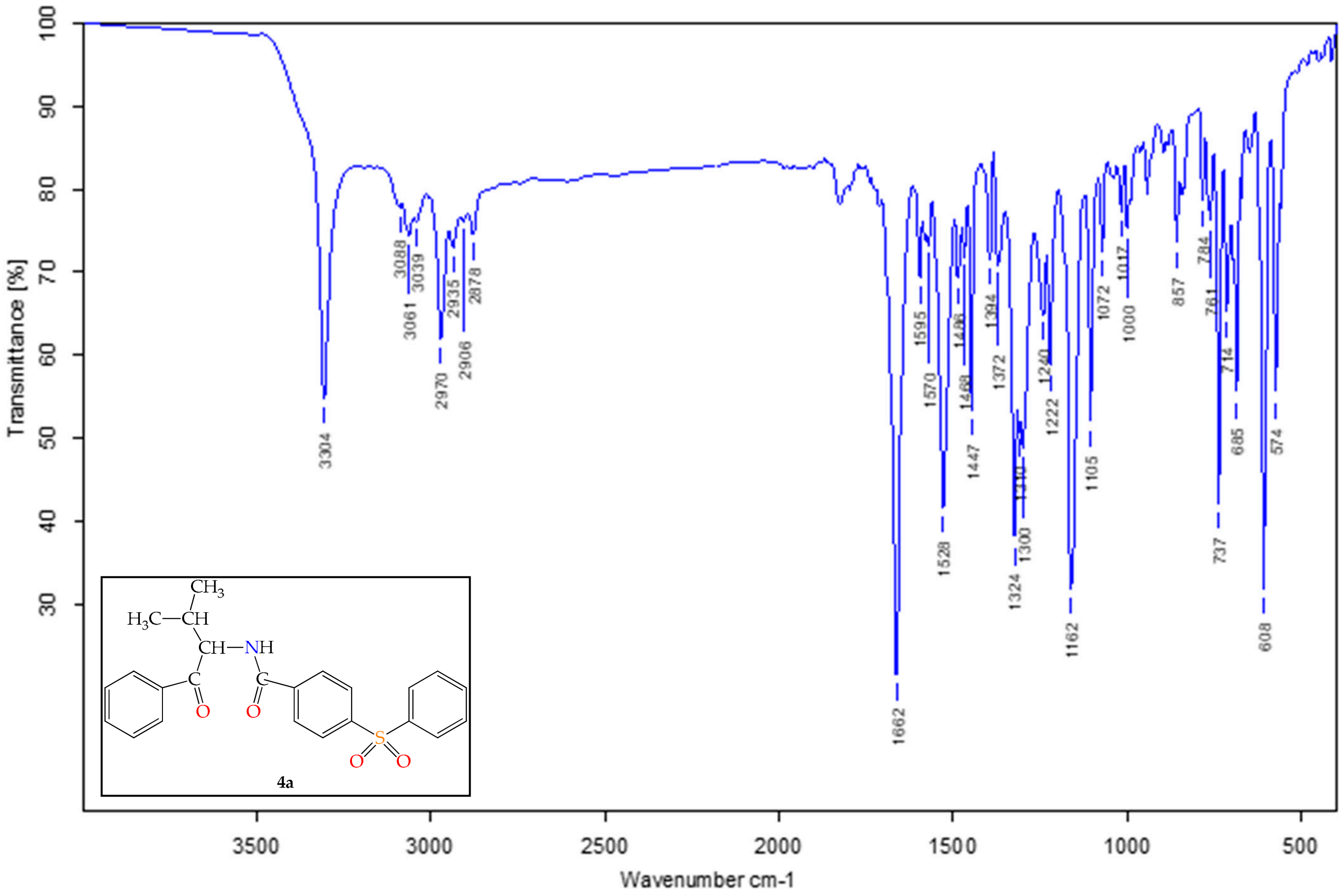

Figure S23. The FT-IR spectrum of *N*-(3-methyl-1-oxo-1-phenylbutan-2-yl)-4-(phenylsulfonyl)benzamide **4a**.

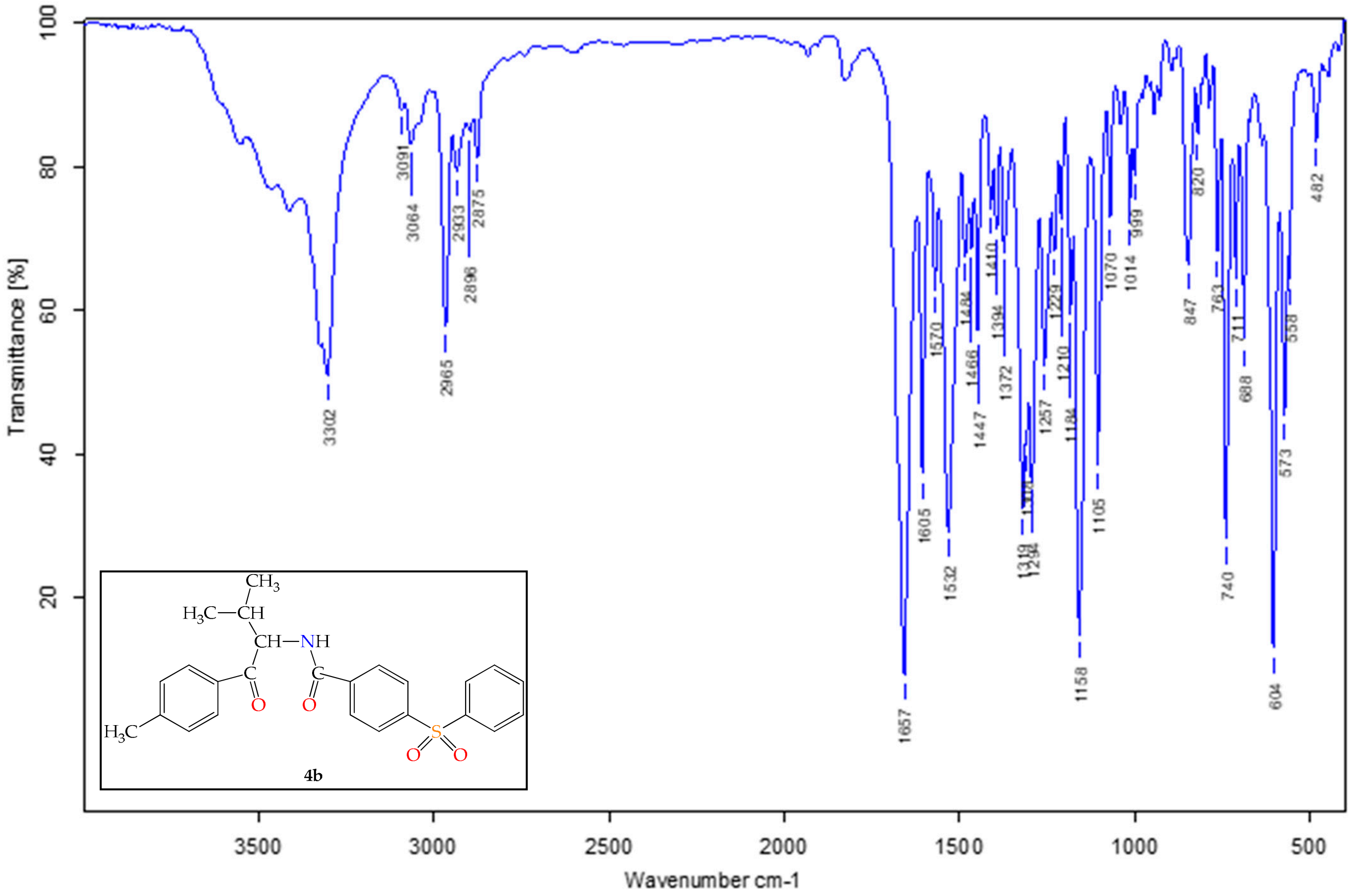

Figure S24. The FT-IR spectrum of *N*-[3-methyl-1-oxo-1-(*p*-tolyl)butan-2-yl]-4-(phenylsulfonyl)benzamide **4b**.

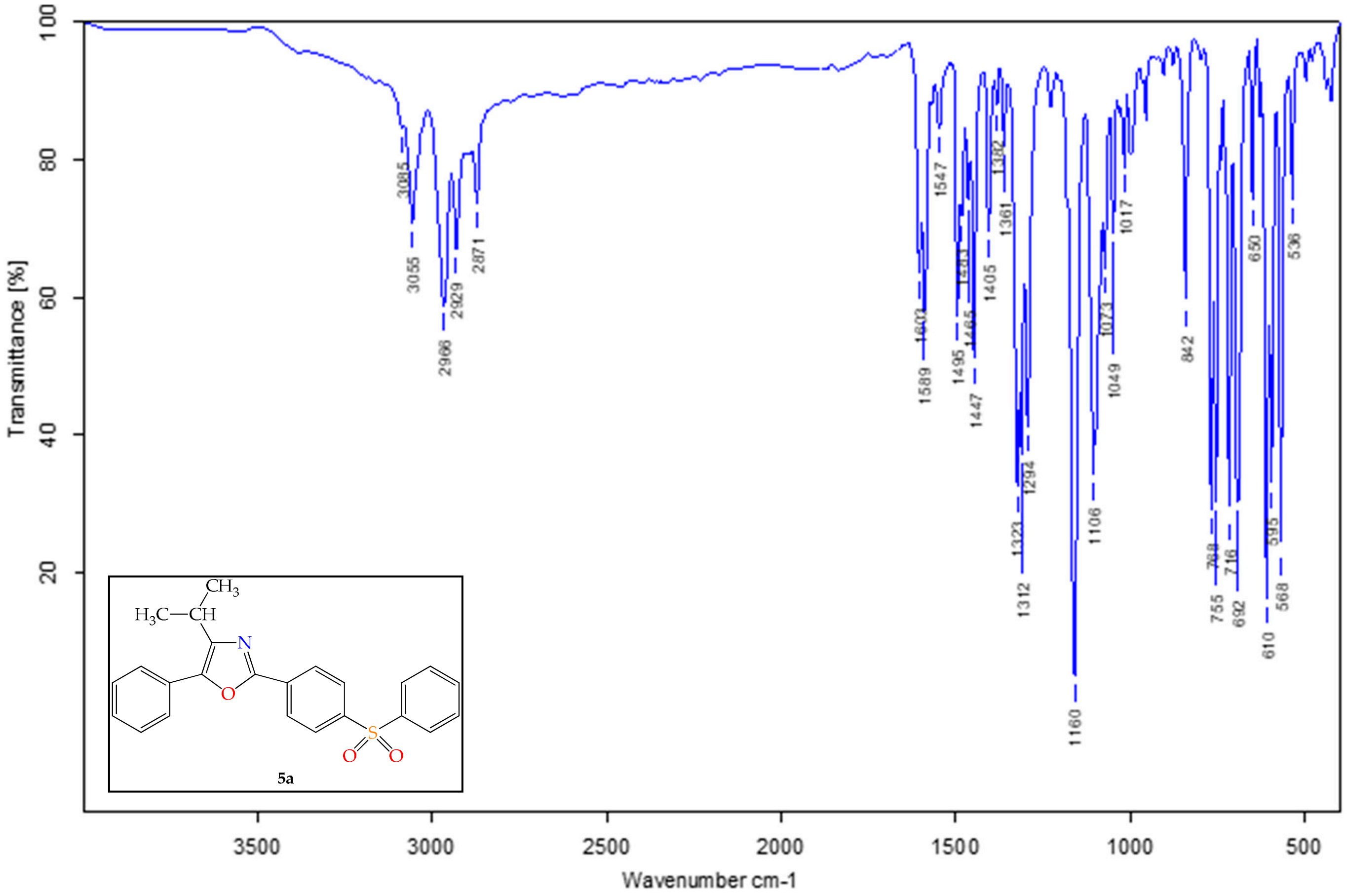

Figure S25. The FT-IR spectrum of 4-isopropyl-5-phenyl-2-[4-(phenylsulfonyl)phenyl]-1,3-oxazole **5a**.

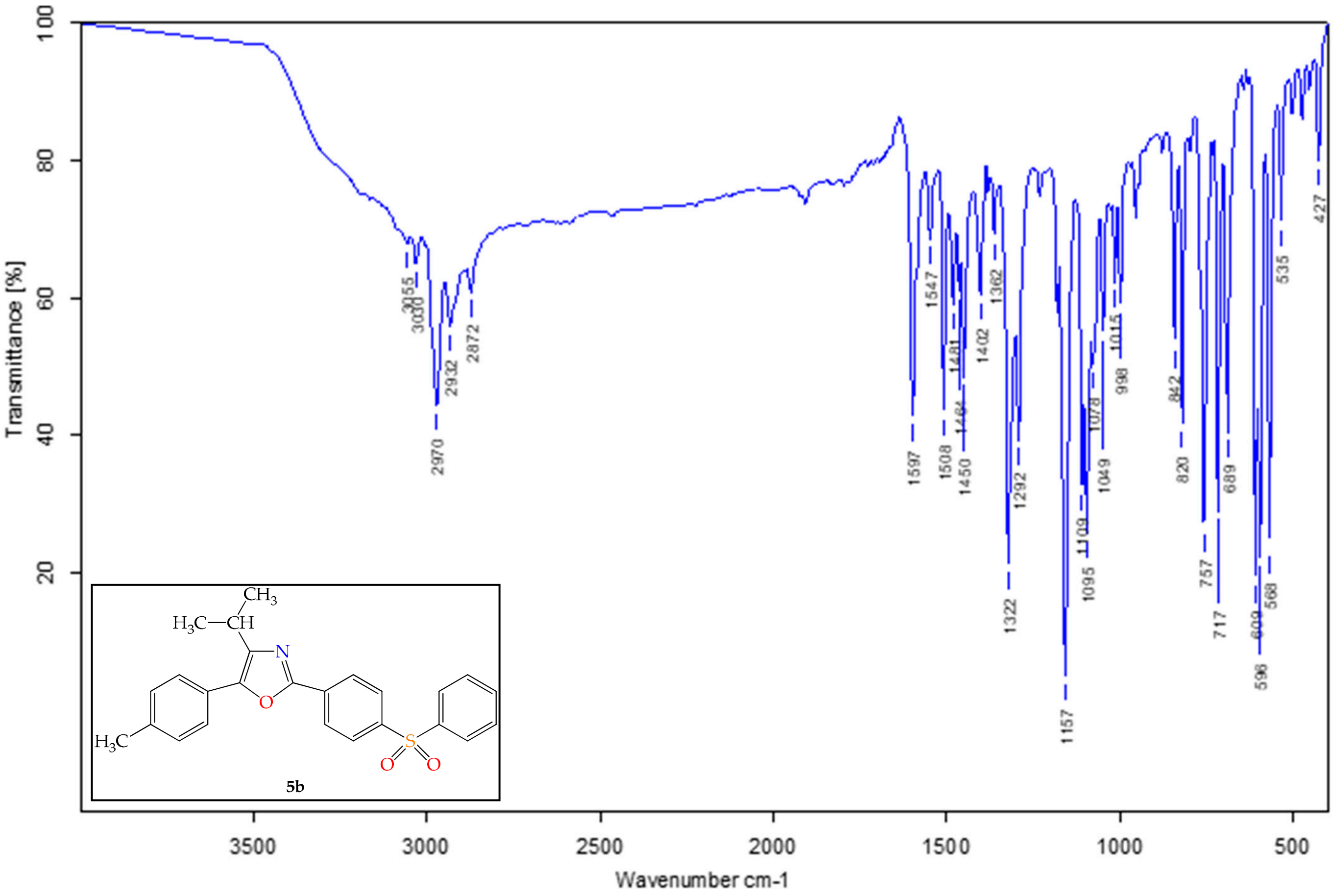

Figure S26. The FT-IR spectrum of 4-isopropyl-2-[4-(phenylsulfonyl)phenyl]-5-(*p*-tolyl)-1,3-oxazole **5b**.

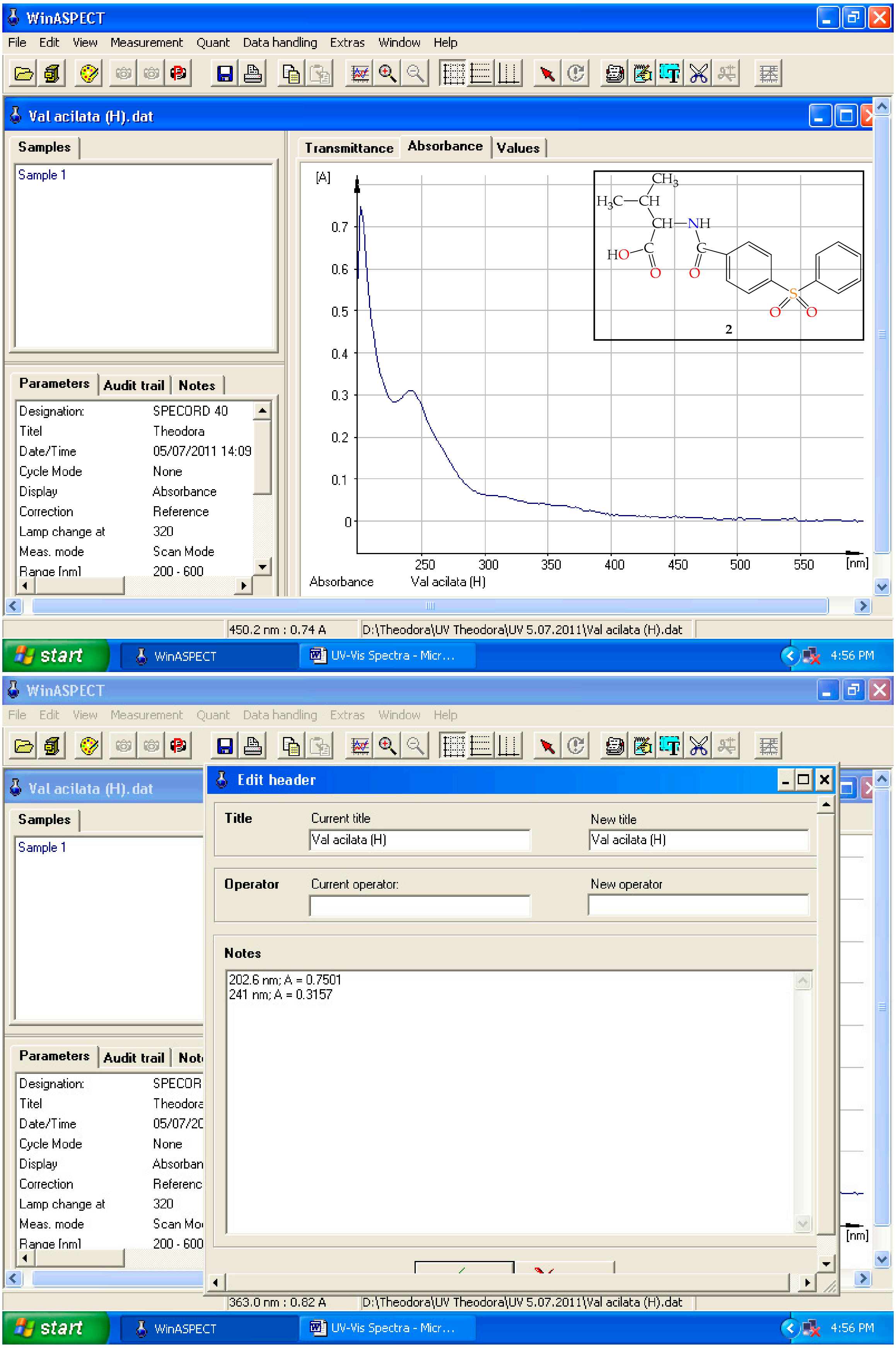

Figure S27. The UV-Vis spectrum of 3-methyl-2-[4-(phenylsulfonyl)benzamido]butanoic acid **2**.

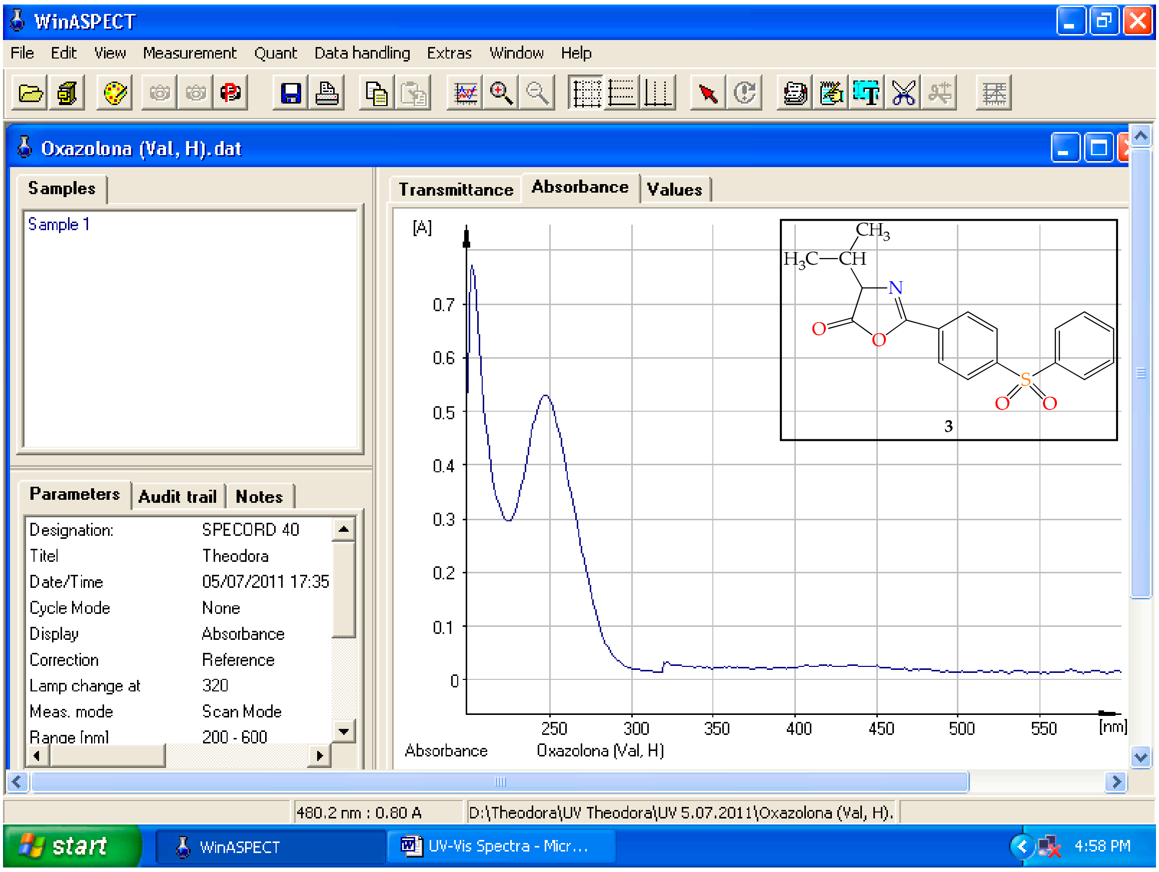

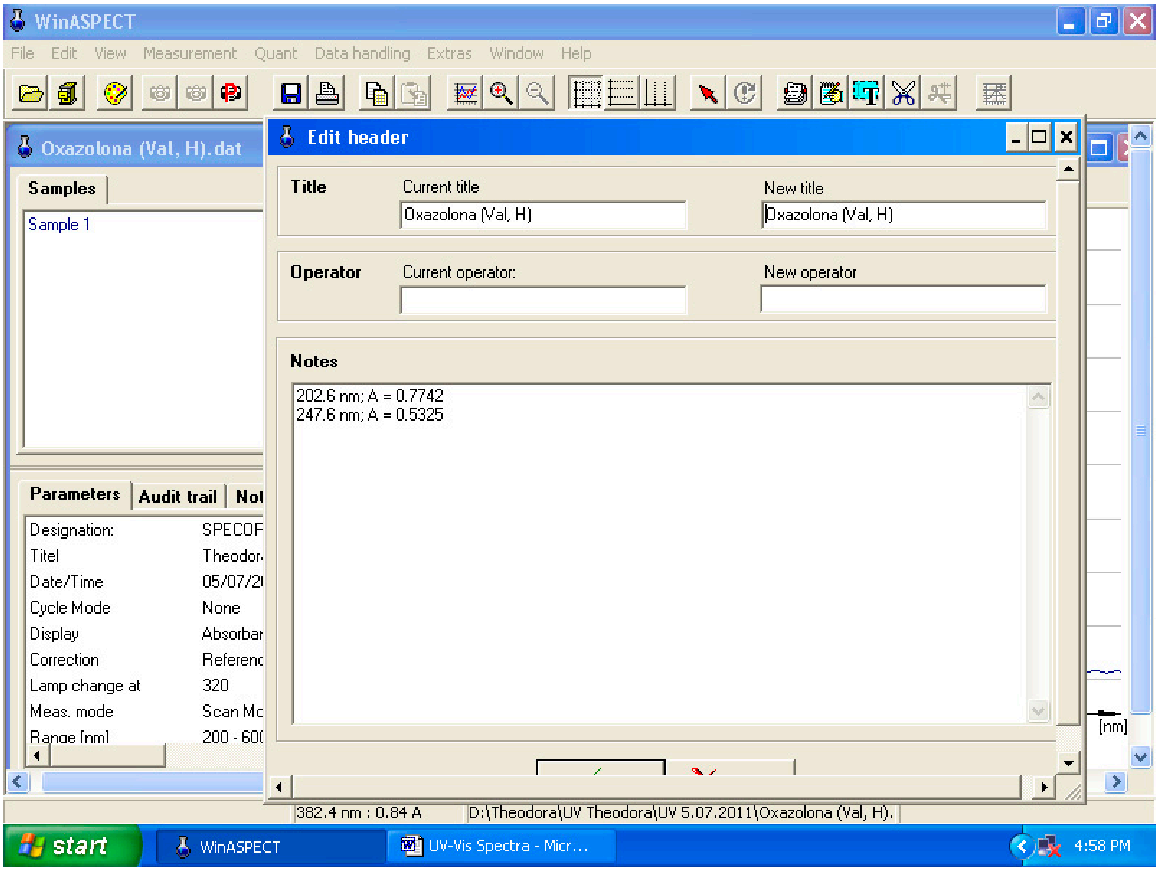

Figure S28. The UV-Vis spectrum of 4-isopropyl-2-[4-(phenylsulfonyl)phenyl]-1,3-oxazol-5(4*H*)-one **3**.

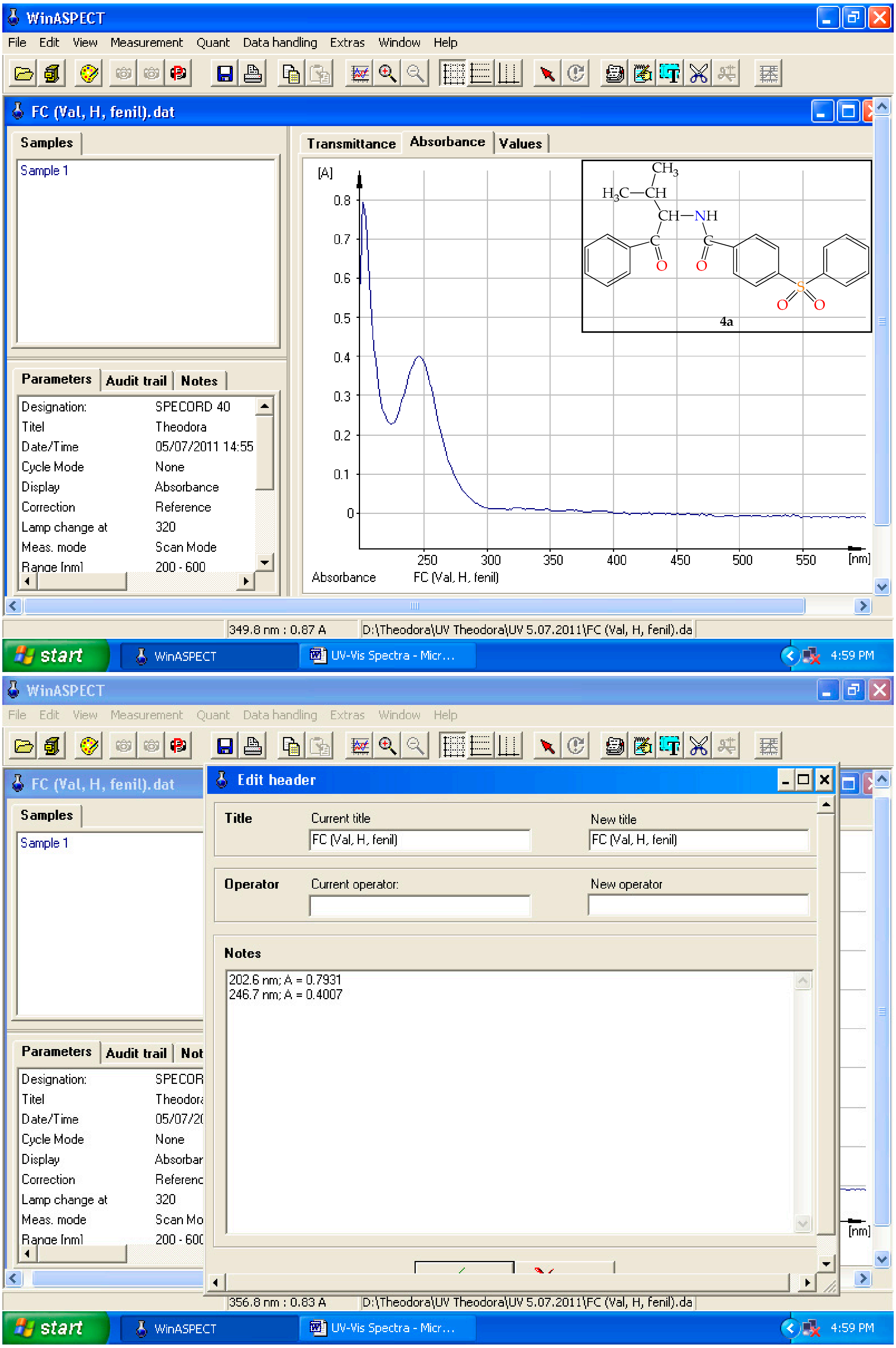

Figure S29. The UV-Vis spectrum of *N*-(3-methyl-1-oxo-1-phenylbutan-2-yl)-4-(phenylsulfonyl)benzamide **4a**.

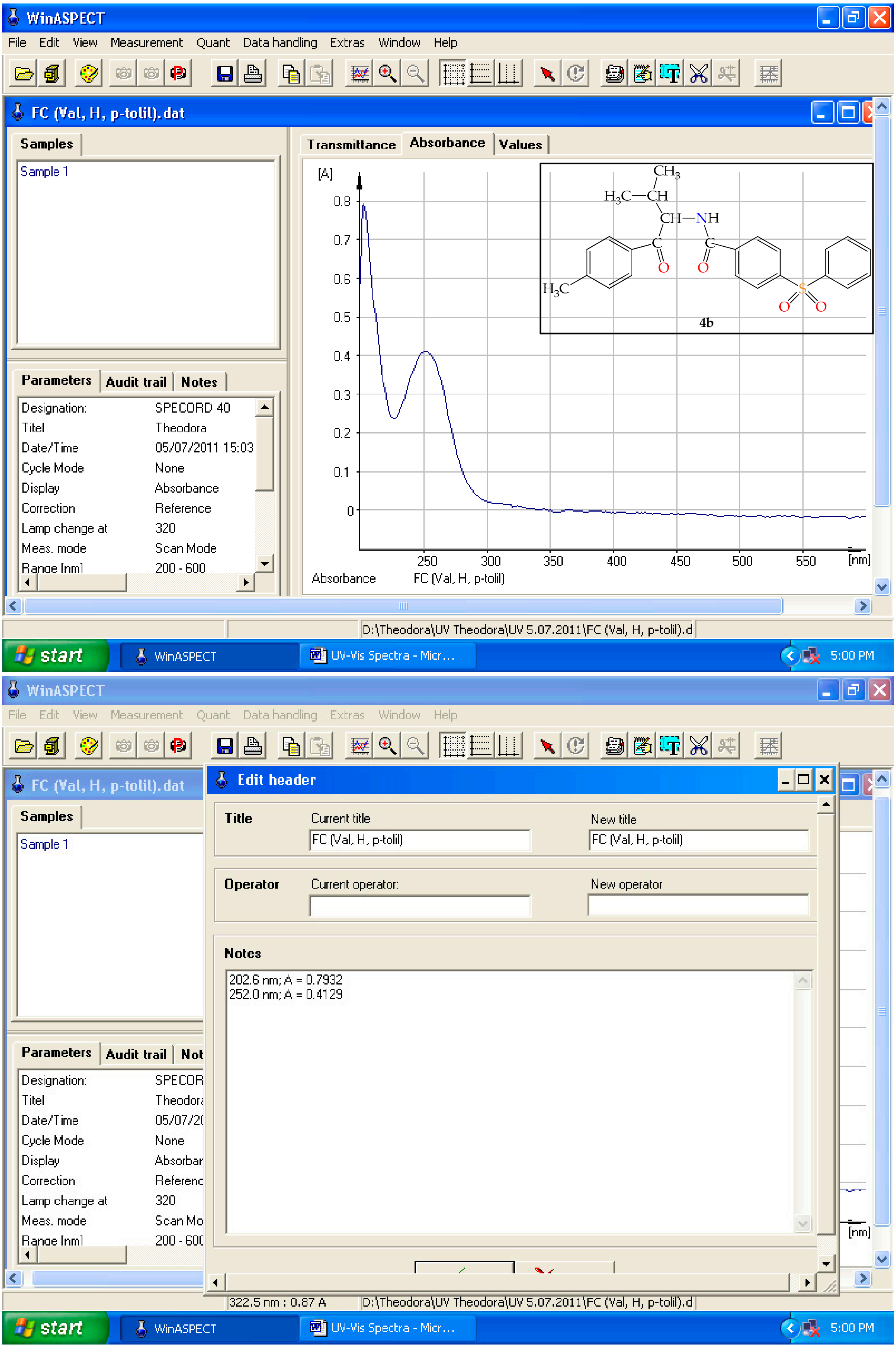

Figure S30. The UV-Vis spectrum of *N*-[3-methyl-1-oxo-1-(*p*-tolyl)butan-2-yl]-4-(phenylsulfonyl)benzamide **4b**.

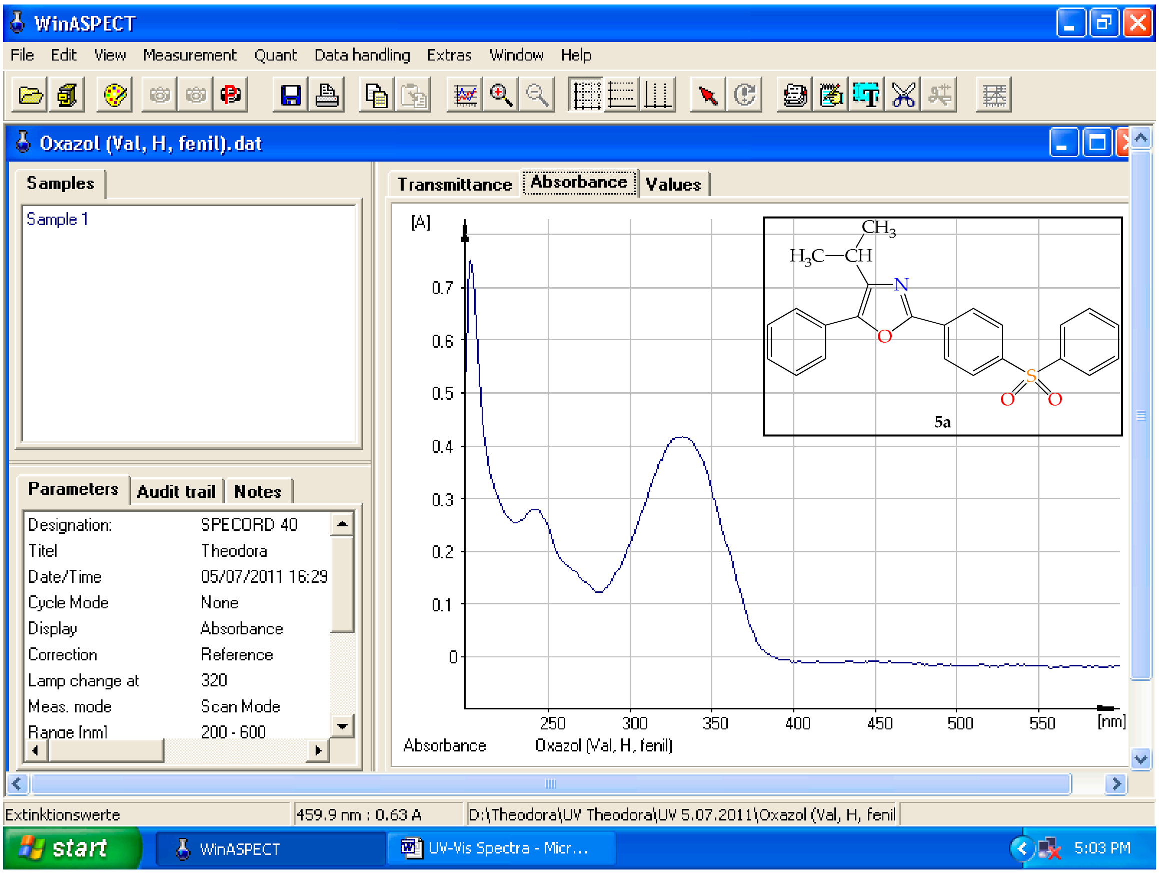

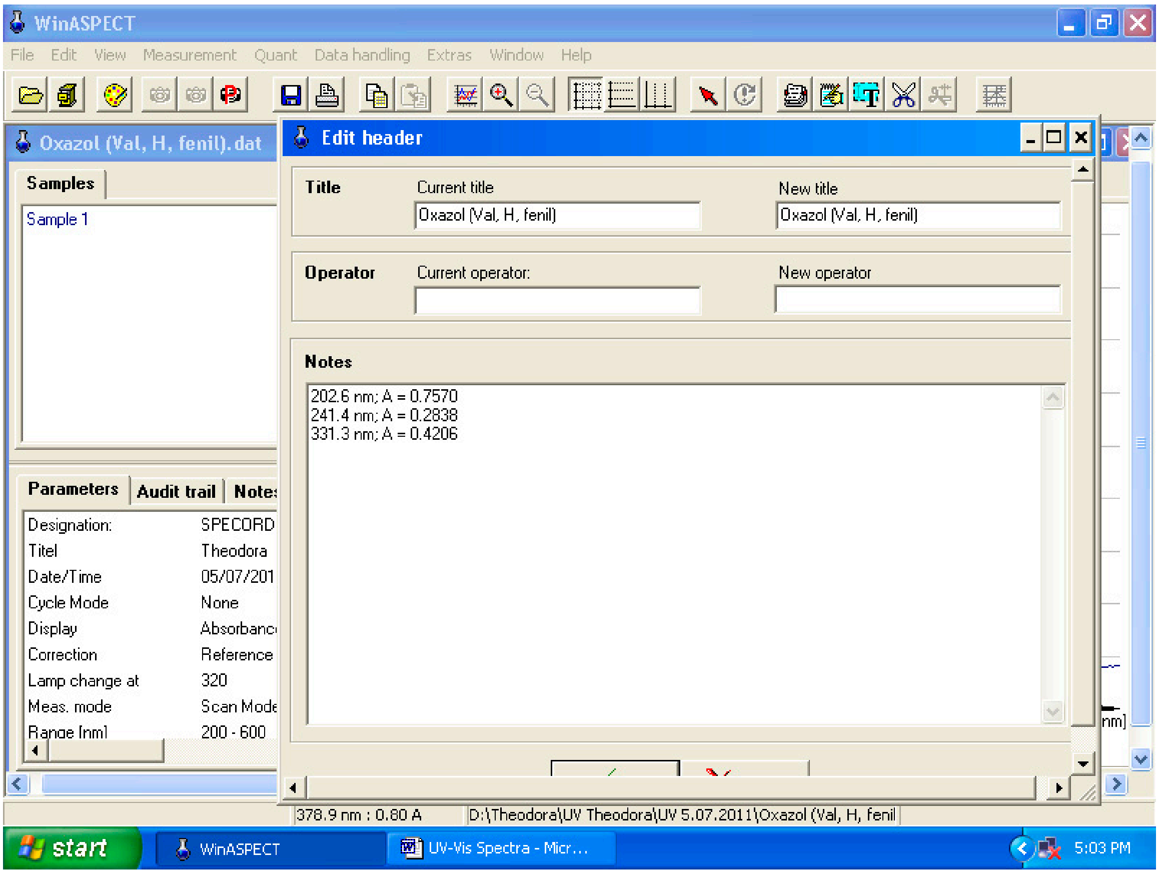

Figure S31. The UV-Vis spectrum of 4-isopropyl-5-phenyl-2-[4-(phenylsulfonyl)phenyl]-1,3-oxazole **5a**.

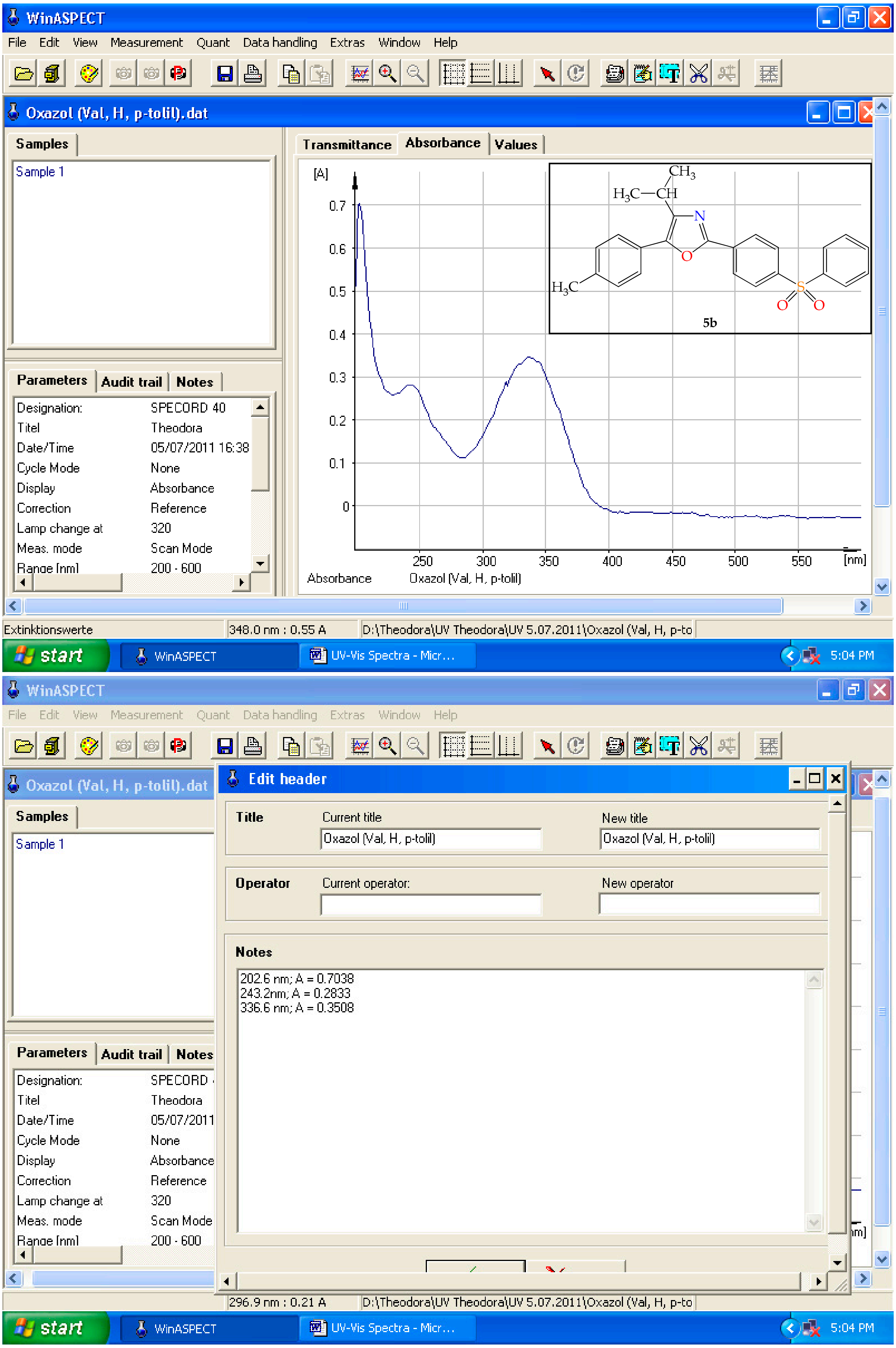

Figure S32. The UV-Vis spectrum of 4-isopropyl-2-[4-(phenylsulfonyl)phenyl]-5-(*p*-tolyl)-1,3-oxazole **5b**.

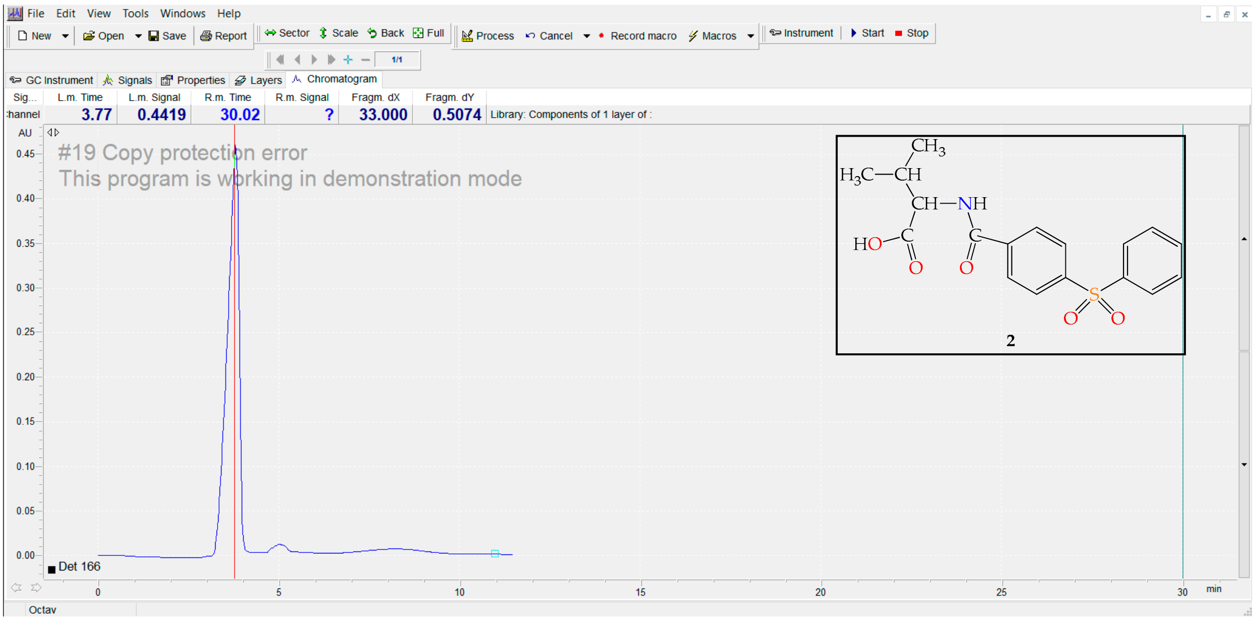

Figure S33. The RP-HPLC chromatogram of 3-methyl-2-[4-(phenylsulfonyl)benzamido]butanoic acid **2**.

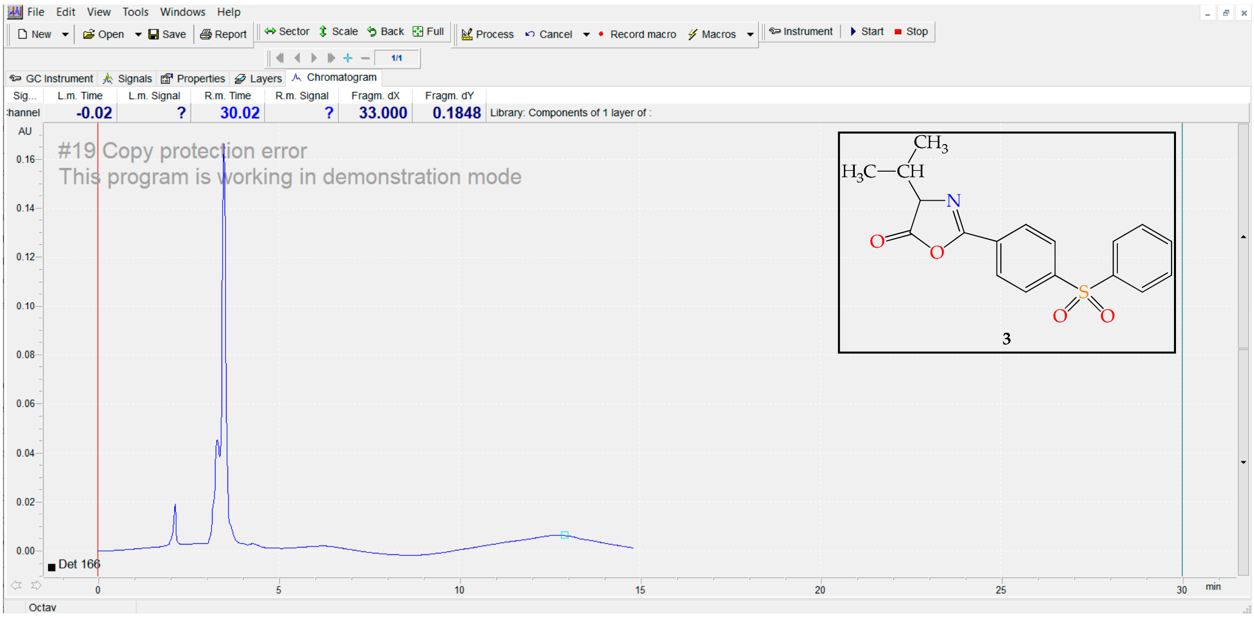

Figure S34. The RP-HPLC chromatogram of 4-isopropyl-2-[4-(phenylsulfonyl)phenyl]-1,3-oxazol-5(4*H*)-one **3**.

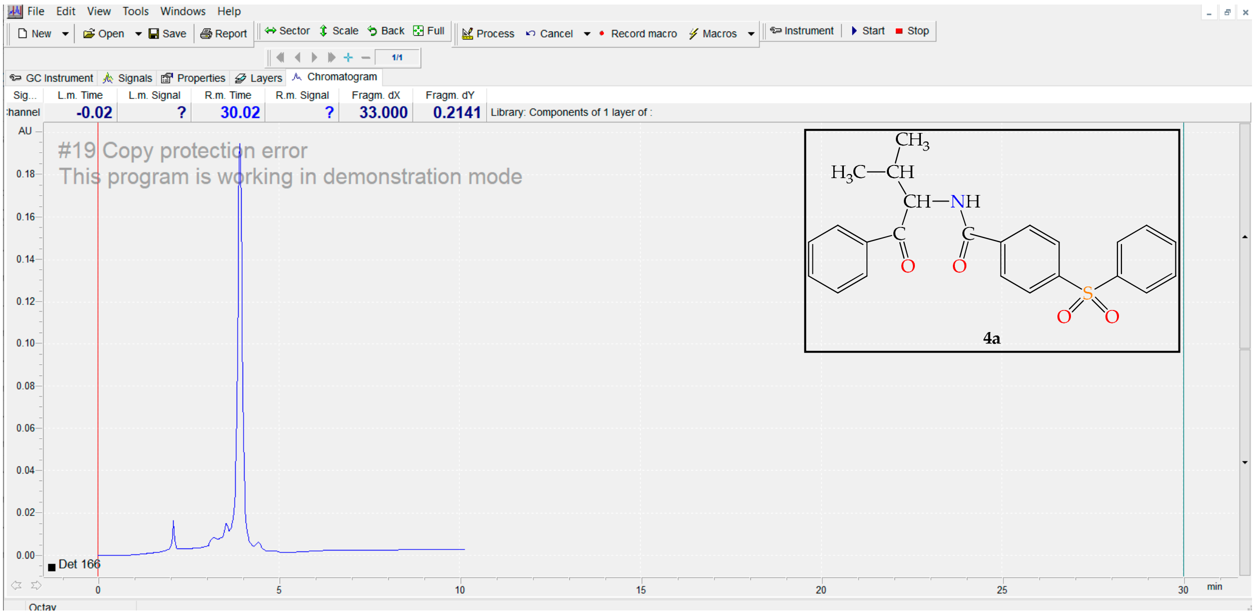

Figure S35. The RP-HPLC chromatogram of *N*-(3-methyl-1-oxo-1-phenylbutan-2-yl)-4-(phenylsulfonyl)benzamide **4a**.

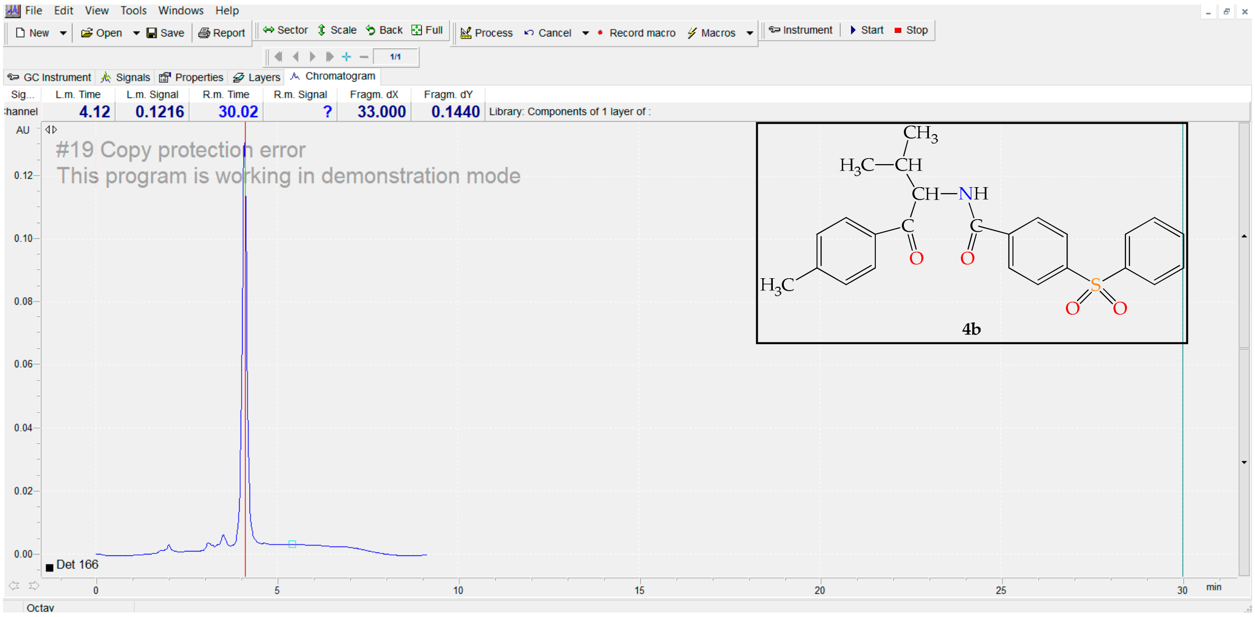

Figure S36. The RP-HPLC chromatogram of *N*-[3-methyl-1-oxo-1-(*p*-tolyl)butan-2-yl]-4-(phenylsulfonyl)benzamide **4b**.

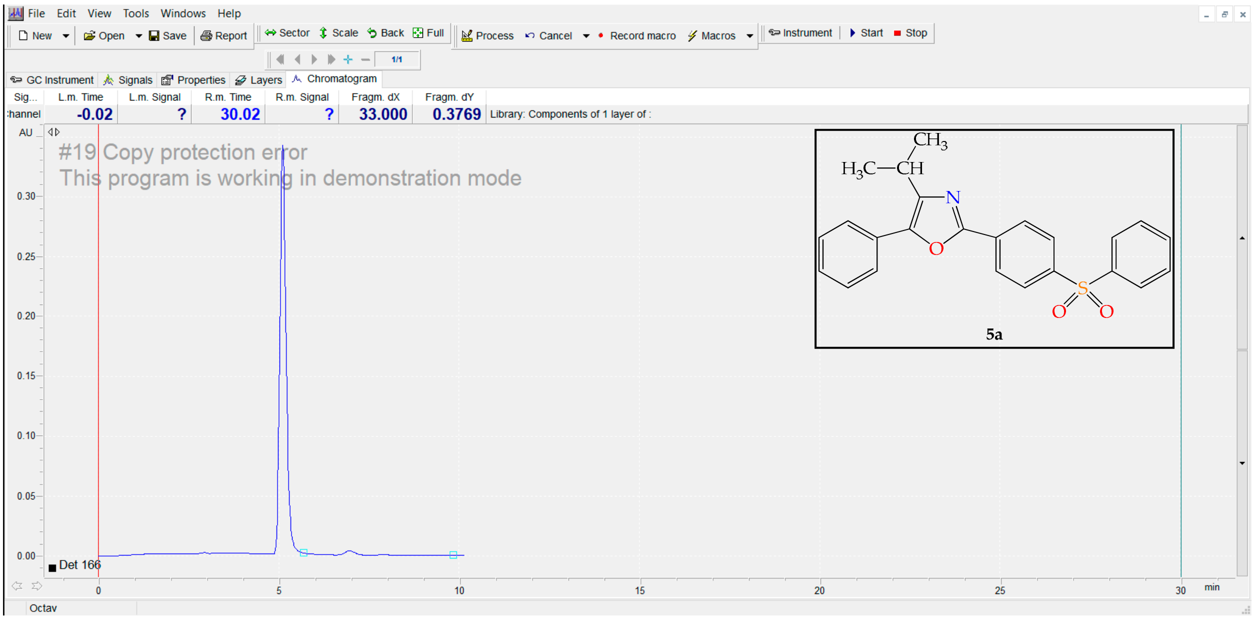

Figure S37. The RP-HPLC chromatogram of 4-isopropyl-5-phenyl-2-[4-(phenylsulfonyl)phenyl]-1,3-oxazole **5a**.

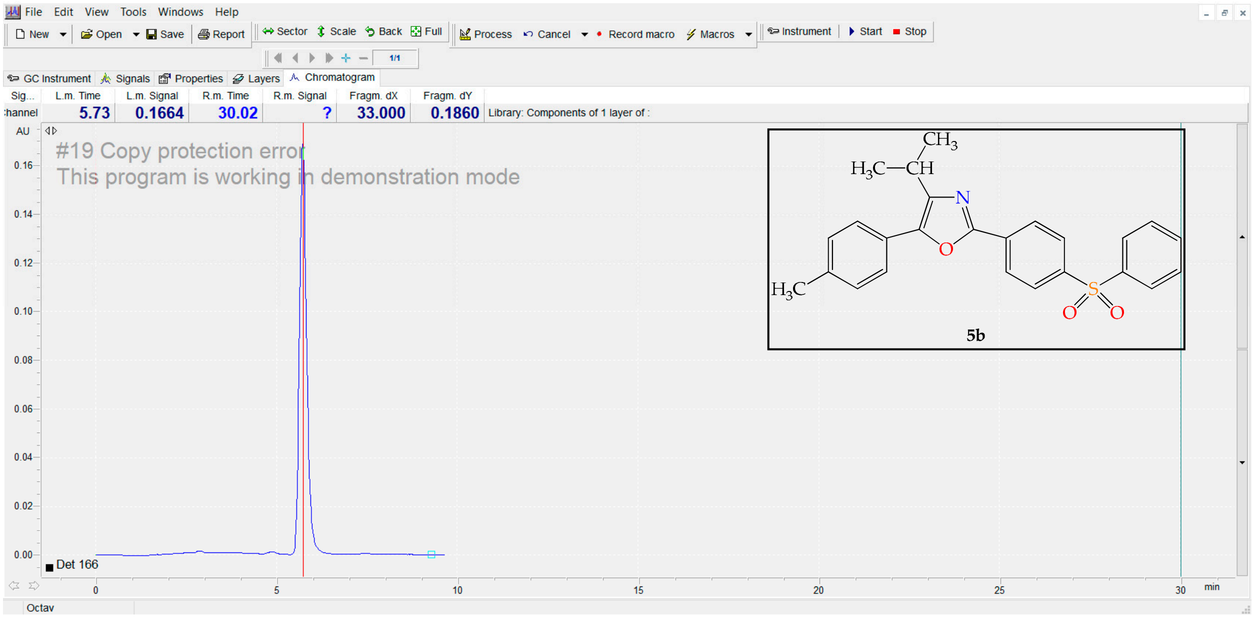

Figure S38. The RP-HPLC chromatogram of 4-isopropyl-2-[4-(phenylsulfonyl)phenyl]-5-(*p*-tolyl)-1,3-oxazole **5b**.

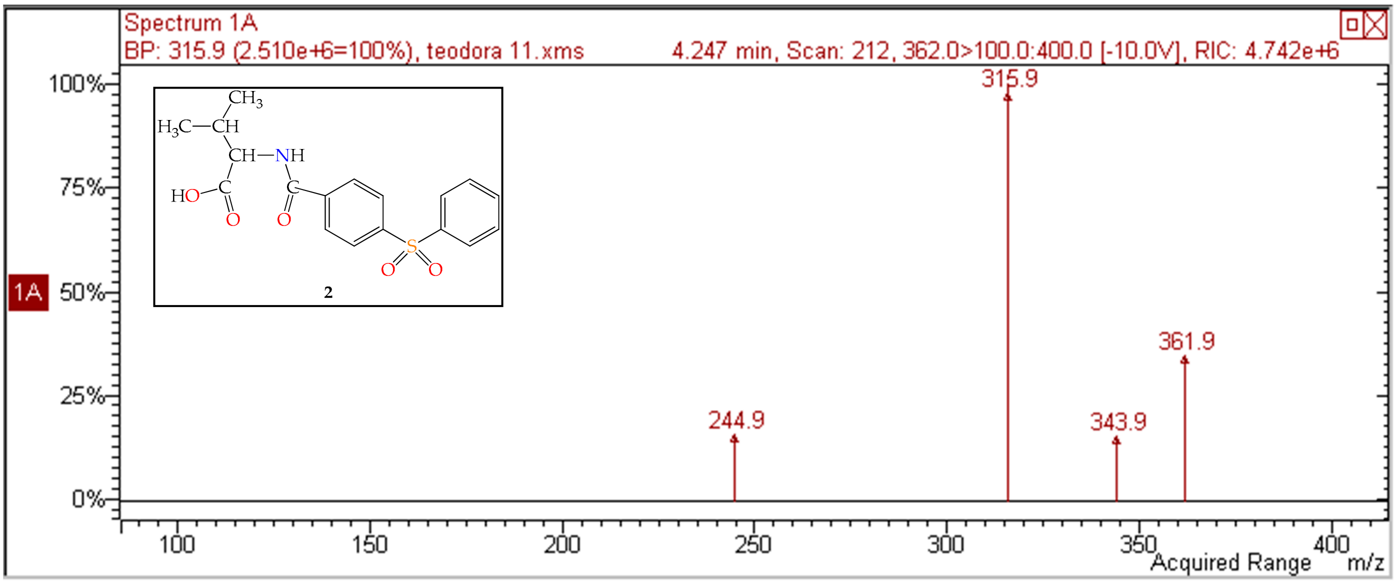

Figure S39. The +ESI-MS/MS spectrum of 3-methyl-2-[4-(phenylsulfonyl)benzamido]butanoic acid **2** (parent ion $[M+H]^+$ *m/z* = 362, fragments at -10 V collision energy).

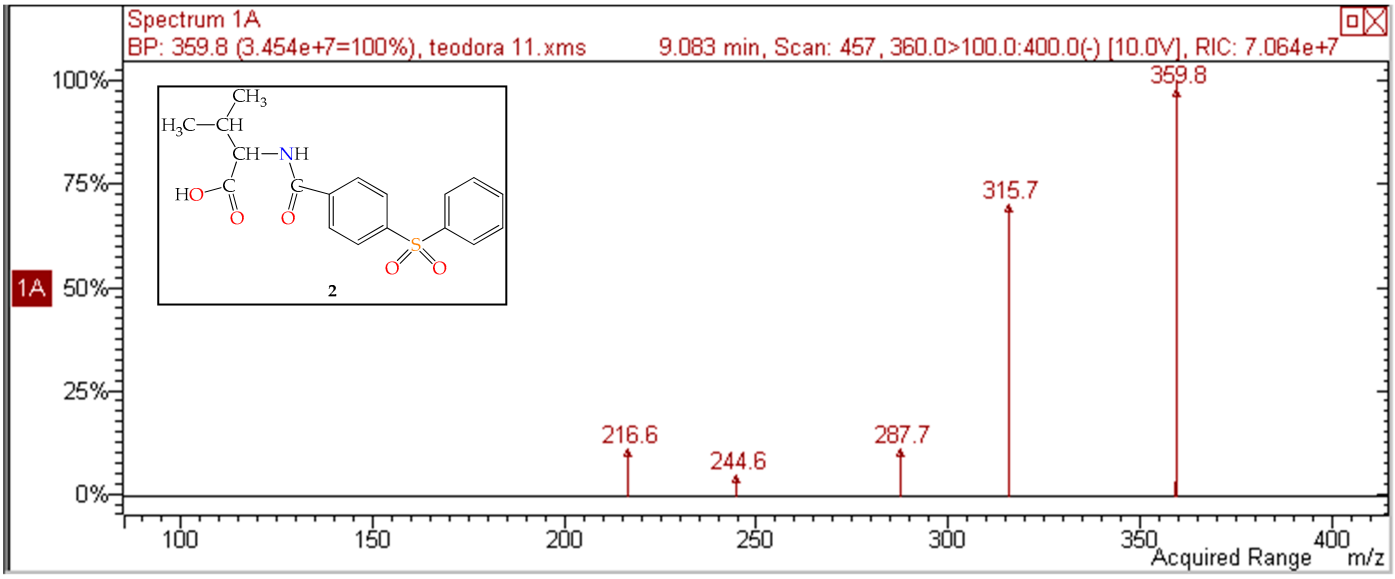

Figure S40. The -ESI-MS/MS spectrum of 3-methyl-2-[4-(phenylsulfonyl)benzamido]butanoic acid **2** (parent ion $[M-H]^-$ *m/z* = 360, fragments at +10 V collision energy).

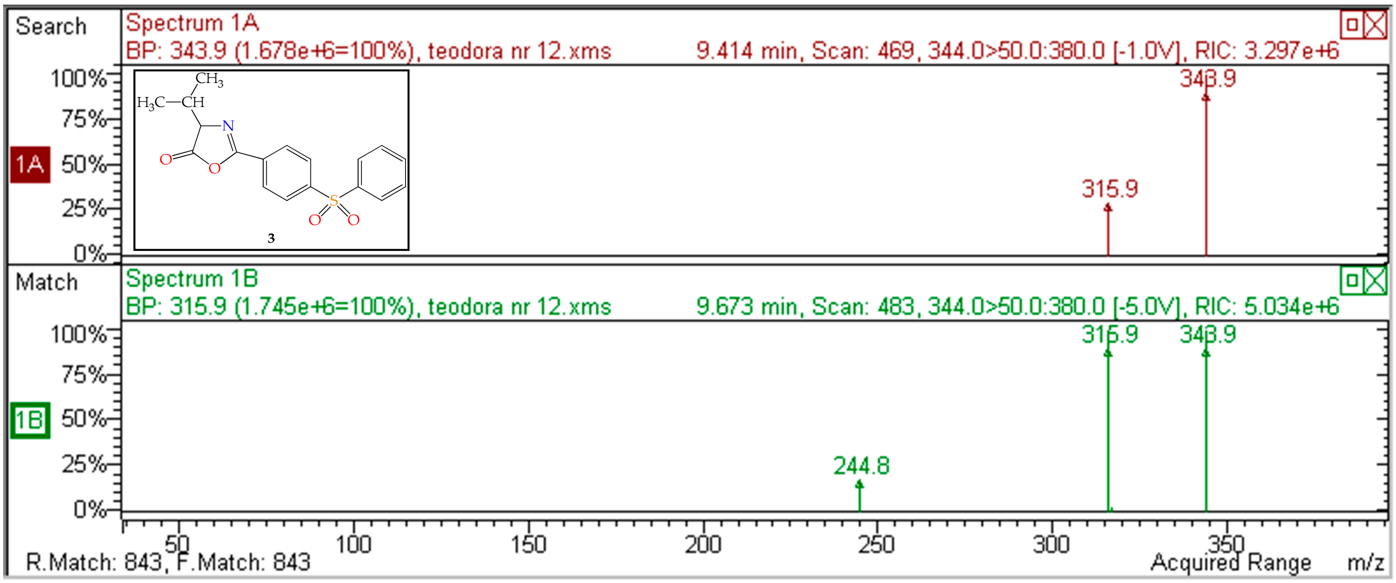

Figure S41. The +ESI-MS/MS spectrum of 4-isopropyl-2-[4-(phenylsulfonyl)phenyl]-1,3-oxazol-5(4*H*)-one **3** (parent ion $[M+H]^+$ m/z = 344, fragments at -1 V and -5 V collision energy).

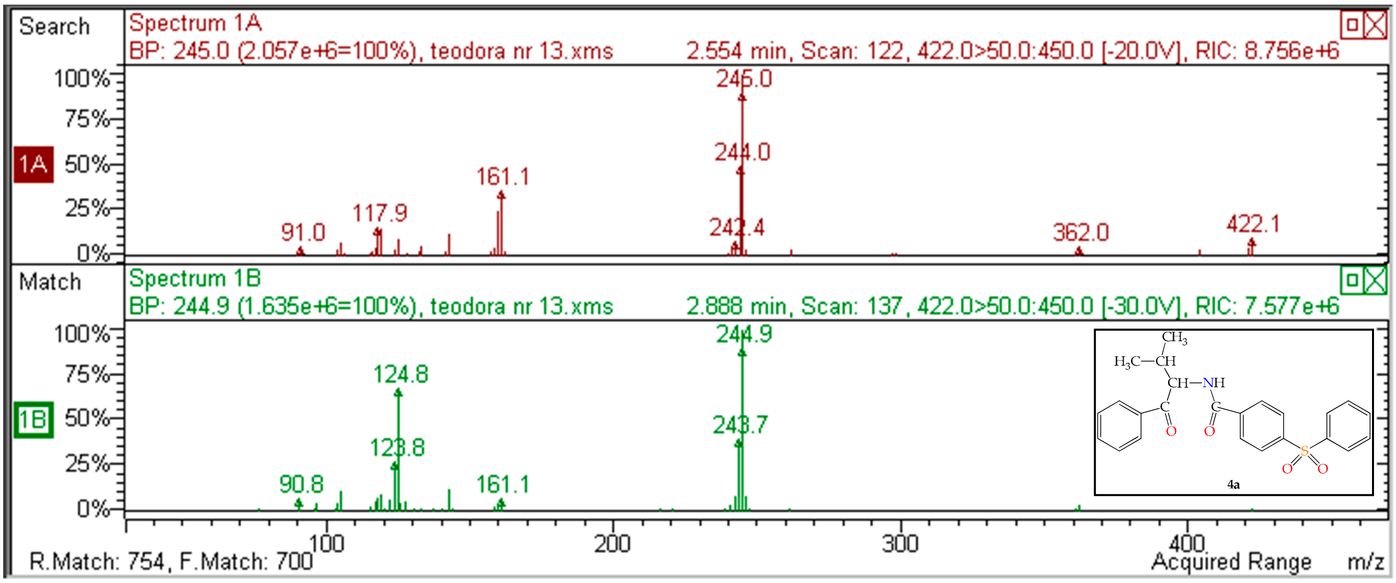

Figure S42.1. The +ESI-MS/MS spectrum of *N*-(3-methyl-1-oxo-1-phenylbutan-2-yl)-4-(phenylsulfonyl)benzamide **4a** (parent ion $[M+H]^+$ m/z = 422, fragments at -20 V and -30 V collision energy).

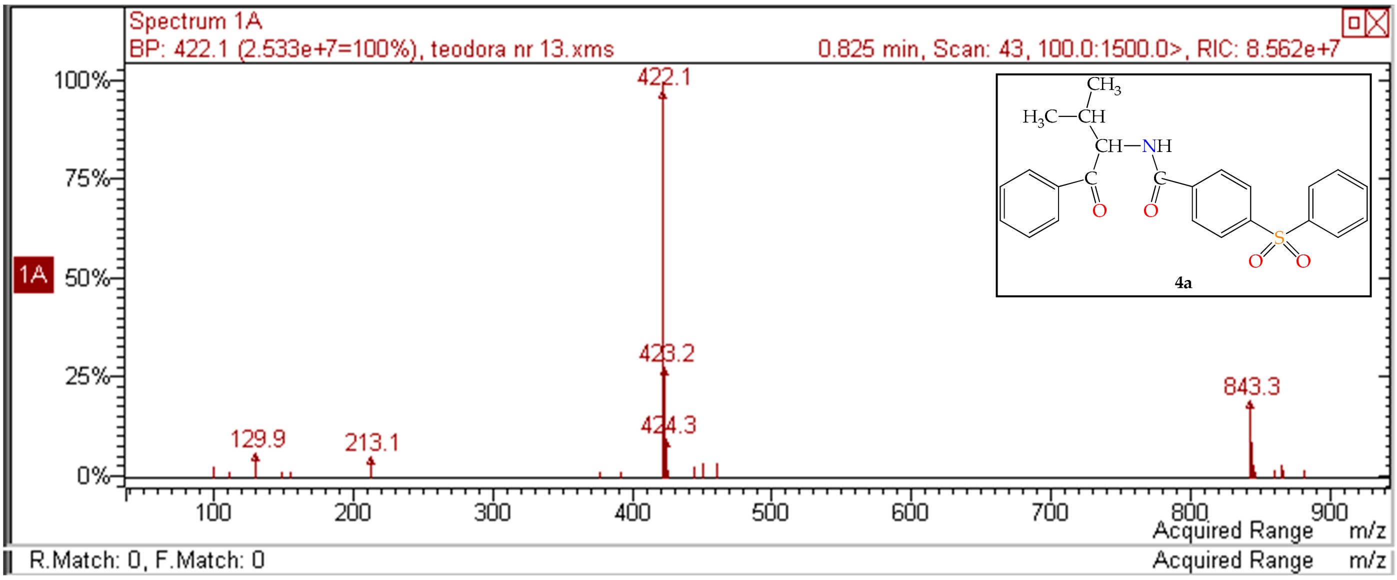

Figure S42.2. The +ESI-MS spectrum of *N*-(3-methyl-1-oxo-1-phenylbutan-2-yl)-4-(phenylsulfonyl)benzamide **4a** (scan range *m/z* = 100-1000 a.m.u., $[M+H]^+$ *m/z* = 422, $[2M+H]^+$ *m/z* = 843).

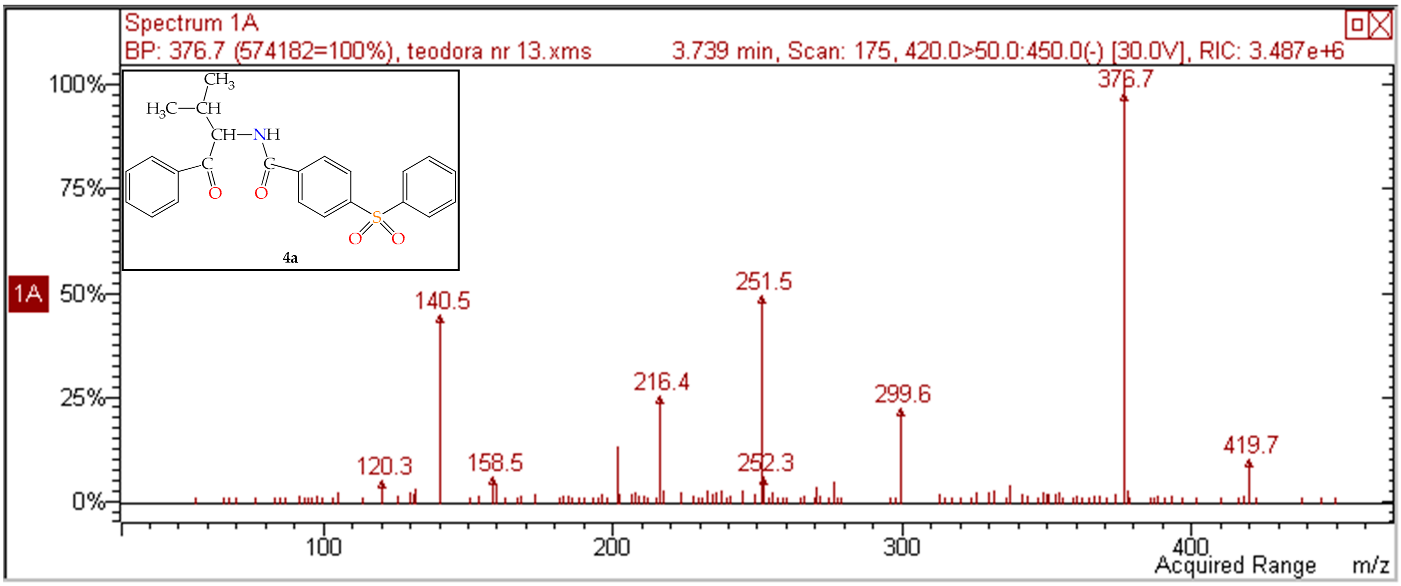

Figure S43.1. The -ESI-MS/MS spectrum of *N*-(3-methyl-1-oxo-1-phenylbutan-2-yl)-4-(phenylsulfonyl)benzamide **4a** (parent ion $[M-H]^-$ *m/z* = 420, fragments at +30 V collision energy).

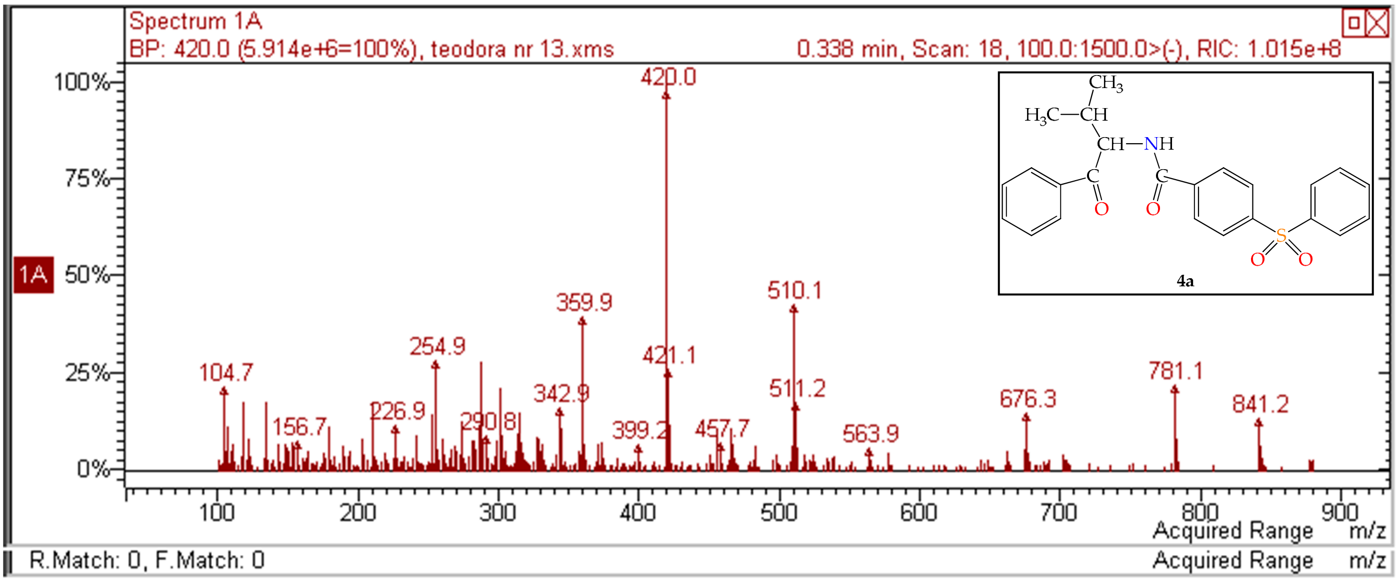

Figure S43.2. The -ESI-MS spectrum of *N*-(3-methyl-1-oxo-1-phenylbutan-2-yl)-4-(phenylsulfonyl)benzamide **4a** (scan range *m/z* = 100-1000 a.m.u., [M-H]$^-$ *m/z* = 420, [2M-H]$^-$ *m/z* = 841)

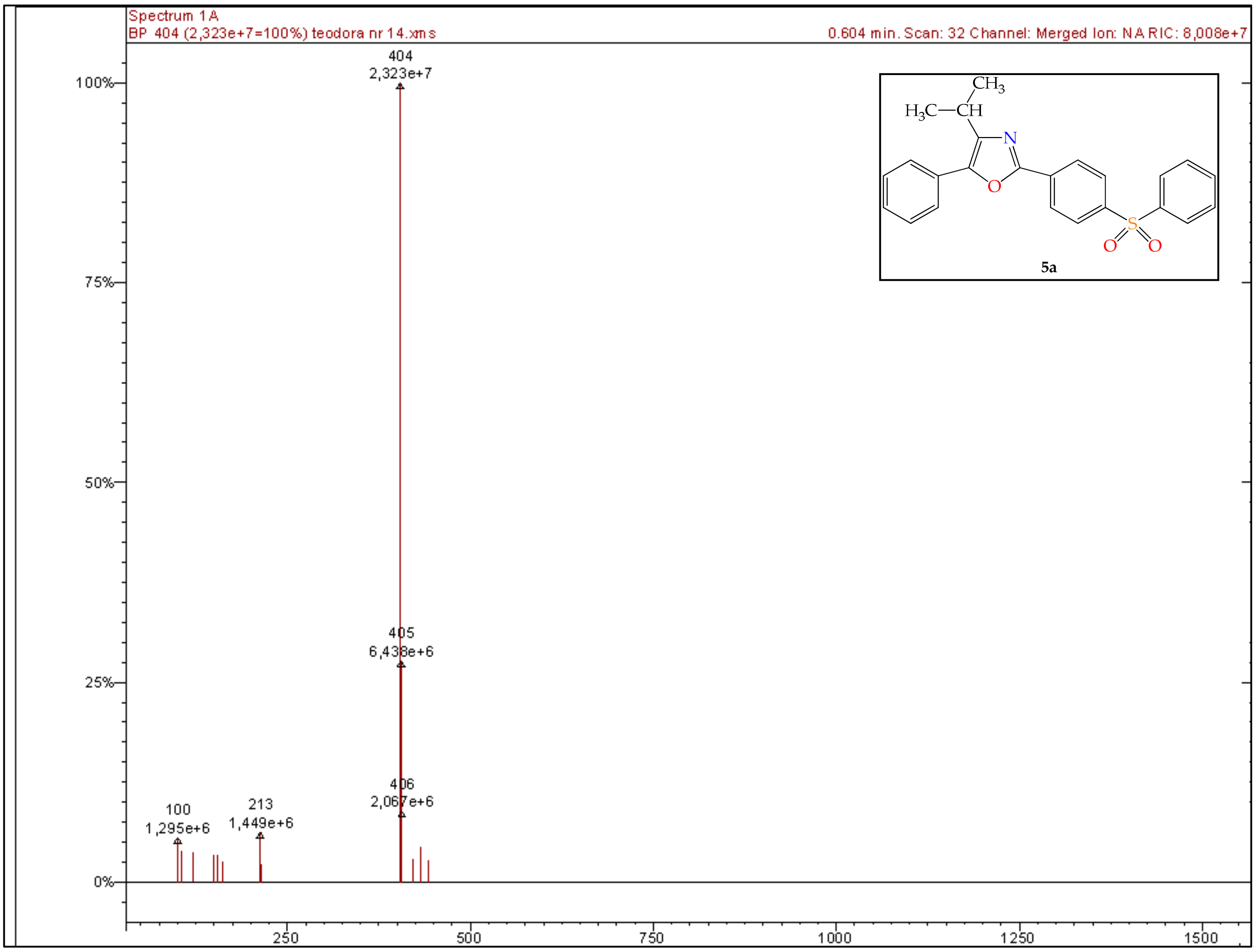

Figure S44.1. The +ESI-MS spectrum of 4-isopropyl-5-phenyl-2-[4-(phenylsulfonyl)phenyl]-1,3-oxazole **5a** (scan range *m/z* = 150-1500 a.m.u., parent ion [M+H]$^+$ *m/z* = 404).

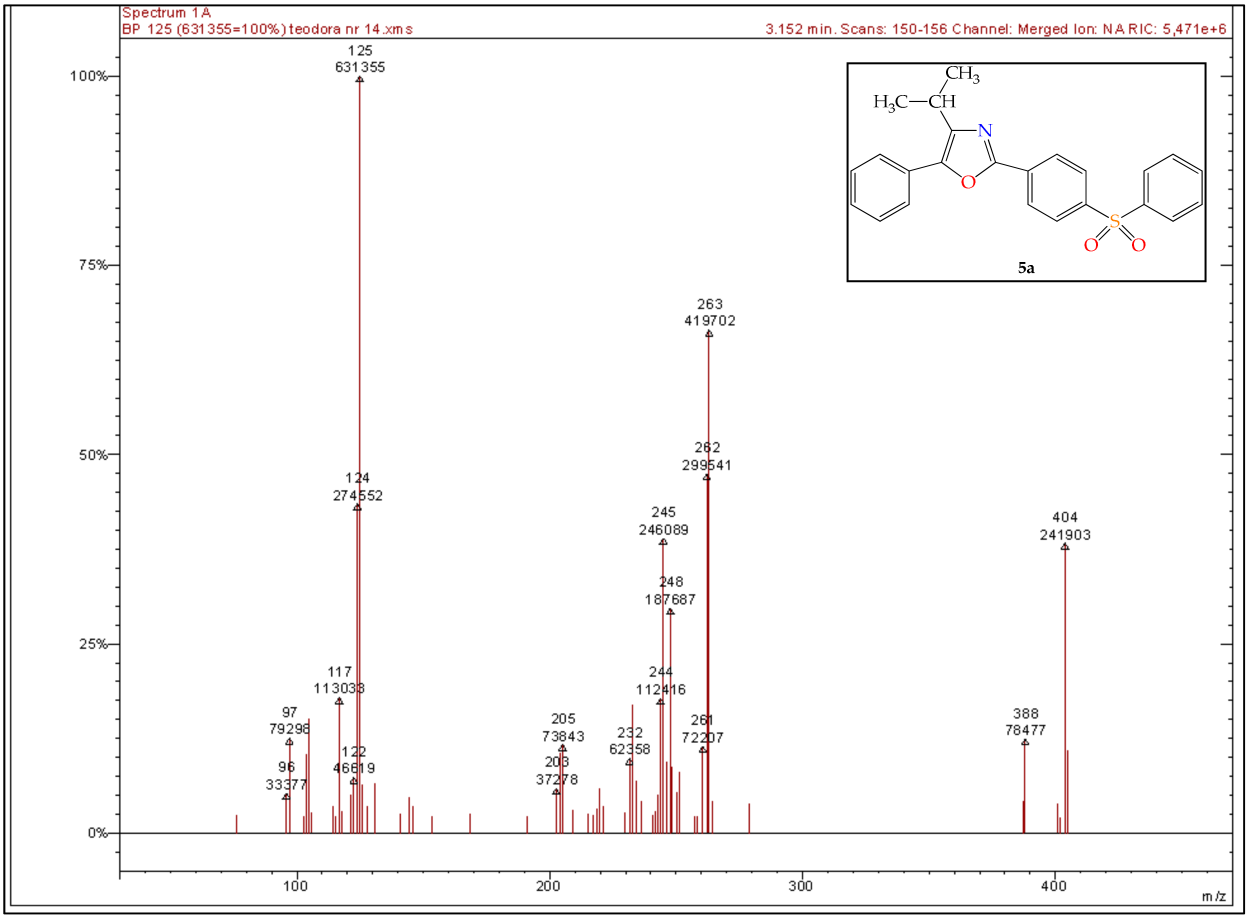

Figure S44.2 The +ESI-MS/MS spectrum of 4-isopropyl-5-phenyl-2-[4-(phenylsulfonyl)phenyl]-1,3-oxazole **5a** (parent ion $[M+H]^+$ m/z = 404, fragments at -30 V collision energy).

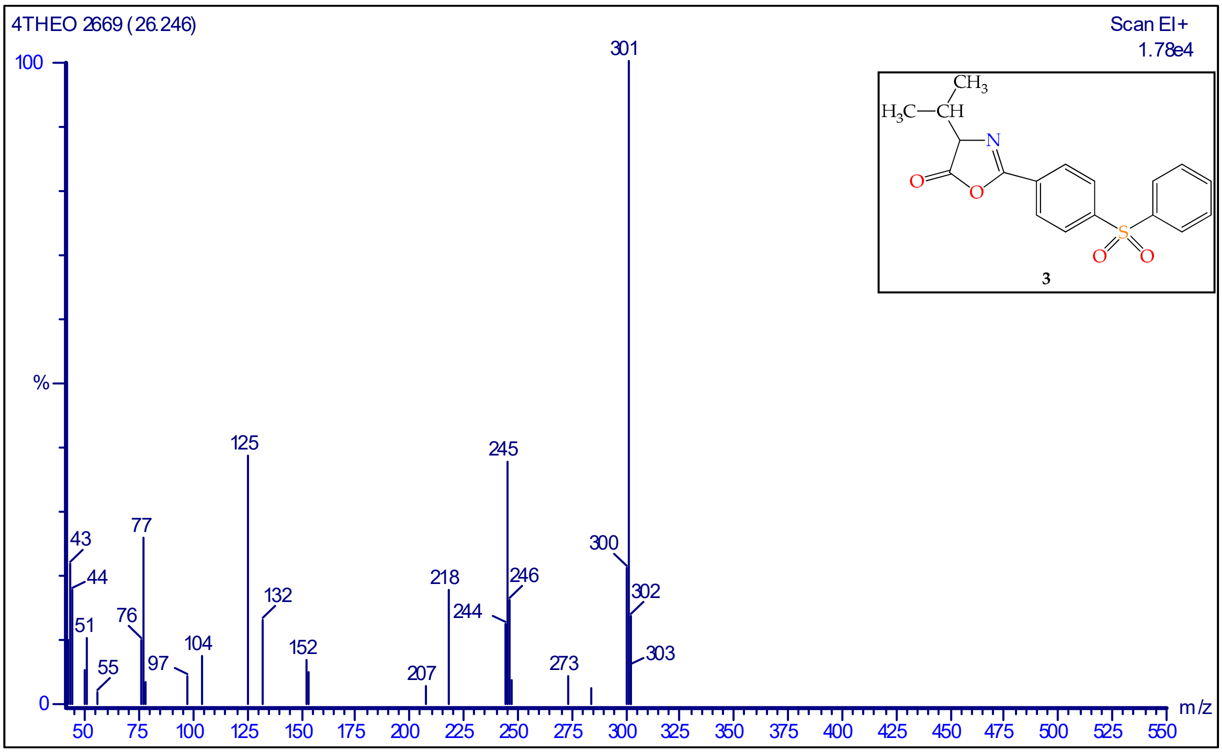

Figure S45. The GC-MS spectrum of 4-isopropyl-2-[4-(phenylsulfonyl)phenyl]-1,3-oxazol-5(4*H*)-one **3**.
